# CDC73 suppresses lung adenocarcinoma via the PTEN/AKT pathway and exhibits biomarker and functional potential in extracellular vesicles

**DOI:** 10.1038/s41420-026-03305-6

**Published:** 2026-08-14

**Authors:** Hao Dong, Yifan Zheng, Gaoming Wang, Xingyi Li, Jing Luo, Jingjing Feng, Xiaohui Zhu, Chongjun Zhong, Yiming Xu, Tian Xie, Yi Shen

**Affiliations:** 1 Department of Thoracic Surgery, Xuzhou Central Hospital, Xuzhou Clinical School of Xuzhou Medical University, Xuzhou, PR China; 2 Department of Thoracic Surgery, Affiliated Hospital 2 of Nantong University and First People’s Hospital of Nantong City, Nantong, China; 3 Department of Cardiothoracic Surgery, First Affiliated Hospital of Huzhou Normal University, Huzhou, China; 4 Department of Cardiothoracic Surgery, Nanjing Jinling Hospital, Affiliated Hospital of Medical School, Nanjing University, Nanjing, China; 5 The First Clinical Medicine School, Xuzhou Medical University, Xuzhou, China; 6 Jiangsu Key Laboratory of Molecular Medicine, Nanjing University, Nanjing, China; 7 Department of Thoracic Surgery, Zhongda Hospital, Southeast University, Nanjing, China

**Keywords:** Non-small-cell lung cancer, Cell growth

## Abstract

CDC73 is a canonical tumor suppressor; however, its biological functions in lung adenocarcinoma (LUAD) and the extracellular vesicle (EV)-mediated mechanisms through which it exerts its effects remain unclear. In this study, we systematically evaluated the diagnostic value, biological functions, and EV-mediated regulatory roles of CDC73 in LUAD. First, quantitative proteomic analysis was performed on plasma-derived EVs from six patients with early-stage LUAD and three healthy donors, followed by further validation of EV-borne CDC73 expression in plasma-derived EV samples from eight LUAD patients and eight healthy donors. We found that the level of EV-borne CDC73 protein (EV-CDC73) was significantly reduced in LUAD, indicating its potential value as a diagnostic liquid-biopsy biomarker. To define its functional role, we established LUAD cell lines stably overexpressing CDC73 (A549^OE and H1299^OE) and performed rescue experiments in combination with PTEN knockdown using siRNA. The tumor-suppressive effects of CDC73 were systematically assessed through experiments examining cell proliferation, cell-cycle progression, apoptosis, migration, and related molecular mechanisms. Meanwhile, EVs were isolated by ultracentrifugation from the conditioned media of A549, H1299, and human bronchial epithelial (HBE) cells to analyze the effects of EVs with different CDC73 levels on recipient A549 and HBE cells, and xenograft models were used for in vivo validation. The results showed that CDC73 overexpression markedly inhibited LUAD cell proliferation and migration, induced G1-phase arrest, and promoted apoptosis. Mechanistically, CDC73 exerted its tumor-suppressive effects by upregulating PTEN and inhibiting AKT activation, whereas PTEN knockdown partially reversed these effects. In vivo experiments further confirmed that CDC73 overexpression suppressed tumor growth and migration. In addition, EVs derived from LUAD cells with high CDC73 expression showed a reduced ability to induce malignant transformation-related phenotypes in HBE cells and suppressed the PTEN/AKT signaling axis; conversely, EVs derived from HBE cells with high CDC73 expression inhibited the malignant progression of LUAD cells. Collectively, CDC73 suppresses the malignant progression of LUAD through the PTEN/AKT signaling axis, while EV-borne CDC73 not only has potential diagnostic value but also directly regulates malignant phenotypes in recipient cells, highlighting its translational potential in the diagnosis and treatment of LUAD.

**Figure:**
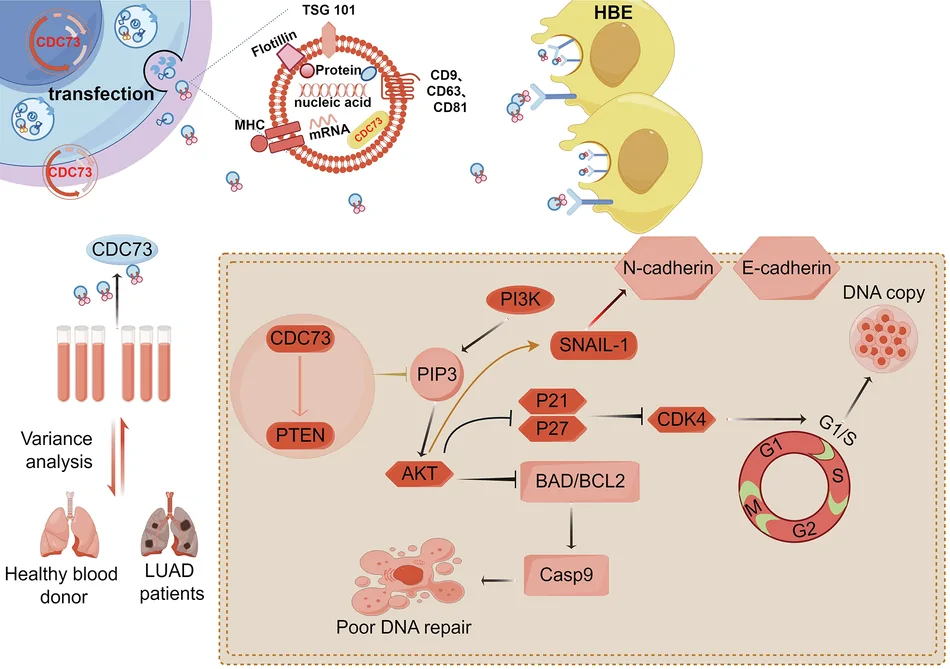

## Introduction

It is well known that lung cancer is the second most common cancer and the leading cause of cancer-related deaths [1]. Despite significant advances in diagnosis and treatment, the prognosis for lung cancer patients remains generally poor, with an overall 5-year survival rate of only 22% [2]. Non-small cell lung cancer (NSCLC) accounts for approximately 85% of all lung cancer cases, with lung adenocarcinoma (LUAD) being the most common subtype of NSCLC, comprising about 40% of all lung cancer cases [3]. Therefore, understanding the molecular mechanisms underlying the development of LUAD and identifying early diagnostic biomarkers and effective therapeutic targets are crucial to improving the survival rate of lung cancer patients.

CDC73, also known as HPRT2, is a tumor suppressor gene that encodes the protein parafibromin, which is involved in regulating cell proliferation and gene expression [4, 5]. Studies have shown that CDC73 expression is primarily localized in the nucleus and cytoplasm [6], and it plays a crucial role in the pathogenesis and prognosis of several cancers, including parathyroid [7], breast [8], liver [9], colorectal cancer (CRC) [10], ovarian epithelium [11], urinary tract [12], gastric cancer [13], and osteosarcoma [14]. Research has demonstrated that the CDC73 protein can bind to RNA polymerase II, thereby inhibiting cell cycle progression [5]. CDC73 also inhibits osteosarcoma cell proliferation via the PI3K/AKT signaling pathway and promotes the activation of caspase-9, inducing apoptosis [14]. Moreover, overexpressed CDC73 acts as an anti-proliferative nuclear protein that can trigger G1 cell cycle arrest [15, 16]. In ovarian cancer, increased CDC73 expression induces apoptosis and inhibits cell growth, migration, and invasion [11]. However, the biological functions and molecular mechanisms of CDC73 in LUAD remain unclear. Our evidence suggests that CDC73 plays a role in suppressing the malignant progression of LUAD cells.

Extracellular vesicles (EVs) represent an emerging field in cancer research, where proteins and miRNAs contained in tumor-derived EVs can serve as biomarkers for cancer diagnosis and prognosis. EVs mediate intercellular communication between cancer cells and normal cells by delivering functional substances locally and remotely, including RNA, DNA, proteins, and lipids [17, 18]. These substances are distributed throughout the body via transport systems and delivered to recipient cells, influencing their biological responses and potentially promoting or inhibiting certain diseases [19, 20], including lung cancer [21]. Recent studies have reported that miRNAs and proteins contained in tumor cell-derived EVs can promote the malignant transformation of normal cells [22–24]. This study, through quantitative proteomics analysis, identified significant differences in the CDC73 protein in extracellular vesicles from the plasma of lung adenocarcinoma patients and healthy donors. It further explores the effects of CDC73 on cell proliferation, apoptosis, and migration in lung adenocarcinoma cells, as well as the underlying mechanisms involved.

Previous studies have reported that tumor cell-derived exosomes contain miRNAs and proteins that can promote the malignant transformation of normal cells [24–28]. In our study, we employed quantitative proteomics to detect proteins in extracellular vesicles from plasma samples of six lung adenocarcinoma patients and three healthy controls. However, the biological effects of CDC73 within EVs in lung adenocarcinoma remain unclear. Our research aims to identify potential diagnostic markers for lung adenocarcinoma and to investigate whether intercellular communication is influenced by CDC73 in extracellular vesicles, which is relevant to the progression of lung adenocarcinoma.

## Results

### Characterization of plasma extracellular vesicles and identification of differentially expressed proteins between early-stage lung adenocarcinoma patients and healthy donors

**Fig. 1:**
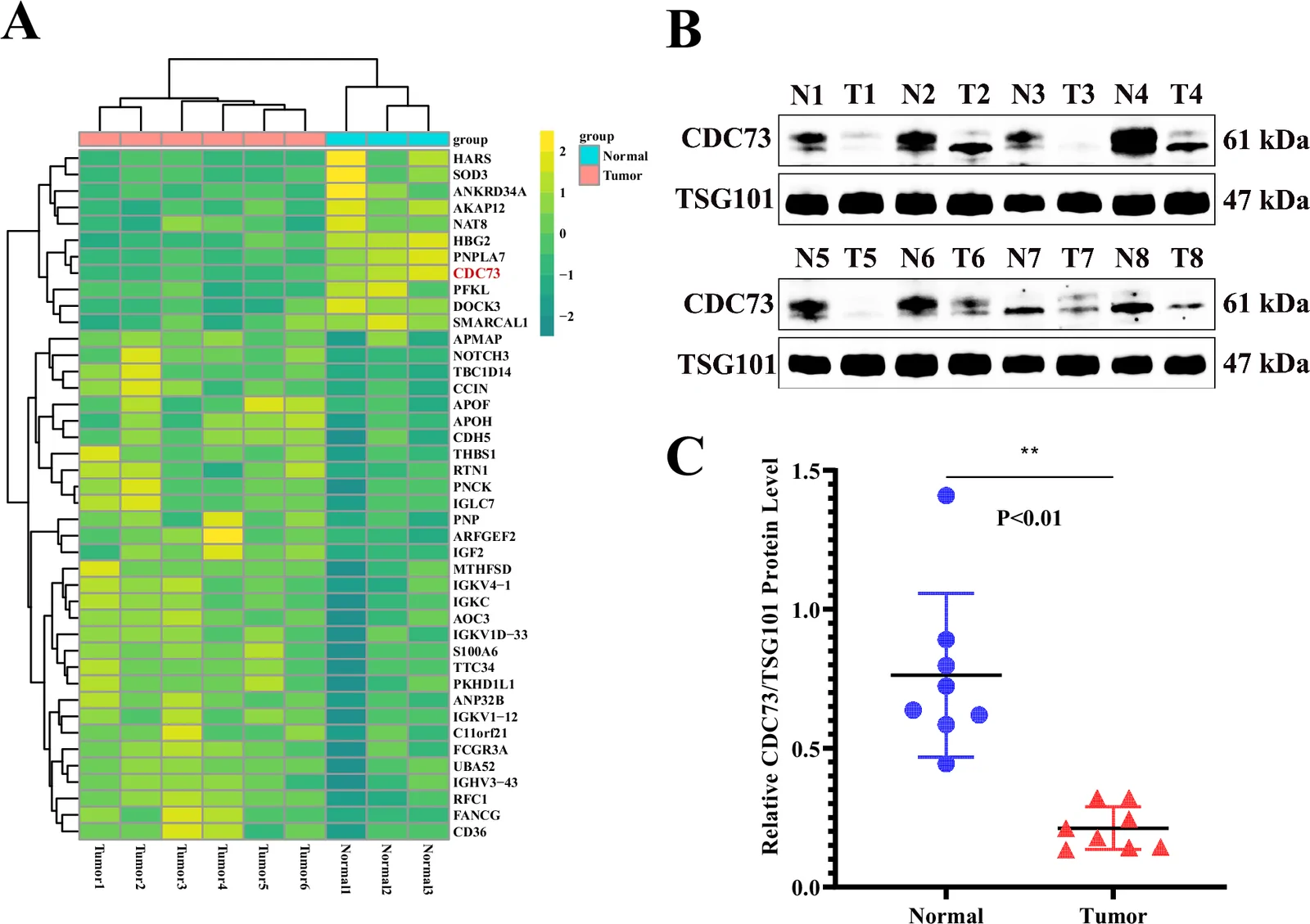
Differentially expressed proteins (DEPs) detected in plasma small extracellular vesicles (sEVs) from patients with early-stage lung adenocarcinoma and healthy donors. Quantitative proteomics revealed downregulation of CDC73 in patient-derived sEVs. **A** Quantitative proteomic analysis of DEPs in plasma sEVs from healthy volunteers (*n* = 3) and patients with primary LUAD (*n* = 6). DEPs were defined as |log2FC| > 1 with *p* < 0.05; yellow indicates upregulation and green indicates downregulation. **B**, **C** Western blot validation of CDC73 expression in plasma sEVs from healthy volunteers (*n* = 8) and patients with primary LUAD (*n* = 8). **p < 0.01).

Proteins within or embedded on the membrane of extracellular vesicles (EVs) are key cargoes that not only reflect the physiological state of the parent cells but also play critical roles in intercellular communication. Proteins in EVs are known to contribute to both the promotion and suppression of tumor progression [29]. To investigate the biological functions of proteins in EVs during the development of early-stage lung adenocarcinoma (LUAD), we extracted EVs from the plasma samples of LUAD patients and healthy donors using ultracentrifugation. To confirm that the isolated material was indeed EVs, we employed several characterization methods according to the guidelines provided in the 2023 Minimal Information for Studies of Extracellular Vesicles (MISEV2023). Firstly, transmission electron microscopy (TEM) showed that the EVs had a typical cup-shaped morphology. Nanoparticle tracking analysis (NTA) revealed that the diameter of these EVs ranged from 30 to 150 nm (Fig. S5D, E). Through quantitative proteomics analysis, using the cutoff criteria of “log2FC absolute value > 1 and *p* value < 0.05,” we identified 42 differentially expressed proteins in the plasma EVs from early-stage LUAD patients (*n* = 6) and healthy donors (*n* = 3) (Fig. 1A). Since EV proteins generally correspond to those of the parent cells, we analyzed the genes encoding these 42 proteins (https://www.genecards.org) and found that the tumor suppressor protein CDC73 was consistently present. To validate the proteomics results, we performed Western blot analysis on a small sample set to assess CDC73 expression in the plasma EVs from early-stage LUAD patients (*n* = 8) and healthy donors (*n* = 8). The results indicated that CDC73 expression in the plasma EVs of healthy donors was higher than in those of early-stage LUAD patients (Fig. 1B, C). This finding is consistent with the results from the quantitative proteomics sequencing.

### CDC73 inhibits LUAD cell proliferation in vitro and induces G1 phase cell cycle arrest

**Fig. 2:**
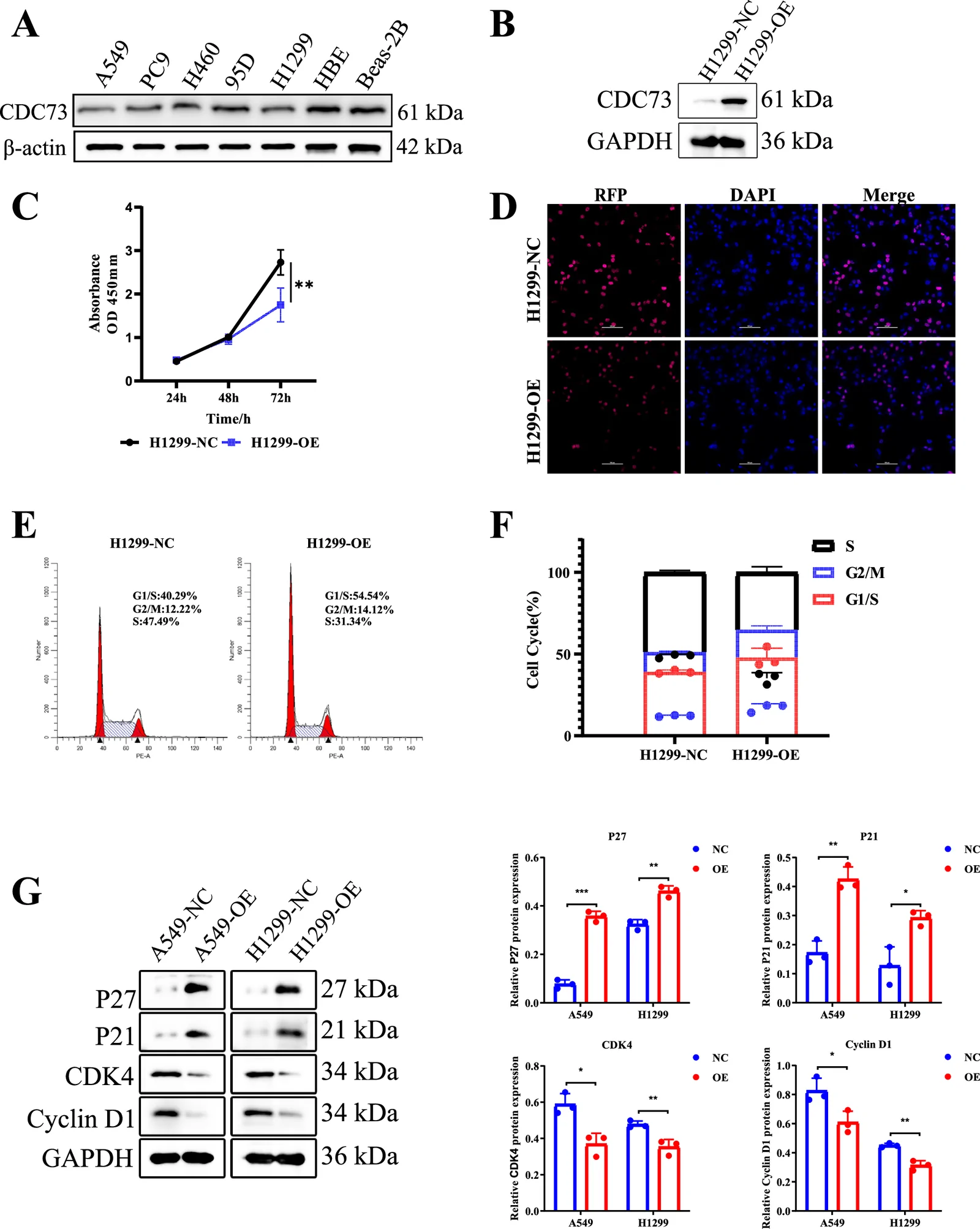
CDC73 suppresses LUAD cell proliferation in vitro and modulates cell-cycle progression. **A** Western blot analysis showing differential CDC73 expression between normal pulmonary epithelial cell lines and lung cancer cell lines. **B** Lentiviral transduction of H1299 lung adenocarcinoma cells; Western blotting confirming CDC73 overexpression. **C** CCK-8 assay comparing proliferative capacity between control and CDC73-overexpressing H1299 cells. **D** EdU incorporation assay assessing proliferation in control versus CDC73-overexpressing H1299 cells. Scale bar, 100 μm. **E** Flow cytometric analysis of cell-cycle distribution in H1299 cells with and without CDC73 overexpression. **F** Quantitative analysis of cell-cycle distribution in H1299 cells with and without CDC73 overexpression. **G** Western blot analysis of cell-cycle regulatory proteins following CDC73 overexpression, with densitometric quantification.

CDC73, also known as HPRT2, is a tumor suppressor gene that plays a role in inhibiting cancer progression in various cancers. However, its function in lung adenocarcinoma (LUAD) remains unclear. First, we investigated CDC73 protein expression in five lung cancer cell lines (A549, PC9, H460, 95D, and H1299) and two human normal bronchial epithelial cell lines (HBE and Beas-2B) using Western blot (Fig. 2A). The results showed that CDC73 expression in normal bronchial epithelial cells (HBE and Beas-2B) was significantly higher than in LUAD cells (A549, PC9, and H1299). Next, to explore whether CDC73 overexpression affects the function of LUAD cells, we infected A549 and H1299 cells, which have low CDC73 expression, with lentivirus to overexpress CDC73. The transfection efficiency of the CDC73-overexpressing cells (A549-OE and H1299-OE) was confirmed via Western blot, showing a significant difference compared to the control groups (A549-NC and H1299-NC) (Figs. 2B and S1A). To determine the effects of CDC73 on LUAD cell proliferation and cell cycle progression, we conducted proliferation-related assays in vitro. The CCK-8 assay results indicated that CDC73 overexpression led to a decrease in the viability of A549 and H1299 cells compared to the control group (*p < 0.05; **p < 0.01, Figs. 2C and S1B). Additionally, we used the EdU-594 assay to label newly synthesized DNA and Hoechst 33342 to stain the total DNA as background, showing that CDC73 overexpression inhibited the proliferation of LUAD cells (Figs. 2D and S1C). Flow cytometric cell cycle analysis revealed that CDC73 overexpression inhibited cell cycle progression in LUAD cells, resulting in G1 phase arrest (Figs. 2E, F and S1D). Furthermore, Western blot analysis of G1/S phase-related proteins showed that CDC73 overexpression significantly upregulated the expression of P21 and P27 while downregulating Cyclin D1 and CDK4 (Fig. 2G). These results indicate that CDC73 overexpression suppresses the proliferation of LUAD cells and induces G1 phase cell cycle arrest.

### CDC73 overexpression inhibits LUAD cell invasion and migration and induces apoptosis

**Fig. 3:**
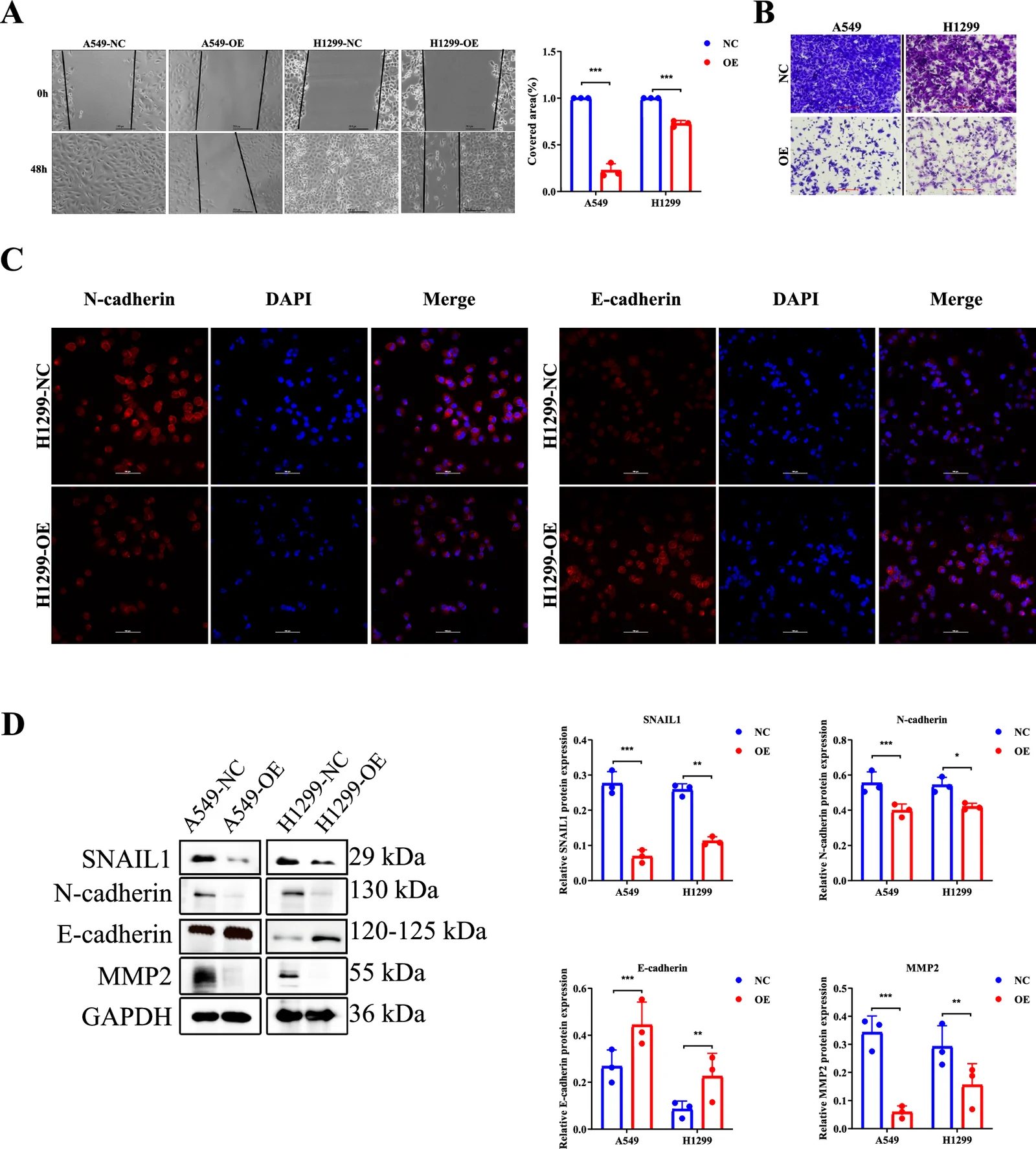
CDC73 suppresses LUAD cell migration in vitro and is associated with EMT. **A** Wound-healing assays evaluating migration of A549 and H1299 cells after lentiviral overexpression of CDC73, with quantitative analysis. **B** Transwell migration assays assessing A549 and H1299 cells following CDC73 overexpression. **C** Confocal immunofluorescence staining showing N-cadherin and E-cadherin signal intensities in H1299 cells with control versus CDC73 overexpression. Scale bar, 100 μm. **D** Western blot analysis of EMT-related proteins following CDC73 overexpression, with densitometric quantification. *p < 0.05; **p < 0.01; ***p < 0.001.

Cancer cell invasion and migration are closely associated with cancer progression and metastasis [30]. Through the wound healing assay, we found that CDC73 overexpression effectively inhibited the migration ability of LUAD cells (***p < 0.001, Fig. 3A). The Transwell assay similarly demonstrated that high expression of CDC73 significantly impeded the invasion of LUAD cells (Fig. 3B). Previous studies have shown that epithelial-mesenchymal transition (EMT) not only promotes cell invasion and migration but also enhances tumor stemness and resistance to chemotherapy, making it one of the core mechanisms of tumor progression. One of the most critical features of EMT is the loss of E-cadherin gene expression, which causes dysfunction in the cell adhesion system, leading to cancer cell invasion and metastasis [31]. In this experiment, we performed immunofluorescence staining using monoclonal antibodies against E-cadherin (red) and N-cadherin (red) to verify the changes in EMT marker protein expression. The results showed that the CDC73-overexpressing A549 and H1299 cells (A549-OE and H1299-OE) exhibited upregulated E-cadherin fluorescence and downregulated N-cadherin fluorescence compared to the control groups (A549-NC and H1299-NC) (Figs. 3C and S2A). Furthermore, we used a Western blot to analyze the expression of EMT-related proteins. The results revealed that CDC73 overexpression led to the downregulation of N-cadherin, SNAIL1, and MMP2, along with the upregulation of E-cadherin (*p < 0.05; **p < 0.01; ***p < 0.001, Fig. 3D). These results are consistent with the findings from the immunofluorescence experiments. Apoptosis is a key mechanism by which the body maintains normal physiological functions, and the inhibition of apoptosis is often closely related to cancer development and progression [32]. To investigate the effects of CDC73 overexpression on apoptosis in LUAD cells, we analyzed apoptosis changes in CDC73-transfected LUAD cells using flow cytometry. The results showed that the CDC73-overexpressing A549 and H1299 cells (A549-OE and H1299-OE) exhibited significantly increased apoptosis rates compared to the control groups (A549-NC and H1299-NC) (*p < 0.05; **p < 0.01, Fig. S3A-3B). Western blot analysis of apoptosis-related proteins further demonstrated that CDC73 overexpression led to the upregulation of caspase-9 and the pro-apoptotic protein BAD, along with a decrease in the anti-apoptotic protein Bcl-2 (*p < 0.05; **p < 0.01, Fig. S3C). In summary, our data indicate that CDC73 overexpression can inhibit LUAD cell invasion and migration by suppressing the development of EMT, and CDC73 also has the ability to induce apoptosis in lung adenocarcinoma cells.

### CDC73 upregulates PTEN expression and inhibits the PTEN/AKT signaling pathway

**Fig. 4:**
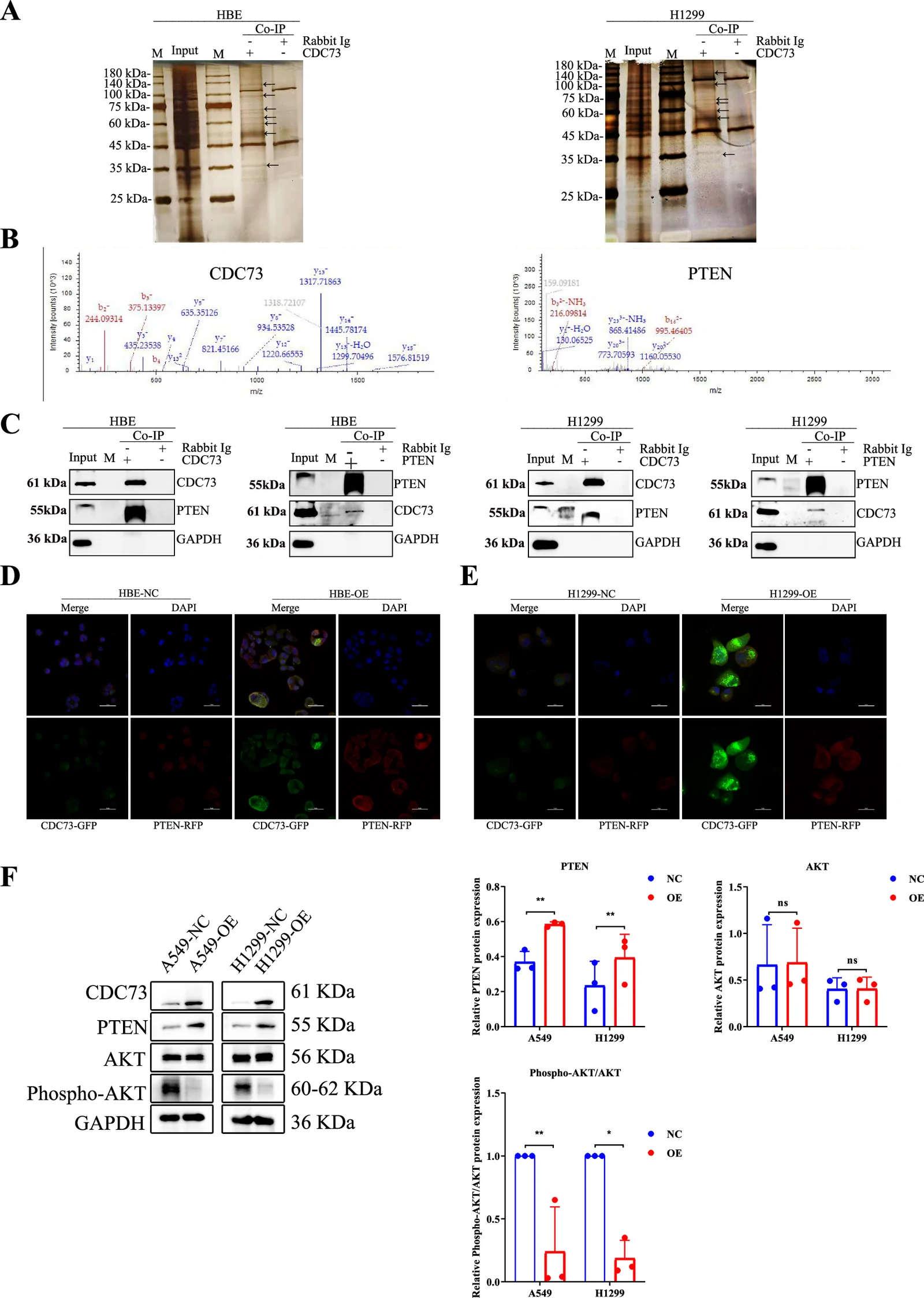
CDC73 regulates PTEN expression to suppress the PI3K/AKT signaling pathway. **A** SDS-PAGE separation of proteins co-immunoprecipitated (co-IP) with CDC73 from HBE and H1299 cell lysates, visualized by silver staining. Black arrows indicate proteins pulled down by the anti-CDC73 antibody. **B** LC–MS/MS identification of peptide fragments corresponding to CDC73 and PTEN. **C** Co-IP analysis of total protein extracts from HBE and H1299 cells demonstrating the interaction between CDC73 and PTEN. **D**, **E** Immunofluorescence (IF) staining illustrating the relationship between CDC73 and PTEN in H1299 lung adenocarcinoma cells and HBE bronchial epithelial cells. **F** Western blot analysis showing changes in PTEN protein levels upon CDC73 overexpression, with densitometric quantification. *p < 0.05; **p < 0.01; ns, not significant.

The PI3K/AKT/mTOR (PAM) signaling pathway is a highly conserved signal transduction network in eukaryotic cells that promotes cell survival, growth, and cell cycle progression [33]. To explore the downstream signaling pathways regulated by CDC73, we performed SDS-PAGE to separate CDC73 pull-down protein products from HBE and H1299 cells and observed the results via silver staining. The black arrows indicate proteins pulled down by the CDC73 antibody during co-immunoprecipitation (co-IP), with molecular weights of 140, 100, 65, 61, 55, 47, and 35 kDa (Fig. 4A). Peptide identification through mass spectrometry, followed by database comparison, confirmed that CDC73 interacts with the protein PTEN (Fig. 4B). Using the STRING database to predict the CDC73 protein–protein interaction network, we found that CDC73 may indirectly regulate PTEN expression via CTNNB1 (Fig. S2B). We further validated this interaction between CDC73 and PTEN through co-IP using total protein samples from HBE and H1299 cells (Figs. 4C and S2C). To further confirm the interaction, immunofluorescence analysis showed that CDC73 and PTEN were colocalized in both H1299 lung adenocarcinoma cells and normal bronchial epithelial cells. Additionally, we observed that the fluorescence intensity of PTEN was significantly increased in the CDC73 overexpression group (HBE-OE and H1299-OE) compared to the control group (HBE-NC and H1299-NC), indicating that CDC73 upregulation promotes PTEN expression (Fig. 4D, E). The PI3K/PIP3 signaling pathway is primarily terminated by the tumor suppressor PTEN, which dephosphorylates PIP3, converting it back to PIP2. Therefore, PTEN is a critical negative regulator of the PAM pathway that influences cell growth and survival, while the loss of PTEN leads to sustained output of intracellular signals [34]. To investigate whether CDC73 regulates the PTEN/AKT pathway through PTEN in lung adenocarcinoma cells, we conducted Western blot analysis and found that CDC73 upregulation inhibited the PTEN/AKT signaling pathway by promoting PTEN expression (Fig. 4F). In CDC73-overexpressing lung adenocarcinoma cell lines (A549OE and H1299OE), we further performed rescue experiments by knocking down PTEN.CCK-8 assays and flow cytometric cell-cycle analysis showed that following siPTEN treatment, the suppressive effect of CDC73 on cell proliferation was markedly attenuated (Fig. S4A, B, F, G). Consistently, wound-healing assays indicated a weakened inhibitory effect of CDC73 on cell migration (Fig. S4C–E). We then assessed the expression of migration-related proteins (N-cadherin, E-cadherin, SNAIL) (Fig. S4H), cell-cycle and proliferation regulators (P21, P27, Cyclin D1, CDK4) (Fig. S4I), and signaling proteins (CDC73, PTEN, p-AKT, AKT) by Western blotting (Fig. S4J). The results demonstrated that PTEN knockdown in the context of CDC73 overexpression not only diminished the inhibitory effects of CDC73 on proliferation and migration in lung adenocarcinoma cells, but also partially restored activation of the PI3K/AKT pathway. These results indicate that CDC73 inhibits the PTEN/AKT signaling pathway by regulating PTEN expression in LUAD cells, thereby contributing to the regulation of tumor progression.

### CDC73 overexpression inhibits lung adenocarcinoma cell proliferation and migration in vivo

**Fig. 5:**
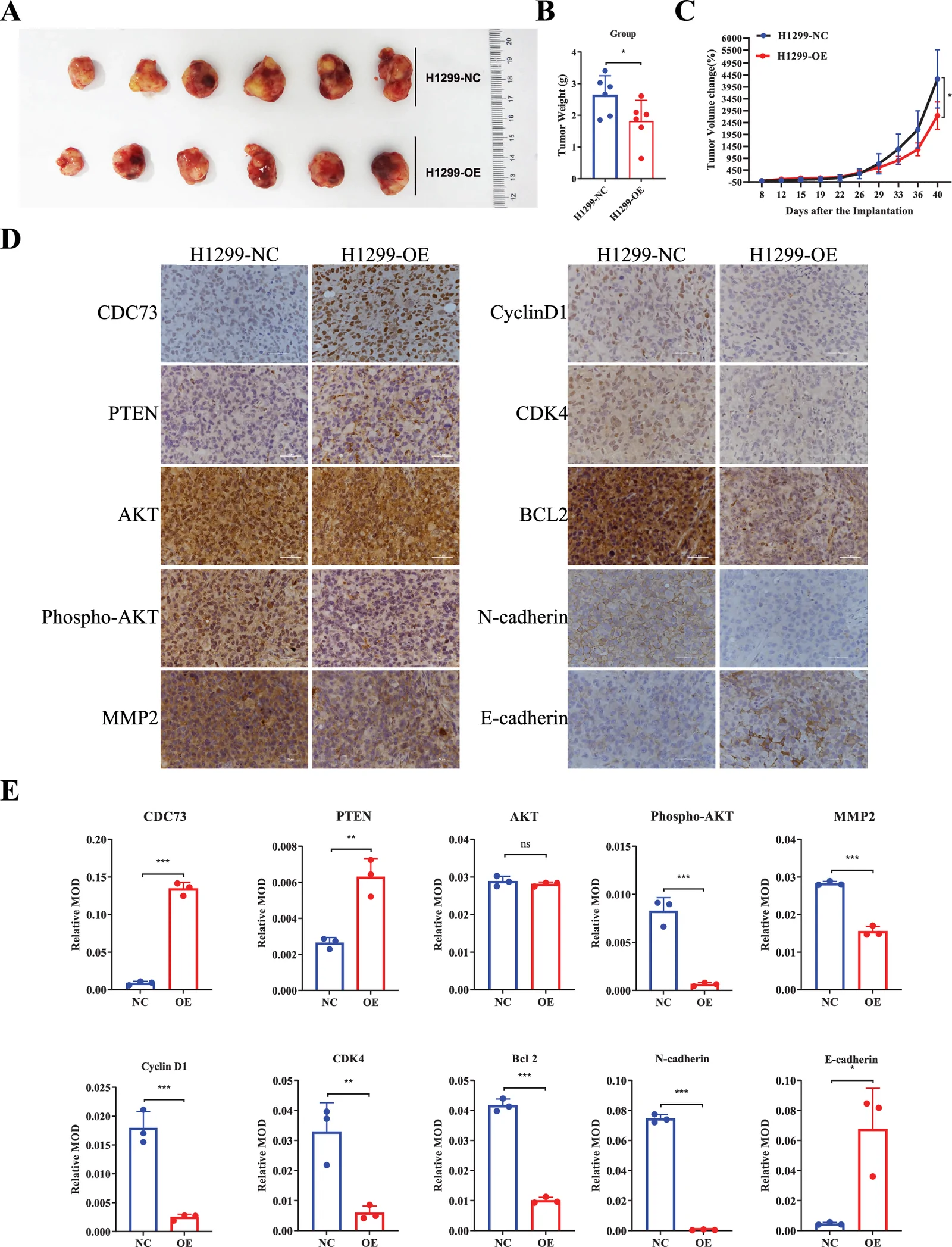
CDC73 inhibits tumor growth in vivo. **A** Tumor formation images of two groups of tumor-bearing mice (H1299-NC, H1299-OE; *n* = 6/group). **B**, **C** Changes in body weight and tumor volume in the two groups of tumor-bearing mice. **D** Immunohistochemical staining analysis of tumor tissues from both groups for CDC73, Cyclin D1, CDK4, Bcl-2, PTEN, AKT, Phospho-Akt, N-cadherin, E-cadherin, and MMP2, with statistical analysis shown in (**E**). Scale bar: 50 μm. *p < 0.05; **p < 0.01; ***p < 0.001; ns: no statistical significance.

To further confirm the role of CDC73 in lung adenocarcinoma progression, we constructed a xenograft model using H1299 cells in nude mice. The results showed that CDC73 overexpression inhibited tumor growth and weight (Fig. 5A–C). Immunohistochemical and statistical analyses of the mouse tumors revealed that the H1299-OE group showed significantly increased levels of CDC73, PTEN, and E-cadherin, while the levels of p-Akt, Cyclin D1, CDK4, BCL2, N-cadherin, and MMP2 were significantly decreased (Fig. 5D, E) (*p < 0.05; **p < 0.01; ***p < 0.001; ns: no statistical significance). These results are consistent with the findings from the in vitro experiments. In summary, our results demonstrate that CDC73 can inhibit the malignant progression of lung adenocarcinoma by regulating PTEN expression and affecting the PTEN/AKT signaling pathway.

### Characteristics and uptake of LUAD-derived extracellular vesicles

Previous studies have shown that the expression of CDC73 in plasma extracellular vesicles (EVs) from healthy donors is higher than that in early-stage lung adenocarcinoma (LUAD) patients. Additionally, increasing evidence suggests that proteins in EVs can directly act on target cells to perform specific functions [35]. Therefore, we aimed to investigate whether the levels of CDC73 in cell-derived EVs play a role in the progression of LUAD. We isolated extracellular vesicles (EVs) by differential centrifugation from human plasma and from the conditioned media of lung adenocarcinoma cells and normal bronchial epithelial cells (HBE). According to the guidelines from the Minimal Information for Studies of Extracellular Vesicles (MISEV2023), we validated the EVs using Western blot analysis for the EV marker proteins TSG101, CD63, and CD9 (Fig. S5A–C). We also examined CDC73 expression in EVs derived from A549NC,A549OE,H1299-NC and H1299-OE cells, using these marker proteins as internal references (Fig. S5B, C). The results indicated that the expression of CDC73 in EVs was consistent with that of the parent cells. Previous studies have shown that normal bronchial epithelial cells can undergo malignant transformation influenced by microenvironmental signals, simulating pre-tumor changes and spontaneous transformation [27, 28]. For example, miR-21 in EVs has been shown to promote malignant proliferation of normal HBE cells [24]. In this study, H1299 cells were stably transfected to express GFP-CD63, and EVs were subsequently isolated from the supernatants of these cells using ultracentrifugation. These EVs were co-cultured with normal HBE cells (Fig. S5F). After 24 h, confocal microscopy showed that the H1299 cell-derived EVs (green) were taken up by normal HBE cells. These results suggest that LUAD-derived EVs can be internalized by bronchial epithelial cells, and the EVs isolated from LUAD cells can be used for further experiments.

### CDC73-high extracellular vesicles secreted by LUAD cells reduce HBE cell malignant proliferation compared to the NC group

**Fig. 6:**
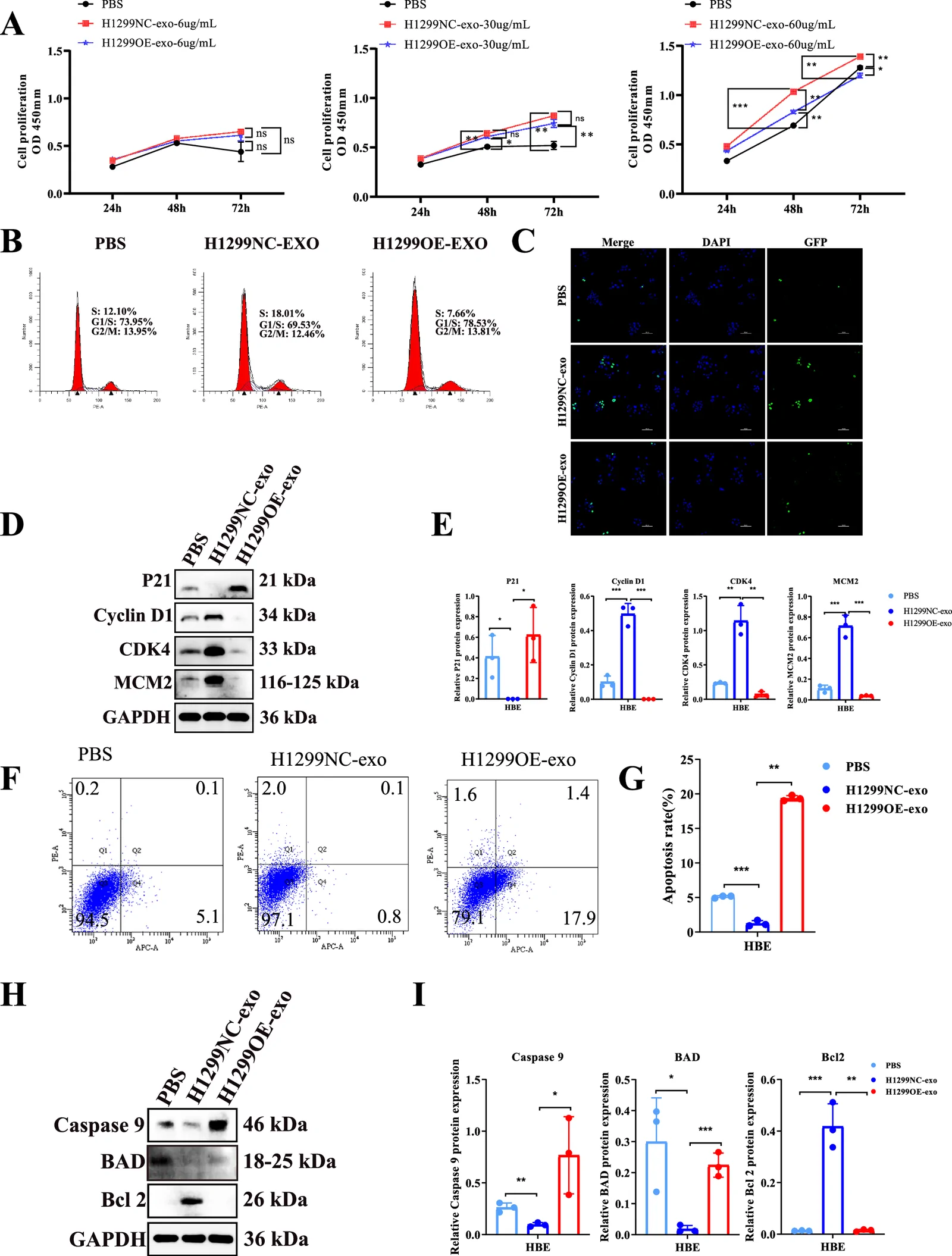
Co-culture of extracellular vesicles (EVs) from lung adenocarcinoma cells (H1299NC and H1299OE) with HBE cells reduced proliferation and enhanced apoptosis in the OE group compared with the NC group. **A** EVs from lung adenocarcinoma cells induced HBE proliferation in a dose- and time-dependent manner. **B** Cell-cycle progression of HBE cells after co-culture with H1299^NC- or H1299^OE-derived EVs, analyzed by flow cytometry. **C** EdU assay assessing HBE proliferative changes after co-culture with H1299NC- or H1299OE-derived EVs. **D**, **E** Western blot analysis and quantification of cell-cycle regulators in HBE cells after co-culture. *p < 0.05; **p < 0.01; ***p < 0.001. **F**, **G** Flow-cytometric analysis of apoptosis in HBE cells following co-culture. **H**, **I** Western blot analysis and quantification of apoptosis-related proteins in HBE cells following co-culture. *p < 0.05; **p < 0.01; ***p < 0.001.

To further explore the effect of CDC73 expression levels in LUAD-derived extracellular vesicles (EVs) on the proliferation of normal bronchial epithelial cells (HBE), we co-cultured EVs extracted from the supernatants of CDC73-overexpressing LUAD cells (H1299OE-exo) and empty vector cells (H1299NC-exo) with HBE cells for 24, 48, and 72 h. Using the CCK-8 assay, we observed that at an EV concentration of 30 μg/mL, both the H1299OE-exo and H1299NC-exo groups promoted HBE cell proliferation at 48 and 72 h compared to the PBS group, but there was no significant difference between the H1299OE-exo and H1299NC-exo groups. However, at an EV concentration of 60 μg/mL, both groups again promoted HBE cell proliferation compared to the PBS group, but the proliferation capacity of the H1299OE-exo group was significantly weaker than that of the H1299NC-exo group at 48 and 72 h. Interestingly, at 72 h, the proliferation rate of the H1299OE-exo group was even lower than that of the PBS group (Fig. 6A). These results suggest that the level of CDC73 in EVs affects HBE cell proliferation capacity (*p < 0.05; **p < 0.01; ***p < 0.001; ns: no statistical significance). Based on these results, we selected a concentration of 60 μg/mL and a co-culture time of 48 h for further experiments. Using these conditions, we performed flow cytometry to analyze the cell cycle distribution of HBE cells co-cultured with PBS, A549NC-EXO, A549OE-EXO, H1299NC-EXO, and H1299OE-EXO. The results showed that compared to the PBS group, both A549NC-EXO and H1299NC-EXO significantly increased the S phase and decreased the G0/G1 phase, indicating that LUAD-derived EVs can promote the cell cycle progression of HBE cells. Interestingly, when compared to the A549NC-EXO and H1299NC-EXO groups, the A549OE-exo and H1299OE-exo groups showed a significant reduction in the S phase and an increase in the G0/G1 phase in co-cultured HBE cells (Figs. 6B and S6A). This suggests that EVs with high CDC73 expression reduce the ability of LUAD-derived EVs to promote HBE cell cycle progression compared to the NC group. Subsequently, we performed an EdU assay to analyze the proliferation ability of HBE cells co-cultured with PBS, A549NC-EXO, A549OE-EXO, H1299NC-EXO, and H1299OE-EXO. The results showed that, compared to the PBS group, the proliferation ability (green fluorescence) of HBE cells was enhanced in both the A549NC-EXO and H1299NC-EXO groups (Figs. 6C and S6B). However, when compared to the A549NC-EXO and H1299NC-EXO groups, the proliferation ability of HBE cells co-cultured with A549OE-exo and H1299OE-exo was reduced. These results indicate that CDC73-high EVs reduce the ability of LUAD-derived EVs to promote HBE cell proliferation compared to the NC group. Finally, we analyzed the expression of cell cycle and proliferation-related proteins in HBE cells co-cultured with PBS, A549NC-EXO, A549OE-EXO, H1299NC-EXO, and H1299OE-EXO using Western blot. The results showed that, compared to the PBS group, both the A549NC-EXO and H1299NC-EXO groups exhibited reduced expression of p21, with increased expression of CyclinD1, CDK4, and MCM2. However, compared to the A549NC-EXO and H1299NC-EXO groups, the A549OE-exo and H1299OE-exo groups exhibited increased expression of p21 and decreased expression of CyclinD1, CDK4, and MCM2 (Figs. 6D, E and S6C, D) (*p < 0.05; **p < 0.01; ***p < 0.001).Using flow cytometry, we analyzed the apoptosis of HBE cells co-cultured with PBS, A549NC-EXO, A549OE-EXO, H1299NC-EXO, and H1299OE-EXO. The results showed that, compared to the PBS group, both the A549NC-EXO and H1299NC-EXO groups exhibited reduced apoptosis. This suggests that LUAD-derived EVs have the ability to inhibit apoptosis in HBE cells. Interestingly, compared to the A549NC-EXO and H1299NC-EXO groups, the A549OE-exo and H1299OE-exo groups showed an increase in apoptosis in co-cultured HBE cells (Figs. 6F, G and S6E) (**p < 0.01; ***p < 0.001). These results indicate that EVs with high CDC73 expression have a weakened ability to inhibit HBE cell apoptosis compared to the NC group. We further analyzed apoptosis-related proteins in HBE cells co-cultured with PBS, A549NC-EXO, A549OE-EXO, H1299NC-EXO, and H1299OE-EXO using Western blot. The results showed that, compared to the PBS group, the A549NC-EXO and H1299NC-EXO groups exhibited reduced expression of Caspase9 and BAD, while Bcl2 expression increased. However, in comparison with the A549NC-EXO and H1299NC-EXO groups, the A549OE-exo and H1299OE-exo groups showed increased expression of Caspase9 and BAD and decreased expression of Bcl2 in co-cultured HBE cells (Figs. 6H, I and S6F) (*p < 0.05; **p < 0.01; ***p < 0.001). In conclusion, LUAD-derived extracellular vesicles (EVs) drive cell-cycle progression, enhance proliferation, and suppress apoptosis in HBE cells. Notably, when CDC73 is highly expressed in these EVs, these pro-proliferative and anti-apoptotic effects are attenuated, resulting in reduced cell-cycle advancement, diminished malignant proliferation, and a weaker inhibition of apoptosis compared with the NC group.

### CDC73-high extracellular vesicles secreted by LUAD cells show a weakened ability to promote HBE cell migration compared to the NC group

**Fig. 7:**
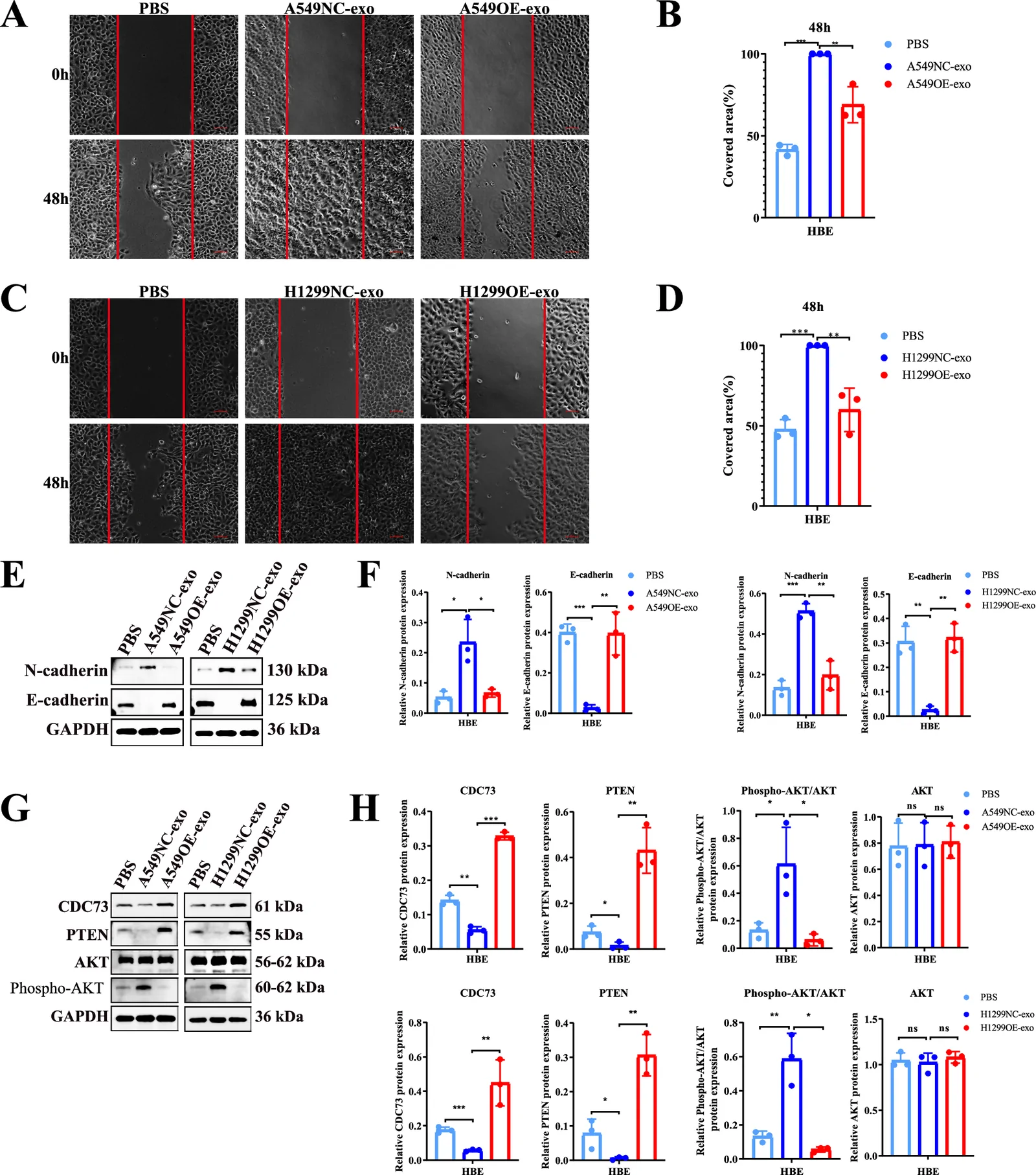
Following co-culture of HBE cells with extracellular vesicles (EVs) from lung adenocarcinoma cells (A549NC, A549OE, H1299NC, H1299OE), HBE cell migration was reduced in the OE groups compared with the NC groups. CDC73 enriched in EVs from overexpressing cells modulated PTEN expression and thereby suppressed the PTEN/AKT signaling pathway. **A**–**D** Wound-healing assays assessing migration of HBE cells after co-culture with EVs from A549NC/A549OE and H1299NC/H1299OE cells. **E**, **F** Western blot analysis and quantification of migration-related proteins in HBE cells after co-culture. **G**, **H** Western blot analysis and quantification of proteins in the PTEN/AKT pathway following co-culture. *p < 0.05; **p < 0.01; ***p < 0.001; ns, not significant.

We continued our study under the experimental conditions of co-culturing HBE cells with LUAD-derived extracellular vesicles (EVs) at a concentration of 60 μg/mL for 48 h. Using a wound healing assay, we analyzed the migration ability of HBE cells co-cultured with PBS, A549NC-EXO, A549OE-EXO, H1299NC-EXO, and H1299OE-EXO. The results showed that, compared to the PBS group, both A549NC-EXO and H1299NC-EXO groups enhanced the migration ability of HBE cells, suggesting that LUAD-derived EVs promote HBE cell migration. Interestingly, when compared to the A549NC-EXO and H1299NC-EXO groups, the migration ability of HBE cells co-cultured with A549OE-exo and H1299OE-exo was significantly reduced (Fig. 7A–D) (**p < 0.01; ***p < 0.001). These results indicate that EVs with high CDC73 expression reduce the ability to promote HBE cell migration compared to the NC group. We further analyzed epithelial-mesenchymal transition (EMT)-related proteins in HBE cells co-cultured with PBS, A549NC-EXO, A549OE-EXO, H1299NC-EXO, and H1299OE-EXO using Western blot. The results showed that, compared to the PBS group, both A549NC-EXO and H1299NC-EXO groups exhibited increased expression of N-cadherin and decreased expression of E-cadherin. However, compared to the A549NC-EXO and H1299NC-EXO groups, the A549OE-exo and H1299OE-exo groups showed reduced N-cadherin expression and increased E-cadherin expression in co-cultured HBE cells (Fig. 7E, F) (*p < 0.05; **p < 0.01; ***p < 0.001). Additionally, we analyzed proteins related to the PTEN/AKT pathway in HBE cells co-cultured with PBS, A549NC-EXO, A549OE-EXO, H1299NC-EXO, and H1299OE-EXO. The results showed that, compared to the PBS group, both the A549NC-EXO and H1299NC-EXO groups exhibited reduced CDC73 and PTEN expression, with increased p-AKT expression. However, in comparison with the A549NC-EXO and H1299NC-EXO groups, the A549OE-exo and H1299OE-exo groups showed increased CDC73 and PTEN expression and decreased p-AKT expression in co-cultured HBE cells (Fig. 7G, H) (*p < 0.05; **p < 0.01; ***p < 0.001; ns: no statistical significance). In conclusion, our study found that LUAD-derived EVs have the ability to promote HBE cell migration. Interestingly, EVs with high CDC73 expression reduce the ability to promote HBE cell migration compared to the NC group. Furthermore, we found that EVs from the NC group promoted the PTEN/AKT pathway, while EVs with high CDC73 expression inhibited the PTEN/AKT pathway by upregulating PTEN expression.

### CDC73 within extracellular vesicles secreted by HBE cells suppresses the malignant progression of lung adenocarcinoma

**Fig. 8:**
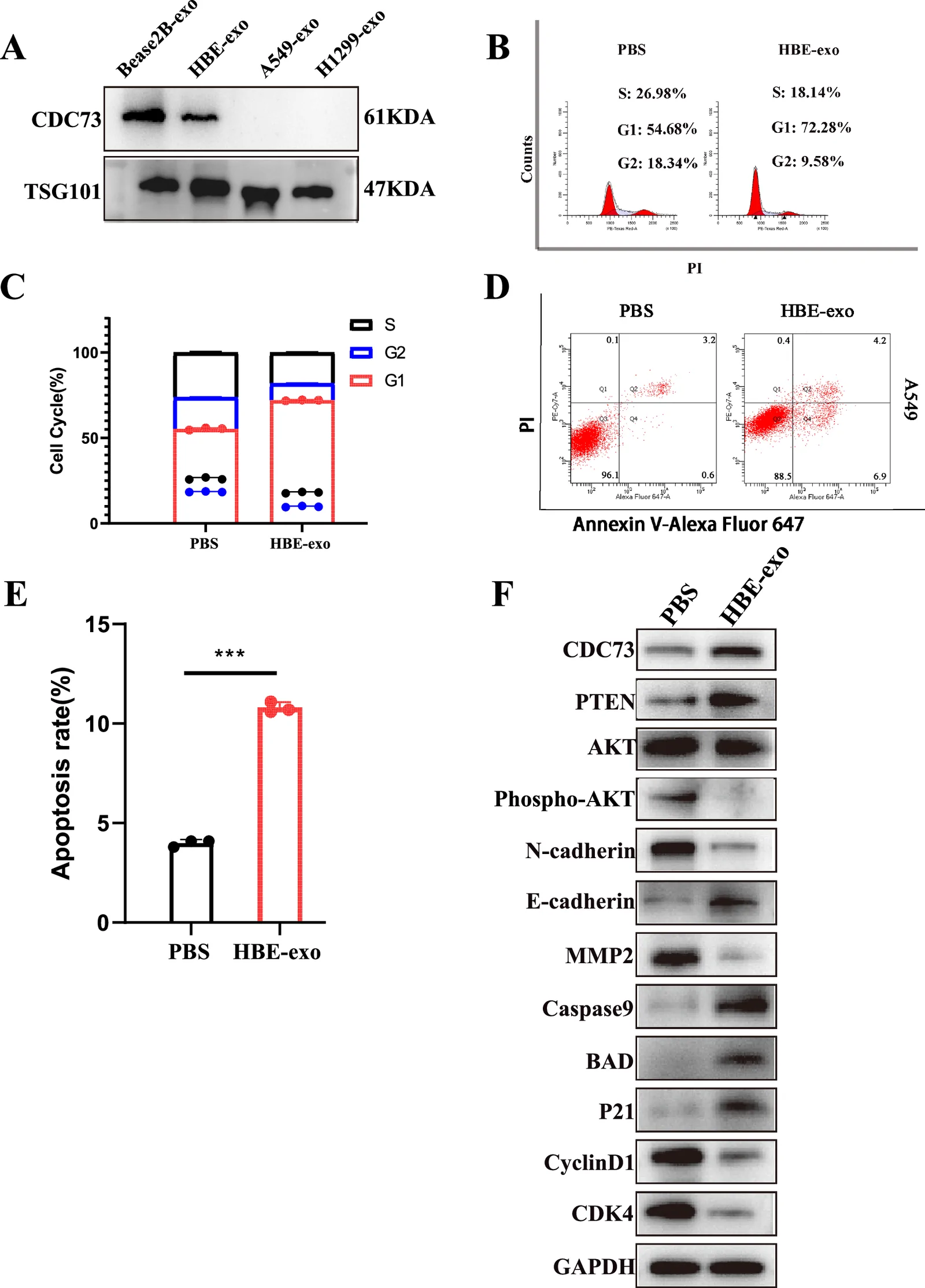
Co-culture of A549 cells with extracellular vesicles from normal pulmonary epithelial HBE cells (HBE-exo) versus PBS control showed reduced proliferation and increased apoptosis in the HBE-exo group. **A** Western blot analysis comparing CDC73 abundance in EVs secreted by normal pulmonary epithelial cells versus lung adenocarcinoma cells. **B**, **C** Flow cytometric analysis of cell-cycle distribution in A549 cells following HBE-exo treatment, with quantitative summary. **D**, **E** Flow cytometric assessment of apoptosis in A549 cells after HBE-exo treatment, with statistical analysis. ***p < 0.001. **F** Western blot analysis of proliferation-, apoptosis-, and PTEN pathway–related proteins in A549 cells co-cultured with HBE-derived EVs versus PBS control.

We first compared CDC73 expression in EVs secreted by normal pulmonary epithelial cells (Beas-2B, HBE) and lung adenocarcinoma cells (A549, H1299) by Western blotting. CDC73 levels were markedly higher in EVs derived from normal epithelial cells than in those from lung adenocarcinoma cells (Fig. 8A). We then co-cultured A549 cells with EVs released by HBE cells for 48 h. Flow cytometry showed a pronounced G1 arrest: the proportion of cells in G1 increased from 54.68% in the PBS control to 72.28% in the HBE-exo group (Fig. 8B, C). Apoptosis assays further indicated that the apoptotic rate rose from 3.8% in controls to 11.1% after HBE-exo treatment (Fig. 8D, E). Mechanistically, Western blotting revealed alterations in cell-cycle regulators (P21, Cyclin D1, CDK4), migration-related proteins (N-cadherin, E-cadherin, MMP2), apoptosis-related proteins (Caspase-9, BAD), and components of the PTEN/AKT pathway (PTEN, p-AKT, AKT). HBE-exo treatment markedly upregulated CDC73, concomitantly suppressing proliferation and migration, promoting apoptosis, and attenuating activation of the PTEN/AKT signaling axis (Fig. 8F). Together, these findings demonstrate that CDC73 is expressed at substantially higher levels in EVs from normal pulmonary epithelial cells than in those from lung adenocarcinoma cells. EVs secreted by normal epithelial cells can upregulate CDC73 and thereby restrain the malignant progression of lung adenocarcinoma cells.

## Discussion

Lung adenocarcinoma (LUAD) is the most common subtype of lung cancer, accounting for 50% of all lung cancer diagnoses, and its incidence is on the rise [36]. Although previous studies have reported that CDC73 is an important tumor suppressor gene [4, 9], its biological function in LUAD has not been clearly defined. In this study, our findings demonstrate that CDC73 plays a critical role in regulating LUAD progression. We constructed LUAD cell lines that overexpress CDC73 using lentiviral plasmids. Through cell proliferation assays, Transwell assays, wound healing assays, and flow cytometry, we confirmed that CDC73 is involved in LUAD cell proliferation, invasion, migration, and cell cycle apoptosis. Mechanistically, we found that CDC73 exerts its biological function by regulating PTEN. Upregulation of CDC73 led to an increase in PTEN expression, which subsequently inhibited the classical PTEN/AKT pathway, a pathway known to be associated with cancer growth and metastasis. Flow cytometry analysis showed that CDC73 overexpression in LUAD cells induced G1/S arrest. When investigating the regulatory mechanisms of CDC73 on the cell cycle, we found that the expression of G1/S-related proteins P21 and P27 was upregulated, while Cyclin D1 and CDK4 expression was downregulated. Previous studies have shown that CDK4 and Cyclin D1 form a complex that promotes the G1 to S phase transition, and their reduced expression can inhibit this progression [37]. Tumor suppressor genes typically negatively regulate processes related to cell cycle progression, preventing uncontrolled cell growth [38, 39]. Our research also indicates that the tumor suppressor gene CDC73 may inhibit LUAD malignancy by regulating the cell cycle. The malignant transformation of an initially benign tumor is driven by changes that enable sustained tumor cell survival, making the induction of apoptosis a cornerstone of anticancer therapies. Apoptosis can occur via intrinsic or extrinsic pathways, with the intrinsic pathway being triggered by the release of cytochrome C into the cytoplasm, leading to caspase-9 activation [40]. Our flow cytometry analysis revealed that CDC73 overexpression stimulates apoptosis in A549 and H1299 cells. We found that overexpression of CDC73 upregulated the pro-apoptotic proteins BAD and caspase-9, while downregulating the anti-apoptotic protein Bcl-2.

The transformation of tumor cells into mesenchymal-like cells is dependent on the induction of epithelial-mesenchymal transition (EMT) [41]. In the case of lung adenocarcinoma (LUAD), the presence of metastatic disease typically indicates poor prognosis and shorter overall survival. SNAIL1, a transcription factor, plays a crucial regulatory role in the induction of EMT and its related malignant mechanisms. It has been proposed that SNAIL1 promotes EMT by upregulating N-cadherin transcription while downregulating E-cadherin [42, 43]. Additionally, E-cadherin disrupts cell-cell adhesion and limits the secretion of MMP proteins [44]. Our study found that CDC73 overexpression significantly alters the migration and invasion capabilities of LUAD cells as well as the expression of EMT-related genes (SNAIL1, N-cadherin, MMP-2, and E-cadherin), suggesting that CDC73 negatively regulates LUAD cell migration. Multiple studies have shown that the PTEN/AKT signaling pathway plays an important role in tumor progression [45]. Zheng et al. conducted transcriptome sequencing and bioinformatic analysis of colorectal cancer, revealing a strong correlation between CDC73 and the PTEN/AKT signaling pathway in colorectal cancer cells [10], although the exact mechanism of this inhibition has yet to be explored. PTEN, a negative regulator of the PI3K-AKT pathway, reduces AKT phosphorylation and inhibits pathway activation when activated [26]. As a second messenger, PIP3 is produced and activated by PI3K [46]. In this context, CDC73 upregulation leads to increased PTEN expression, which in turn catalyzes the dephosphorylation of PIP3 to PIP2, thereby blocking the activation of the PI3K-AKT pathway [47]. These findings strongly suggest that PTEN may mediate the regulatory effects of CDC73 on the PTEN/AKT pathway. To confirm the interaction between CDC73 and PTEN in LUAD, we performed co-immunoprecipitation (CO-IP), mass spectrometry analysis, and rescue experiments, which revealed for the first time that the tumor suppressor genes CDC73 and PTEN interact in LUAD cells. Based on qualitative proteomics, we conducted protein interaction network analysis, which showed that CDC73 upregulates PTEN expression to inhibit the PTEN/AKT pathway, thus exerting a functional role in inhibiting LUAD growth.

Although extensive studies have been conducted on the role of miRNAs in extracellular vesicles (EVs) in lung cancer diagnosis, treatment, and pathogenesis, research on proteins in EVs is relatively scarce [35, 47–49]. Previous studies have shown that protein profiling of extracellular vesicles can aid in the prediction and diagnosis of cholangiocarcinoma [50]; however, research leveraging EV-associated proteins to predict and diagnose lung adenocarcinoma remains limited. In this study, we found that CDC73 expression in plasma EVs from LUAD patients was significantly lower than that in healthy donors. Based on sequencing results, we also conducted small-scale clinical validation. Our study suggests that CDC73 in EVs could serve as a potential non-invasive diagnostic biomarker for LUAD. Previous studies have reported that tumor-derived EVs can promote the malignant transformation of normal cells [24, 51]. Our results indicate that LUAD-derived EVs promote HBE cell proliferation and migration while inhibiting apoptosis. Interestingly, the ability of CDC73-high EVs secreted by LUAD cells to promote HBE cell proliferation and migration and inhibit apoptosis was reduced compared to the NC group. EVs derived from HBE cells suppressed A549 cell proliferation and migration while promoting apoptosis. Furthermore, we found that CDC73 in EVs upregulates PTEN expression, thereby inhibiting the PTEN/AKT pathway.

Nevertheless, several limitations of the present study should be acknowledged. First, the clinical validation cohort was relatively small, and larger independent cohorts will be required to further establish the diagnostic value of EV-borne CDC73. Second, although the current study was designed to focus on the biological effects of CDC73 restoration and CDC73-enriched EVs in the context of LUAD-associated CDC73 downregulation, complementary CDC73 loss-of-function models, including stable knockdown or genetic knockout systems, were not included. Third, while PTEN knockdown-based rescue experiments supported the functional involvement of the PTEN/AKT axis, additional in vivo pharmacologic or genetic rescue studies would further strengthen the mechanistic conclusions. These issues warrant further investigation in future studies.

## Conclusions

In summary, our study demonstrates that CDC73 inhibits the malignant progression of LUAD by regulating PTEN expression and suppressing the PTEN/AKT signaling pathway. High CDC73 expression in EVs inhibits the ability of LUAD cells to promote the malignant transformation of HBE cells, and it similarly regulates PTEN expression to suppress the PTEN/AKT pathway. Therefore, we propose that CDC73 in EVs could serve as a potential target and biomarker for the clinical diagnosis and treatment of LUAD patients.

## Materials and methods

### Clinical plasma samples

Plasma samples were collected from 11 healthy donors and 14 early-stage lung adenocarcinoma patients. All early-stage lung adenocarcinoma patients underwent surgery and were confirmed by histopathology. None of the patients received radiotherapy or chemotherapy prior to blood collection. The study was approved by the Ethics Committees of the Second Affiliated Hospital of Nantong University and the Jinling Hospital Affiliated with Nanjing University, with informed consent obtained from all participants before enrollment. This study adheres to the principles of the Declaration of Helsinki.

### IBT quantification proteomics

Based on clinical pathology results, venous blood samples were collected from untreated typical LUAD patients and healthy controls. Extracellular vesicles were isolated from plasma using ultracentrifugation. Quantitative proteomics analysis using isobaric labeling technology (IBT) was performed by BGI Genomics (Beijing). In a single experiment, a log2FC absolute value > 1 and a *p* value < 0.05 under two screening conditions were considered significant. Raw mass spectrometry data were obtained and compared using protein databases with specialized software.

### Co-immunoprecipitation

HBE and H1299 cells (9 × 10⁶ cells) were lysed in RIPA lysis buffer supplemented with 1% protease inhibitor cocktail for 10 min. The lysates were then centrifuged at 12,000 × *g* at 4 °C for 10 min, and the supernatants were collected for further experimentation. The collected supernatants were incubated overnight with the indicated antibodies, followed by incubation with agarose magnetic beads (10 μL) pre-washed three times with RIPA lysis buffer. The mixture was shaken overnight at 4 °C to capture the immune complexes. Afterward, the samples were centrifuged at 12,000 × *g* for 1 min, the supernatants were discarded, and the pellets were washed three times with RIPA lysis buffer. The samples were then eluted by boiling in 2× Protein Sample Loading Buffer for 5 min and analyzed by SDS-polyacrylamide gel electrophoresis (SDS-PAGE) and immunoblotting. Mass spectrometry-based proteomics was subsequently performed by Hanno Biotechnology (Suzhou) Co., Ltd. (HN2022_GP_1114).

### Preparation and characterization of extracellular vesicles (EVs)

Briefly, plasma or cell culture supernatants were centrifuged at 1000 × *g* and 10,000 × *g* for 10 min each to remove debris. The supernatants were then filtered through a 0.22 μm filter to eliminate impurities, and the pre-cleared supernatants were ultracentrifuged at 100,000 × *g* for 1 h to pellet the extracellular vesicles. The supernatants were discarded, and the EV pellet was washed with PBS, followed by another round of ultracentrifugation at 100,000 × *g* for 1 h. The final EV pellet was resuspended in balanced salt solution for subsequent experiments. Transmission electron microscopy (TEM) and nanoparticle tracking analysis (NTA) were used to characterize the ultrastructure and size of the EVs.

### Uptake of CD63-labeled extracellular vesicles by HBE cells

A stable H1299 cell line expressing GFP-CD63 was constructed through monoclonal selection. GFP-CD63-labeled extracellular vesicles were extracted from the supernatants of the stable GFP-CD63-expressing H1299 cells by ultracentrifugation and co-cultured with HBE cells for 12–24 h. F-actin was stained using CL594-Phalloidin, and the nuclei were stained with DAPI. Finally, the distribution of GFP-CD63 fluorescence within the cells was examined using confocal microscopy.

### Immunohistochemistry

Immunohistochemistry was performed on xenograft tumor sections. Briefly, the sections were dewaxed and treated with 0.01 M sodium citrate buffer using a steam antigen retrieval method for 4 min. The sections were then blocked with 5% BSA at room temperature for 20 min to prevent non-specific staining and inhibit endogenous peroxidase activity. Following this, the sections were incubated overnight in humidified boxes with primary antibodies (CDK73, Cyclin D1, CDK4, Bcl-2, PTEN, Akt, N-cadherin, E-cadherin, and MMP2, all from Proteintech, China). The next day, the sections were incubated at room temperature for 2 h with an enzyme-conjugated secondary antibody polymer. Protein expression was visualized in situ using DAB chromogenic substrates, and hematoxylin and eosin (HE) staining was also performed. The slides were examined under an optical microscope by two independent pathologists, who scored the immunostaining for each specimen. Staining intensity was categorized into four levels: 3 for strong staining, 2 for moderate staining, 1 for weak staining, and 0 for no staining. The final staining score was calculated based on the percentage of positive cells and their respective intensity scores.

### Cell culture and transfection

Non-small cell lung cancer (NSCLC) cell lines (A549, H1299, PC9, 95D, H460) and normal cells (HBE, Bease-2B) were obtained from the Cell Bank of the Chinese Academy of Sciences. NSCLC cells were cultured in DMEM or RPMI-1640 supplemented with 10% fetal bovine serum. Unless otherwise specified, all cells were maintained at 37 °C in a humidified incubator with 5% CO₂. A549 and H1299 cells at 60–80% confluence were transduced with lentiviral particles from GeneChem and, following clonal selection, stable CDC73-overexpressing lines (A549OE and H1299OE) were established. After transduction, cells were washed with PBS to remove residual complete medium and lysed for protein extraction. Transduction efficiency was assessed by Western blotting. Subsequently, PTEN-targeting siRNA (MCE: HY-RS11363) was transfected into A549OE and H1299OE cells to knock down PTEN expression, and transfection efficiency was evaluated by Western blotting.

### Monoclonal experiment

Cells at the logarithmic growth stage were transfected, trypsin-digested, and collected by centrifugation. After being washed with PBS, complete medium was added, and cell counting was performed. The cell suspension was then diluted by gradient multiple times until one cell was placed in 10 μl of medium. After that, a 96-well plate was inoculated with single cells and cultured in a cell incubator with a 5% CO_2_ and 37 °C constant temperature humidified atmosphere for 2–3 weeks. Cells without fluorescence expression were removed, while cells with stable fluorescence expression were further cultured. Finally, Western immunoblotting was employed to determine transfection efficiency.

### Cell Counting Kit-8

Cells were seeded in 96-well plates at a density of 3000 cells per well in 100 µL of complete culture medium and incubated for 24, 48, and 72 h, respectively. After incubation, 10 µL of CCK-8 solution was added to each well, and the plates were incubated for an additional 2–4 h before measuring the absorbance. The experiment was terminated, and the optical density (OD) at 450 nm was measured using a multifunctional microplate reader. Each experiment was repeated at least three times.

### EDU image analysis

Cells were seeded at 4000 cells per well and incubated for 12 h. Then, the Cell-Light™ EDU Apollo643 In Vitro Kit was used to assess cell proliferation according to the manufacturer’s instructions, analyzing the DNA replication status of the cells.

### Flow cytometry analysis

To assess cell cycle distribution, cells were cultured until they reached 70–80% confluency. The cells were washed with PBS, digested with trypsin, and centrifuged at 1200 × *g* for 6 min. After removing the supernatant, the cells were washed twice with PBS and fixed in 10 mL of 75–80% ethanol, then stored at −20 °C for at least 12 h. After fixation, the cells were washed twice with PBS, and the supernatant was discarded. The cell pellet was resuspended in PI/RNase staining solution and incubated for 15 min at room temperature in the dark. PI signals were analyzed using a BD LSRFortessa instrument to determine cell cycle distribution, and comparisons were made. To assess apoptosis, cells were collected in centrifuge tubes, washed once with PBS, and counted. Cells were resuspended at a concentration of 2 × 10⁶ cells/mL. Annexin V-Alexa Fluor647/PI apoptosis detection kit was used according to the manufacturer’s instructions. After incubating the cells on ice in the dark for 15 min, they were immediately analyzed using the BD LSRFortessa instrument. Each experiment was repeated at least three times.

### Wound healing assay

Cells were seeded in 6-well plates at a density of 1.0 × 10⁶ cells per well and incubated overnight. Once the cells reached full confluency, a 10 μL pipette tip was used to manually scratch the cell monolayer. The cells were then incubated in serum-free medium for 24 and 48 h, and images were taken at each time point. Each experiment was repeated three times. The wound area was quantified using Image Pro Plus software.

### Transwell invasion assay

Matrigel matrix gel was added to the upper chamber of the Transwell insert. After allowing the gel to solidify for 5 h, any excess liquid was removed from the top of the gel. The lower chamber was filled with medium containing 20% serum, and cells were diluted in serum-free medium (2 × 10⁴/300 μL) before being seeded on top of the gel in the upper chamber. The Transwell plate was incubated in a humidified atmosphere at 37 °C with 5% CO2 for 12–36 h. Cells that invaded through the gel and reached the lower membrane surface were fixed with 4% paraformaldehyde, stained, and photographed. The invaded cells were quantified using Image Pro Plus software.

### Immunofluorescence staining (IF)

Cells were cultured on glass coverslips 1 day prior to the experiment. After removing the residual culture medium with PBS, the cells were fixed with 4% paraformaldehyde for 5 min. The cells were then permeabilized with 0.1% Tween-20 solution for 30 min, followed by blocking with 3% BSA buffer for 1 h. Subsequently, the cells were incubated overnight on ice with a primary antibody mixture containing the target antibody. After washing off the primary antibody with PBS, the cells were incubated with a rabbit secondary antibody for 2 h. Finally, the nuclei were stained with DAPI for 5 min, and images were immediately captured using a confocal microscope.

### Western immunoblotting assay

After extracting cellular proteins, total protein concentration was assessed using the BCA protein assay kit (Beyotime, China), and a standard curve was generated. Total proteins were separated by 10% SDS-polyacrylamide gel electrophoresis (SDS-PAGE) (Cat No. PG120, PG121; Epizyme, Shanghai, China) and transferred to PVDF membranes. Alternatively, silver staining was performed using a quick silver staining kit (P0017S, Beyotime). Each experiment was independently repeated at least three times. The PVDF membranes were blocked with 5% skim milk diluted in TBST for 1–2 h, followed by washing with TBST for 5 min, repeated three times. The membranes were then incubated overnight at 4 °C with primary antibodies at dilutions specified in the respective antibody datasheets. The antibodies used included: CDC73 (Proteintech, USA), P21 (Proteintech, USA), P27 (Proteintech, USA), Cyclin D1 (Proteintech, USA), MCM2 (Proteintech, USA), CDK4 (CST, USA), Caspase9 (CST, USA), BAD (Proteintech, USA), Bcl-2 (Proteintech, USA), PTEN (Proteintech, USA), Akt (Proteintech, USA), Phospho-Akt (Proteintech, USA), SNAIL1 (Proteintech, USA), N-cadherin (Proteintech, USA), E-cadherin (Proteintech, USA), GAPDH (Proteintech, USA), Beta-actin (Proteintech, USA), CD63 (Abcam, UK), CD9 (Abcam, UK), TSG101 (Abcam, UK), and MMP2 (Proteintech, China). The following day, the membranes were incubated with secondary antibodies at room temperature for 1 h. Protein bands were visualized using the ECL detection system (Thermo Fisher Scientific).

### Xenograft model

A total of 1–2 × 10⁶ cells were injected subcutaneously into the right forelimb axilla of 6–8 week-old nude mice using a 1 mL syringe. The size, weight, and volume of all tumors were recorded. Once the average tumor size reached 1500–2000 mm³, the mice were sacrificed for histopathological evaluation. All animal care and laboratory procedures were conducted in accordance with approval from the Ethics Committee of the First People’s Hospital of Nantong.

### Statistical analysis

Graphs and statistical data were generated using GraphPad Prism 8 software. Differences between the experimental and control groups were analyzed using the t-test. Each experiment was repeated at least three times, and a *p* value of <0.05 was considered statistically significant.

## Supplementary information

Supplementary legends

original flow cytometry data for apoptosis analysis.

SFigure1

SFigure2

SFigure3

SFigure4

SFigure5

SFigure6

Full and uncropped western blots

## Data Availability

The datasets used and/or analyzed during the current study are available from the corresponding author upon reasonable request. The quantitative proteomics sequencing data can be accessed via ProteomeXchange with the identifier PXD042463.
