# Supplementary material for: CDC73 suppresses lung adenocarcinoma via the PTEN/AKT pathway and exhibits biomarker and functional potential in extracellular vesicles
Source: Cell Death Discov. 2026 Aug 14;12:412. doi: 10.1038/s41420-026-03305-6 (PMC13652814; doi:10.1038/s41420-026-03305-6)
Supplement: Supplementary file 1 [file 41420_2026_3305_MOESM2_ESM.pdf]

A549 OE

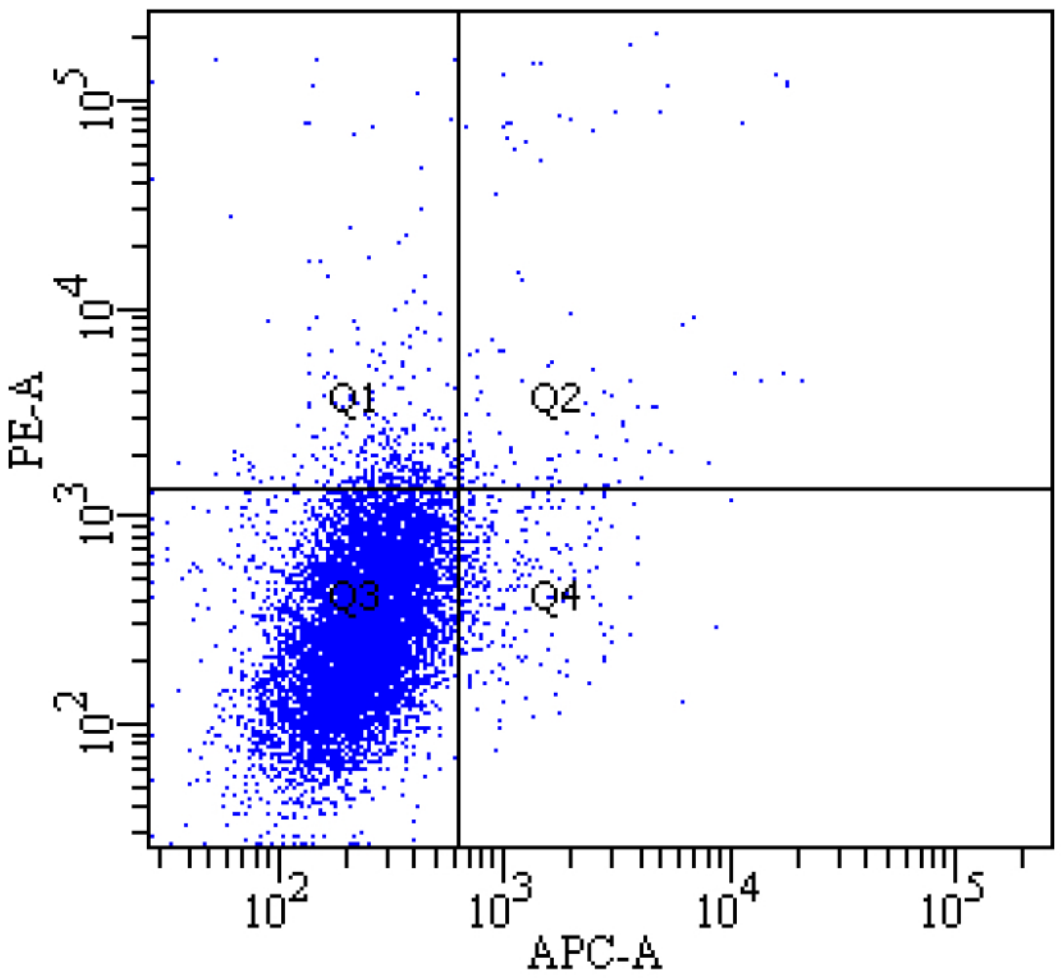

Tube: OX-1

Population	#Events	%Parent	%Total
All Events	17,402	####	100.0
P1	10,000	57.5	57.5
P2	9,014	90.1	51.8
Q1	339	3.8	1.9
Q2	112	1.2	0.6
Q3	8,282	91.9	47.6
Q4	281	3.1	1.6

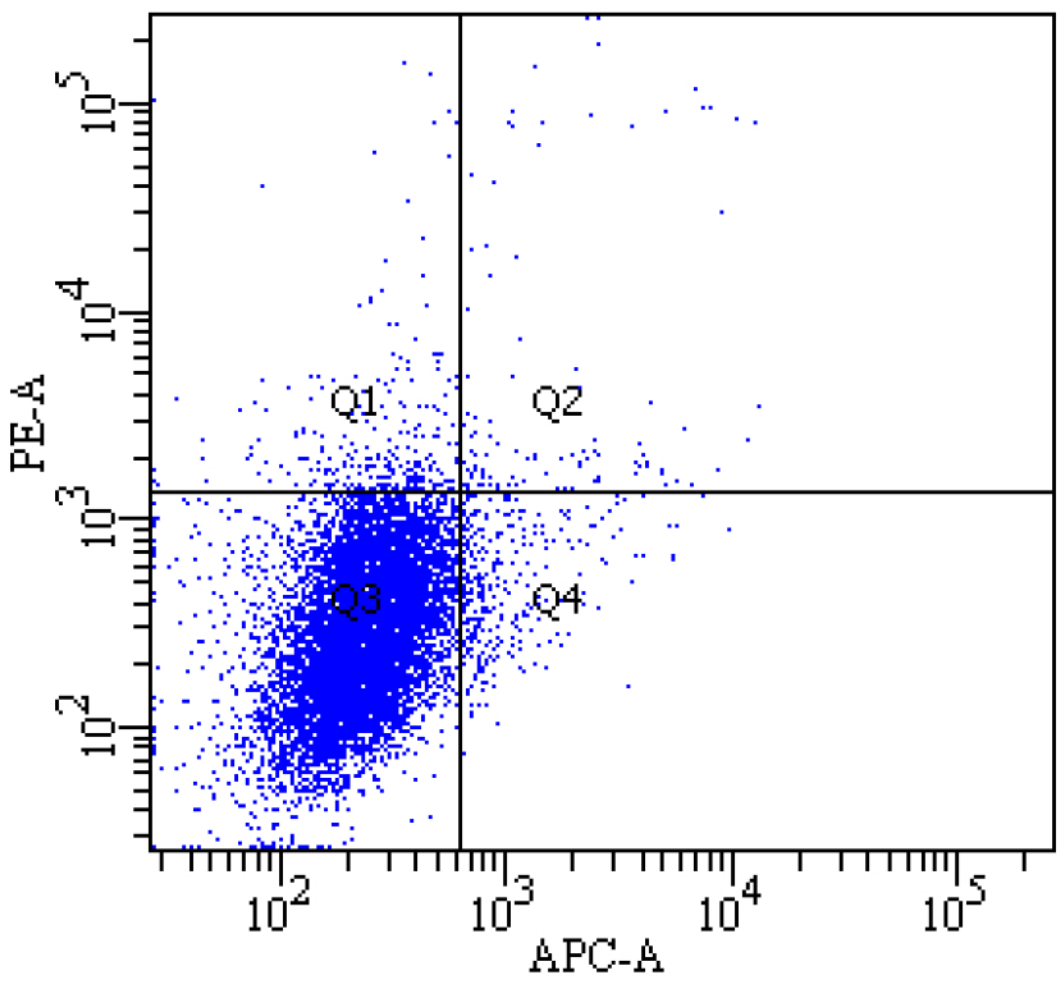

Tube: OX-2

Population	#Events	%Parent	%Total
All Events	18,676	####	100.0
P1	10,000	53.5	53.5
P2	8,980	89.8	48.1
Q1	269	3.0	1.4
Q2	103	1.1	0.6
Q3	8,281	92.2	44.3
Q4	327	3.6	1.8

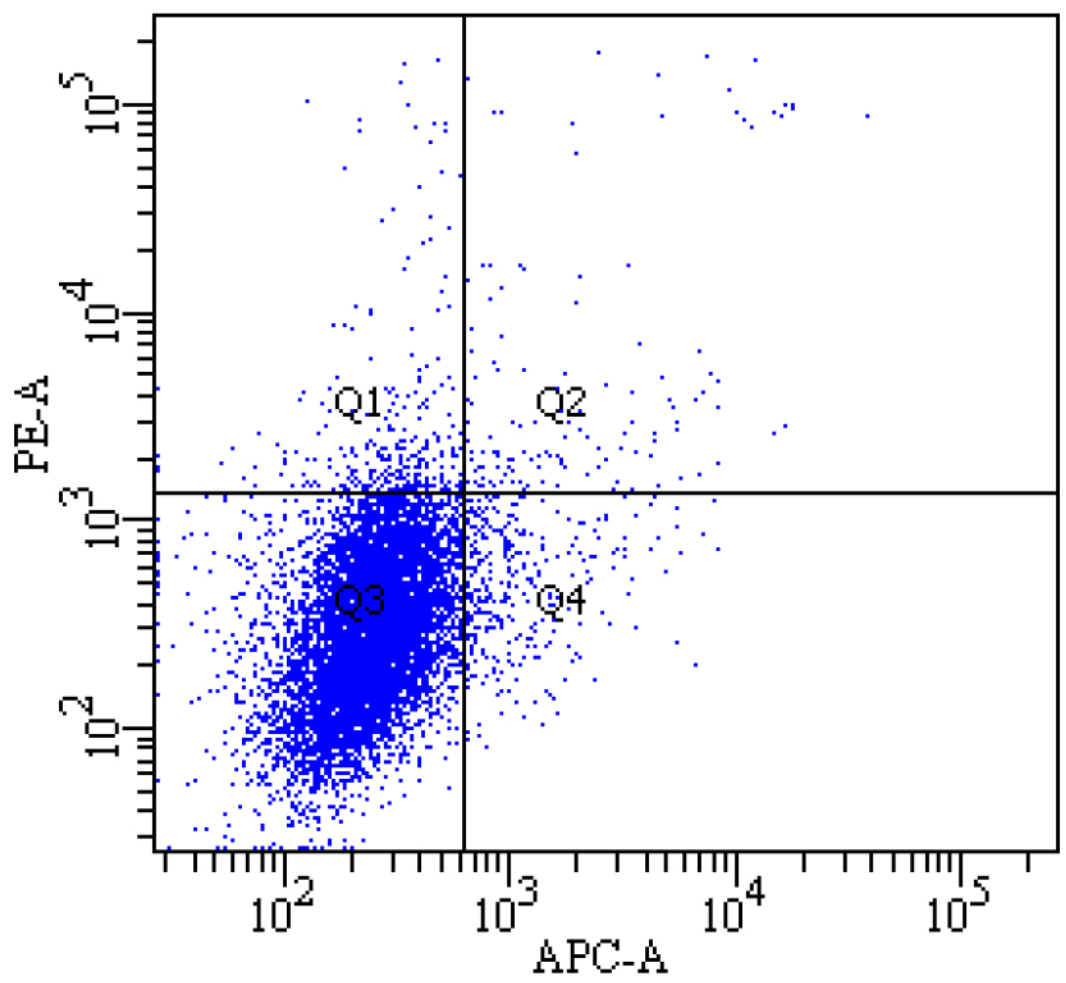

Tube: OX-3

Population	#Events	%Parent	%Total
All Events	18,322	####	100.0
P1	10,000	54.6	54.6
P2	8,997	90.0	49.1
Q1	312	3.5	1.7
Q2	127	1.4	0.7
Q3	8,227	91.4	44.9
Q4	331	3.7	1.8

A549 PBS

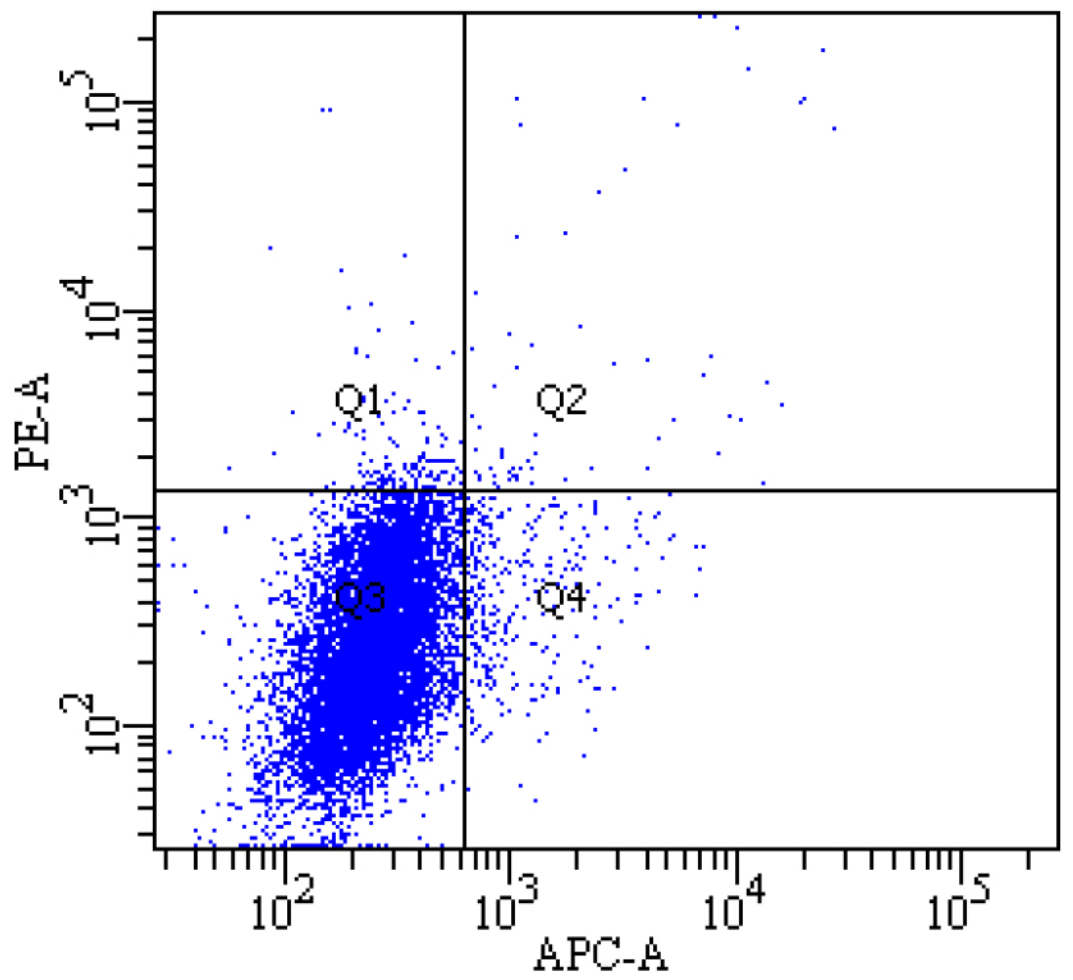

Tube: PBS-1

Population	#Events	%Parent	%Total
All Events	15,204	####	100.0
P1	10,575	69.6	69.6
P2	9,495	89.8	62.5
Q1	160	1.7	1.1
Q2	67	0.7	0.4
Q3	8,925	94.0	58.7
Q4	343	3.6	2.3

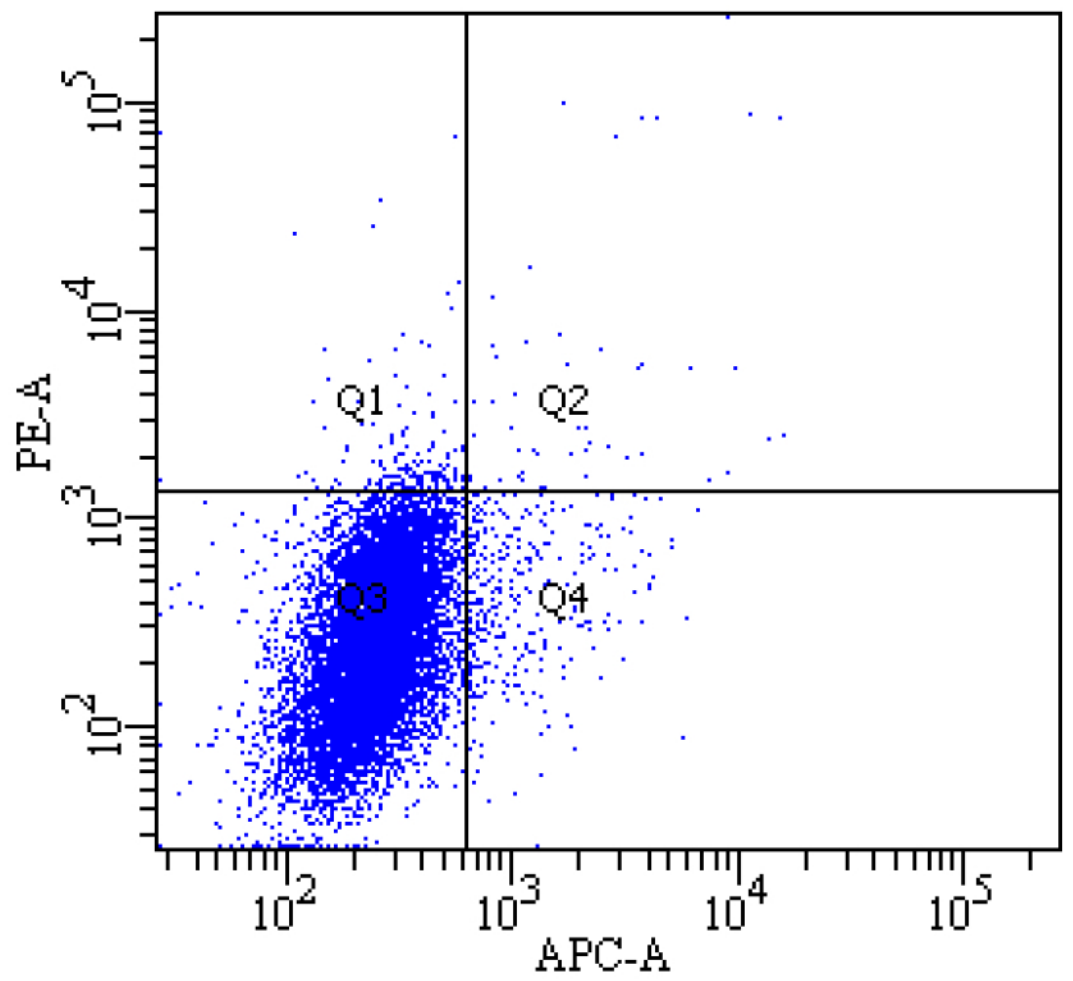

Tube: PBS-2

Population	#Events	%Parent	%Total
All Events	15,038	####	100.0
P1	10,598	70.5	70.5
P2	9,547	90.1	63.5
Q1	176	1.8	1.2
Q2	51	0.5	0.3
Q3	8,980	94.1	59.7
Q4	340	3.6	2.3

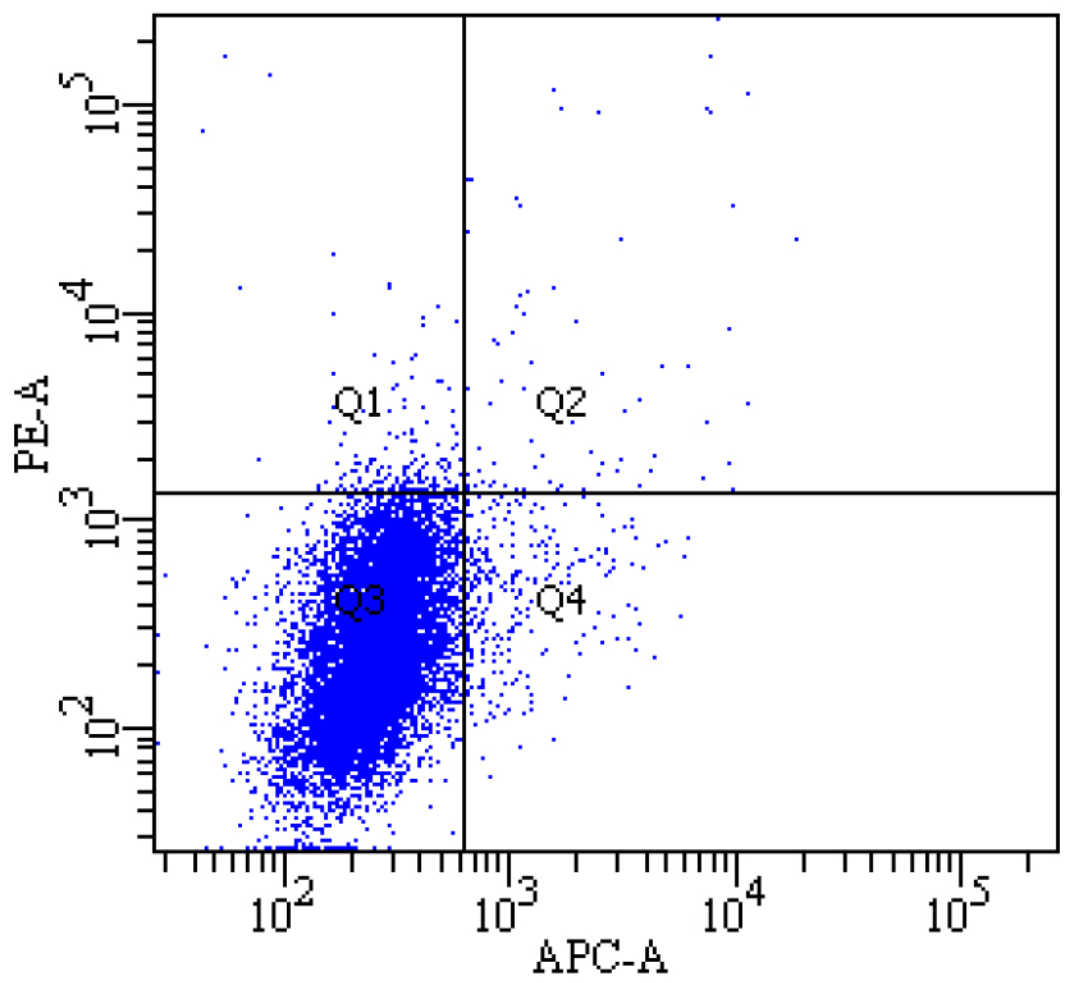

Tube: PBS-3

Population	#Events	%Parent	%Total
All Events	15,116	####	100.0
P1	10,565	69.9	69.9
P2	9,618	91.0	63.6
Q1	172	1.8	1.1
Q2	72	0.7	0.5
Q3	9,098	94.6	60.2
Q4	276	2.9	1.8

A549 NC

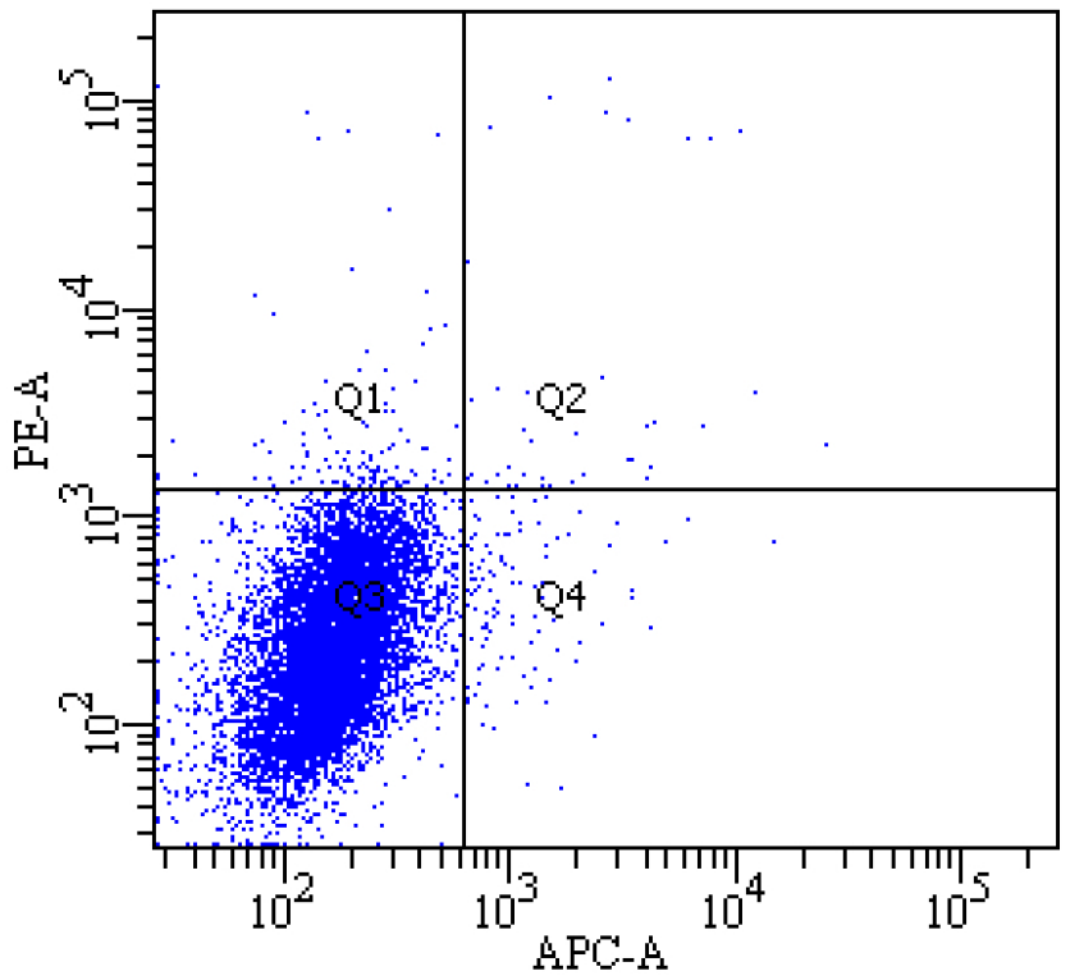

Tube: NC-1

Population	#Events	%Parent	%Total
All Events	15,125	####	100.0
P1	10,000	66.1	66.1
P2	9,224	92.2	61.0
Q1	126	1.4	0.8
Q2	38	0.4	0.3
Q3	8,947	97.0	59.2
Q4	113	1.2	0.7

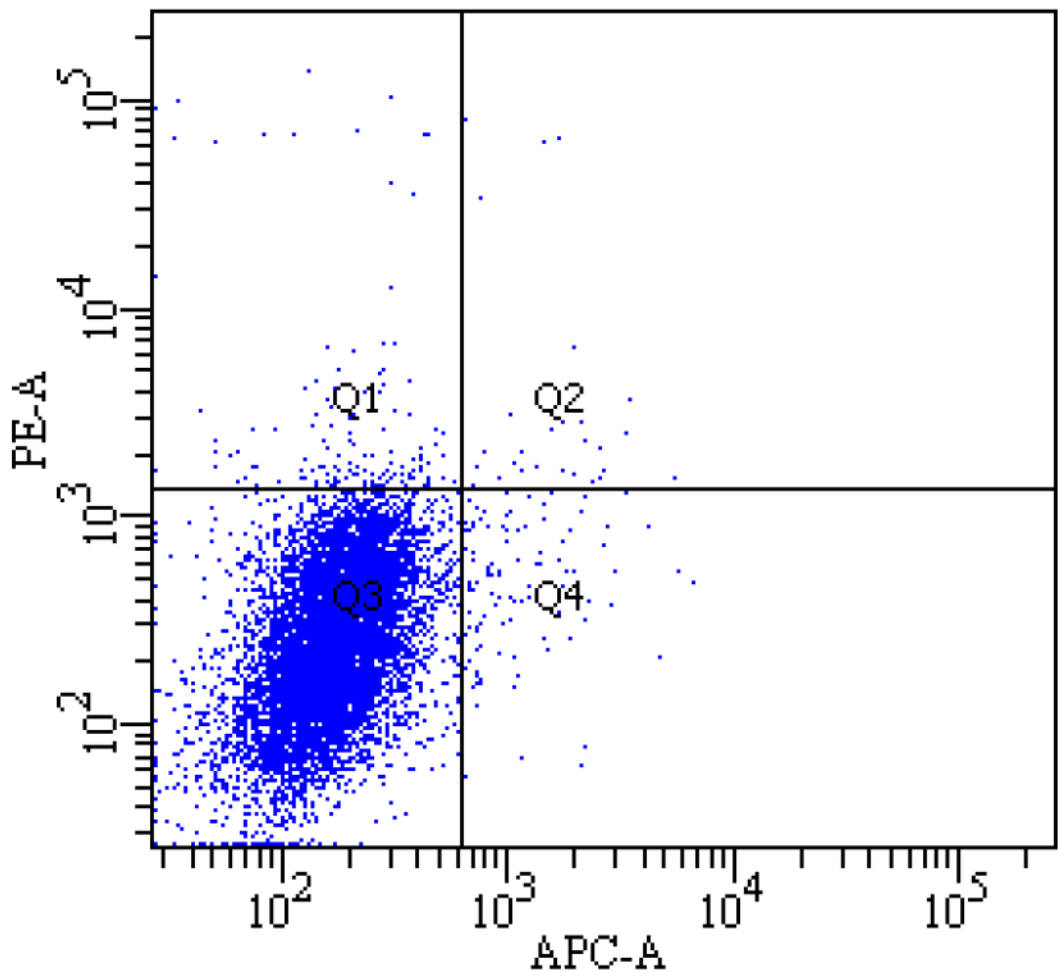

Tube: NC-2

Population	#Events	%Parent	%Total
All Events	15,088	####	100.0
P1	10,000	66.3	66.3
P2	9,218	92.2	61.1
Q1	147	1.6	1.0
Q2	28	0.3	0.2
Q3	8,935	96.9	59.2
Q4	108	1.2	0.7

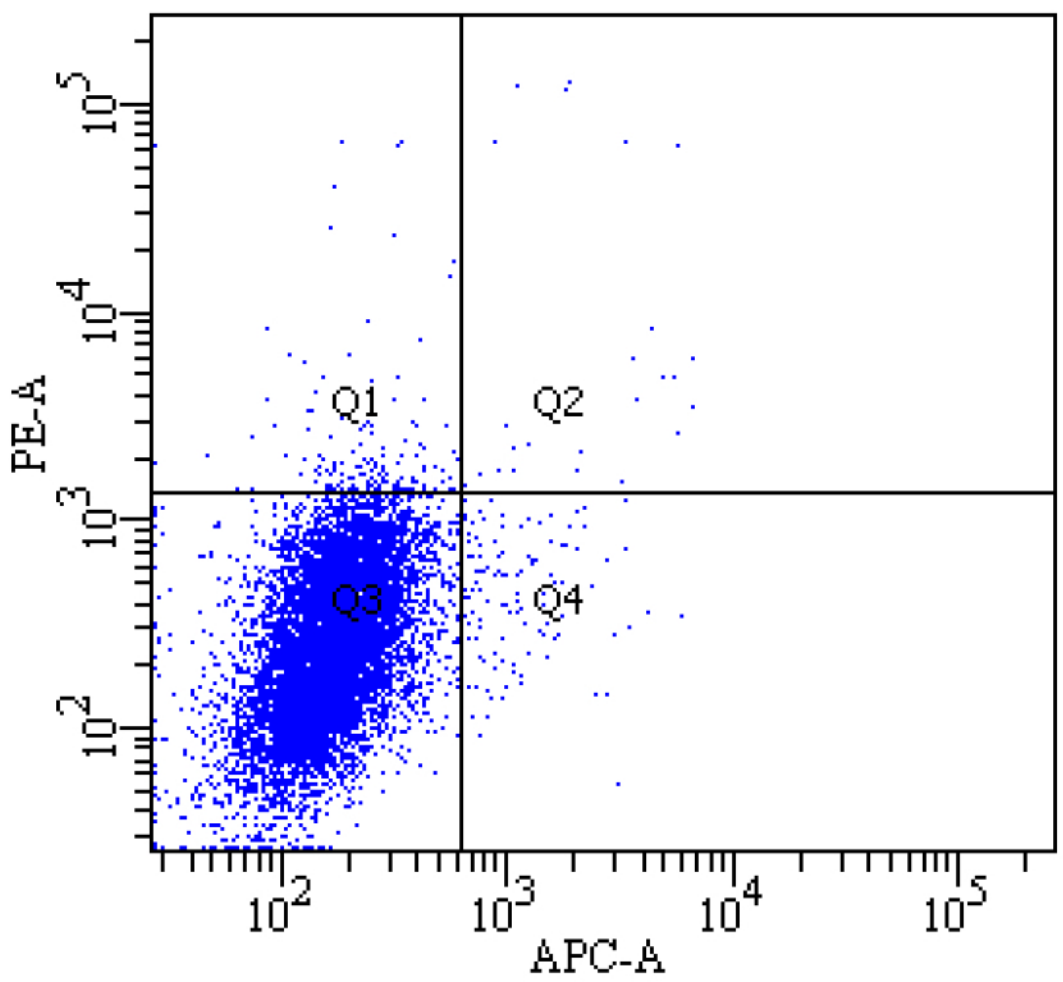

Tube: NC-3

Population	#Events	%Parent	%Total
All Events	14,929	####	100.0
P1	10,000	67.0	67.0
P2	9,314	93.1	62.4
Q1	157	1.7	1.1
Q2	24	0.3	0.2
Q3	9,018	96.8	60.4
Q4	115	1.2	0.8

H1299 NC

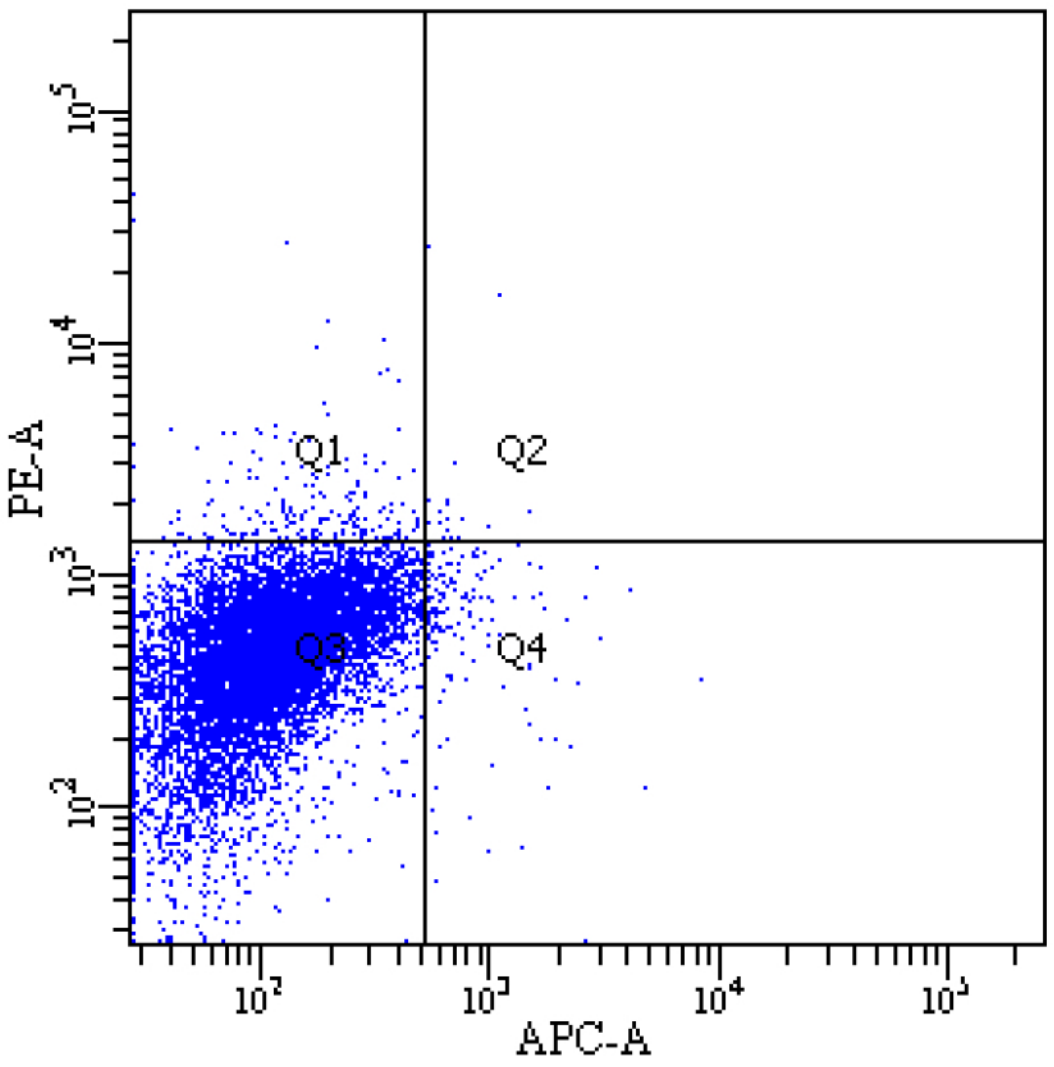

Tube: NC-1

Population	#Events	%Parent	%Total
All Events	16,198	####	100.0
P1	10,471	64.6	64.6
P2	10,066	96.1	62.1
Q1	194	1.9	1.2
Q2	22	0.2	0.1
Q3	9,698	96.3	59.9
Q4	152	1.5	0.9

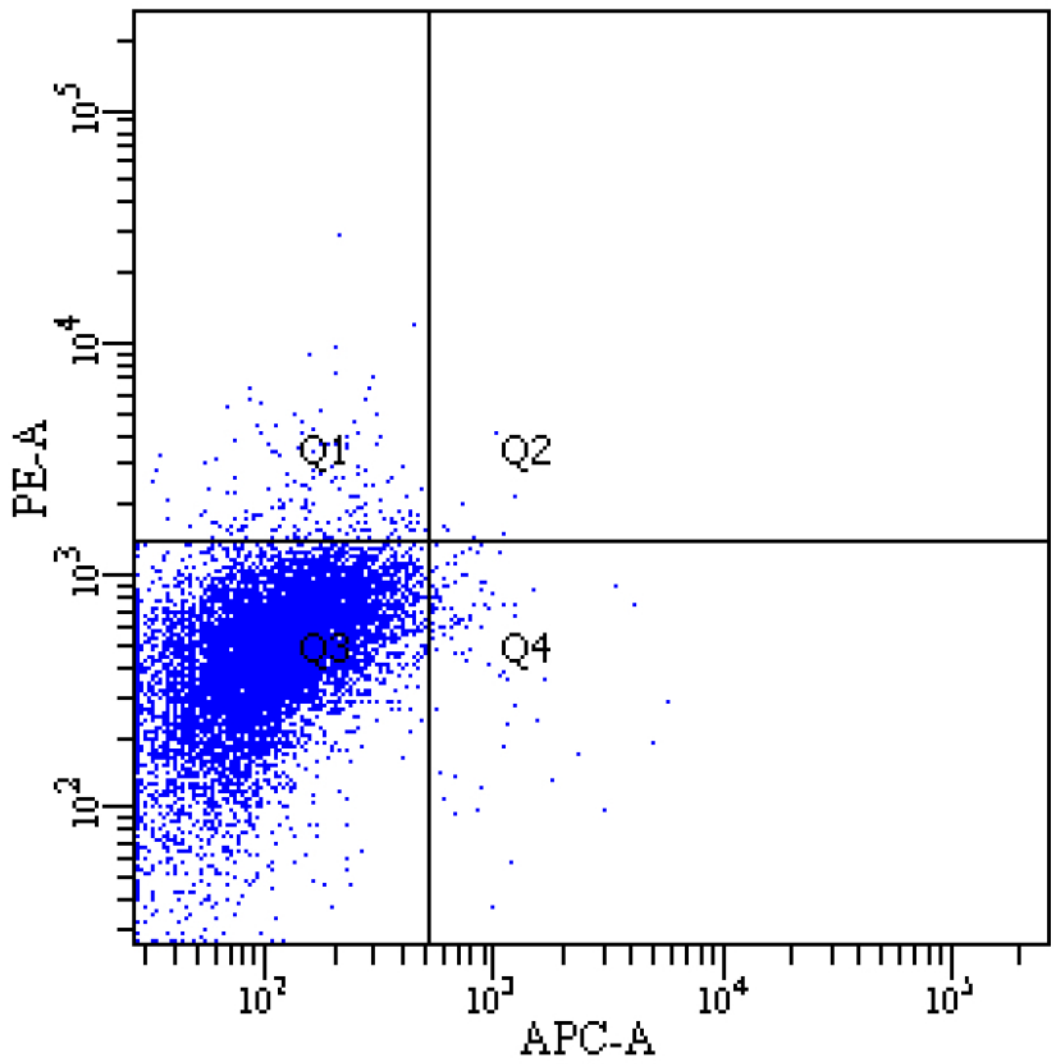

Tube: NC-2

Population	#Events	%Parent	%Total
All Events	16,269	####	100.0
P1	10,474	64.4	64.4
P2	10,098	96.4	62.1
Q1	201	2.0	1.2
Q2	7	0.1	0.0
Q3	9,791	97.0	60.2
Q4	99	1.0	0.6

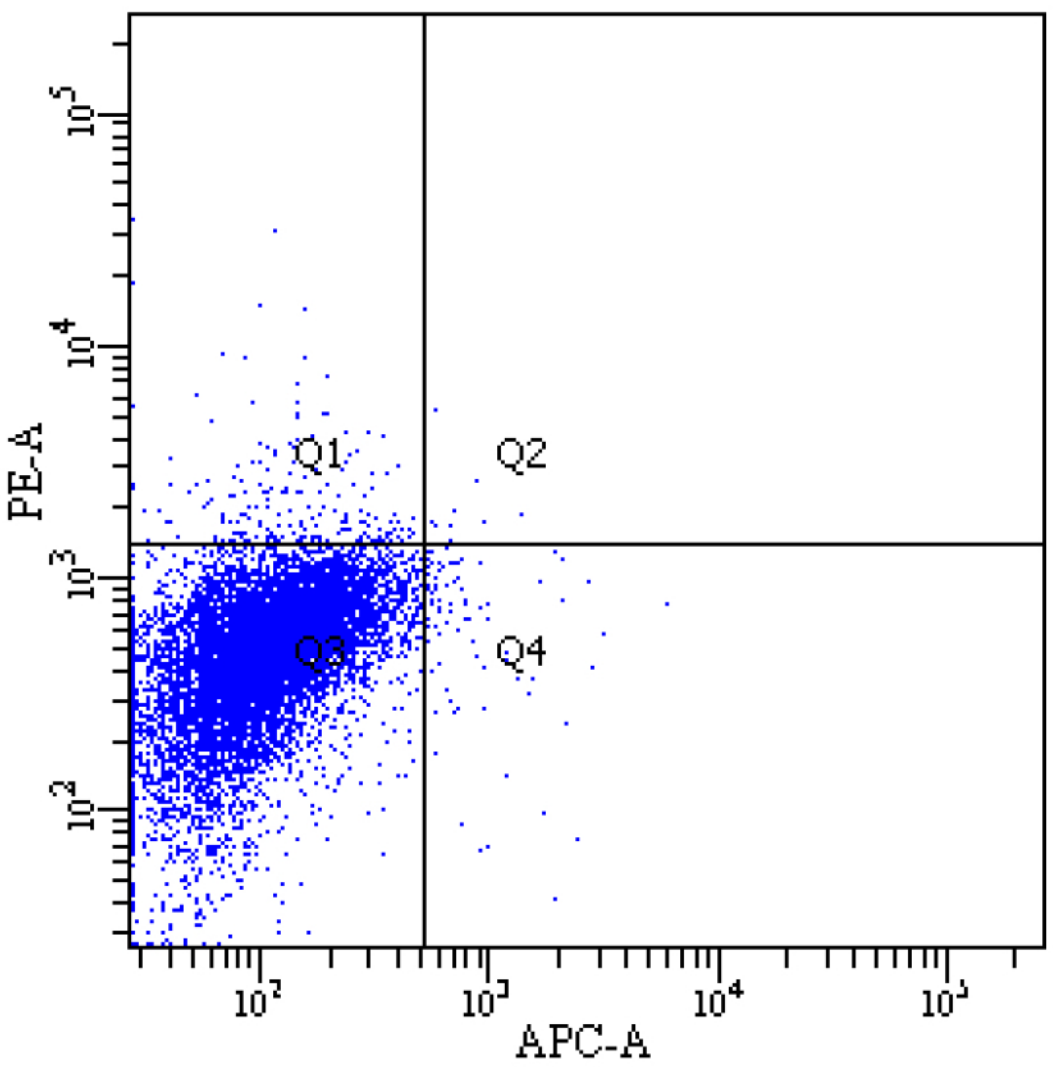

Tube: NC-3

Population	#Events	%Parent	%Total
All Events	16,077	####	100.0
P1	10,470	65.1	65.1
P2	10,106	96.5	62.9
Q1	200	2.0	1.2
Q2	9	0.1	0.1
Q3	9,812	97.1	61.0
Q4	85	0.8	0.5

H1299 OE

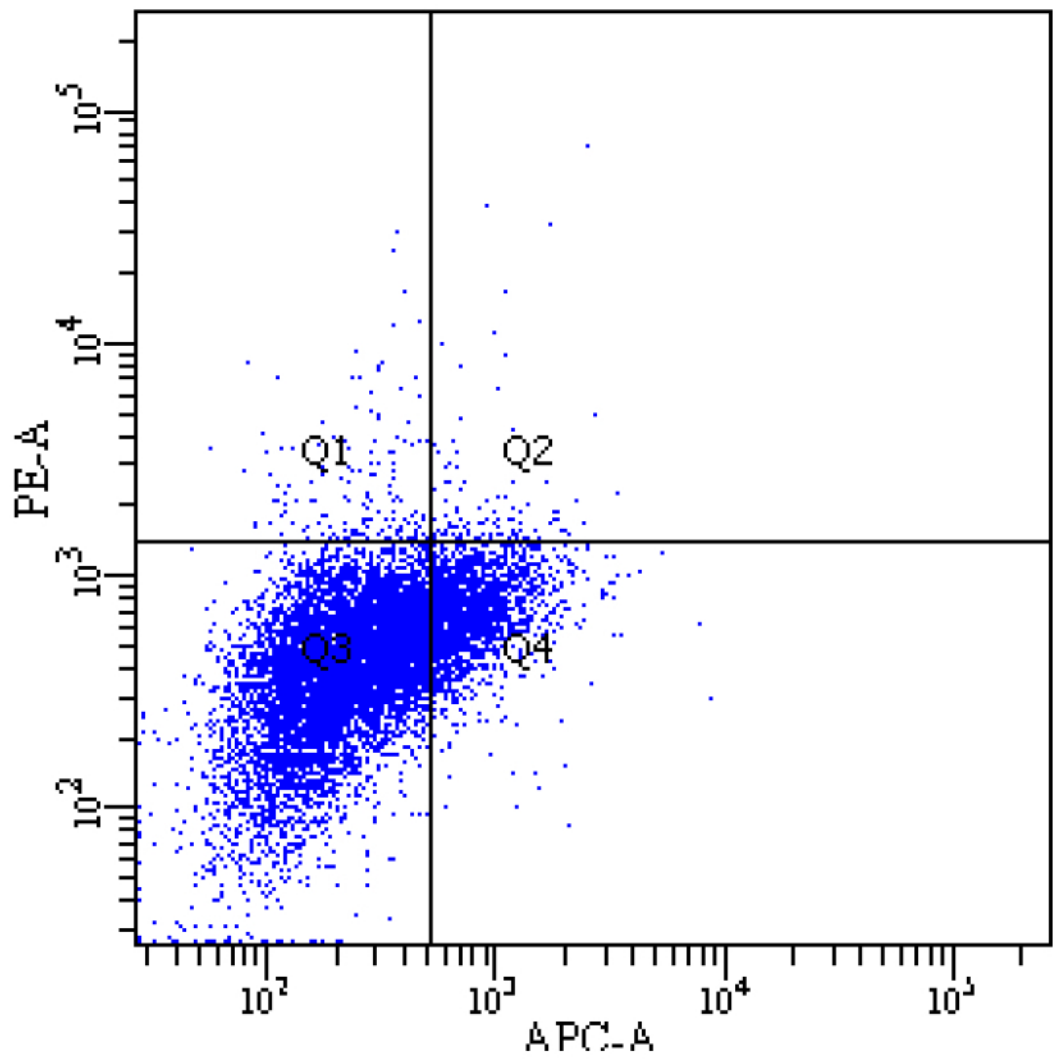

Tube: OX-1

Population	#Events	%Parent	%Total
All Events	16,413	####	100.0
P1	10,399	63.4	63.4
P2	9,951	95.7	60.6
Q1	153	1.5	0.9
Q2	110	1.1	0.7
Q3	7,827	78.7	47.7
Q4	1,861	18.7	11.3

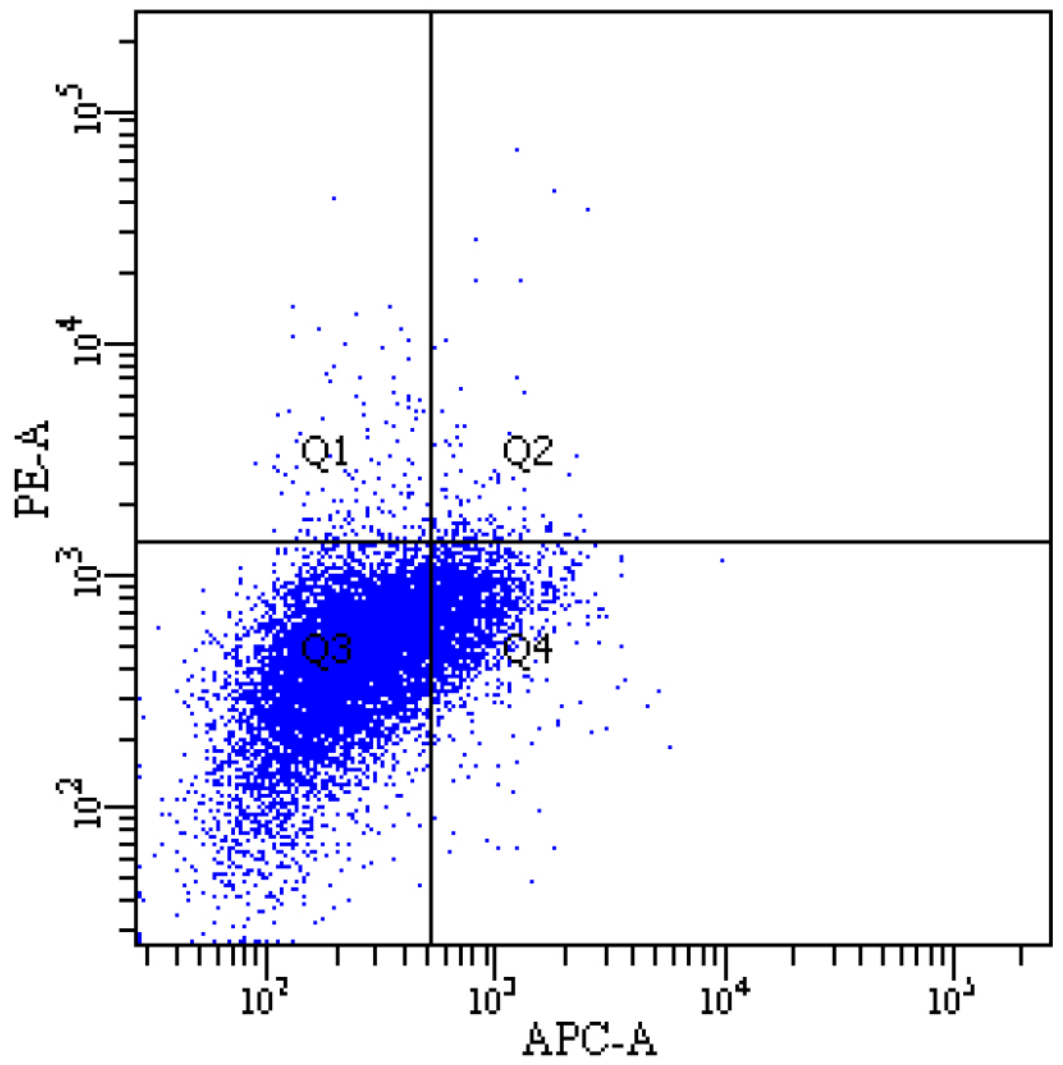

Tube: OX-2

Population	#Events	%Parent	%Total
All Events	16,189	####	100.0
P1	10,399	64.2	64.2
P2	9,961	95.8	61.5
Q1	151	1.5	0.9
Q2	124	1.2	0.8
Q3	7,914	79.4	48.9
Q4	1,772	17.8	10.9

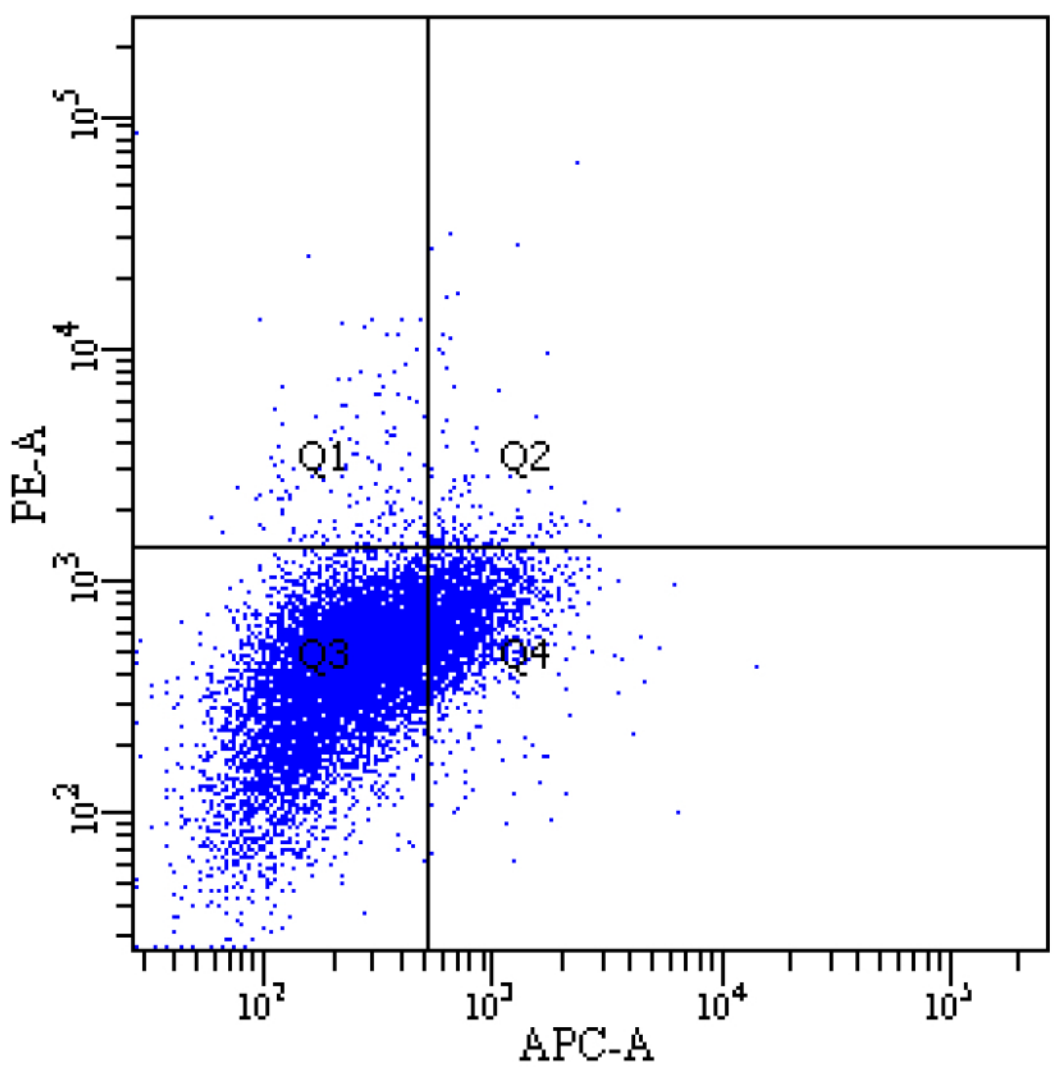

Tube: OX-3

Population	#Events	%Parent	%Total
All Events	16,346	####	100.0
P1	10,393	63.6	63.6
P2	9,979	96.0	61.0
Q1	160	1.6	1.0
Q2	136	1.4	0.8
Q3	7,894	79.1	48.3
Q4	1,789	17.9	10.9

H1299 PBS

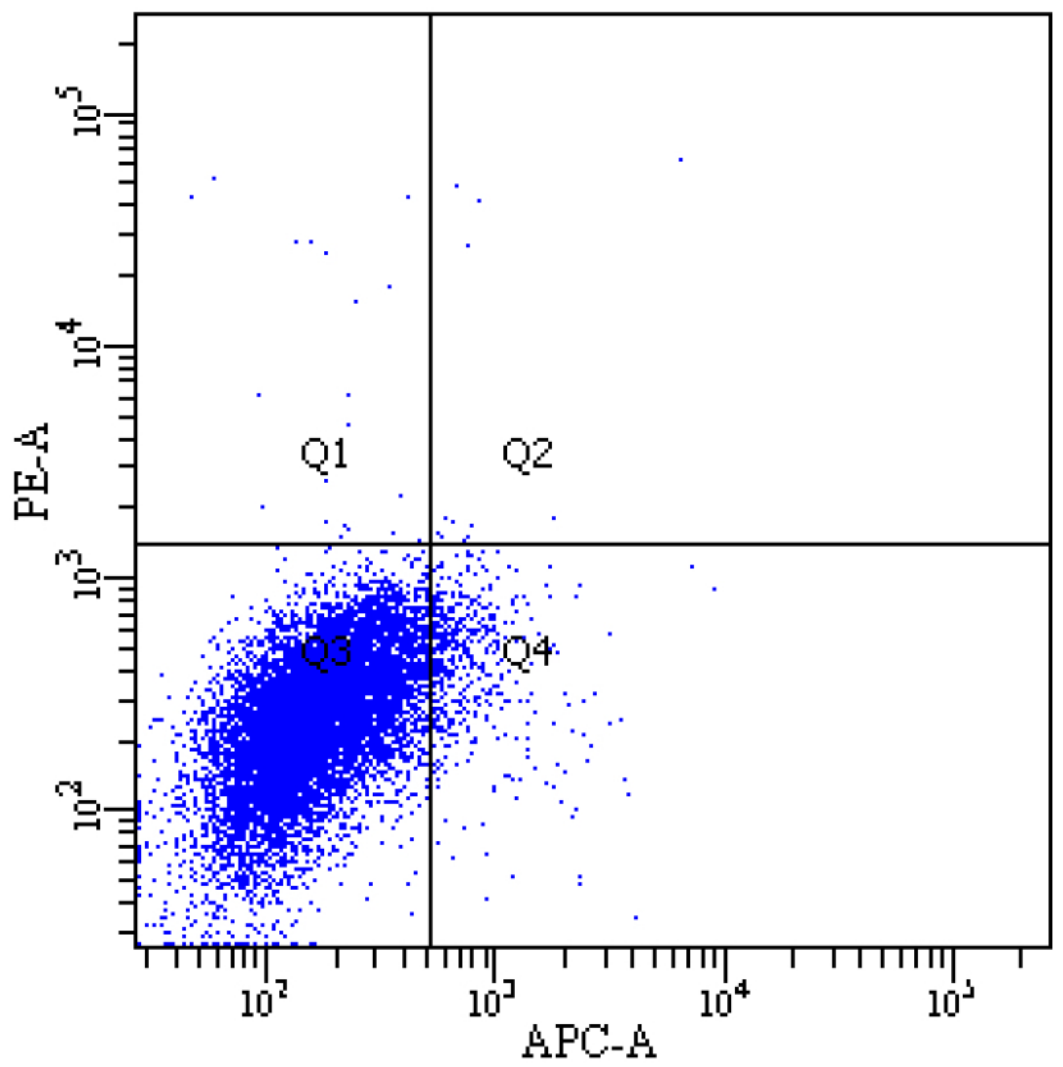

Tube: PBS-1

Population	#Events	%Parent	%Total
All Events	13,330	####	100.0
P1	10,184	76.4	76.4
P2	9,819	96.4	73.7
Q1	22	0.2	0.2
Q2	12	0.1	0.1
Q3	9,310	94.8	69.8
Q4	475	4.8	3.6

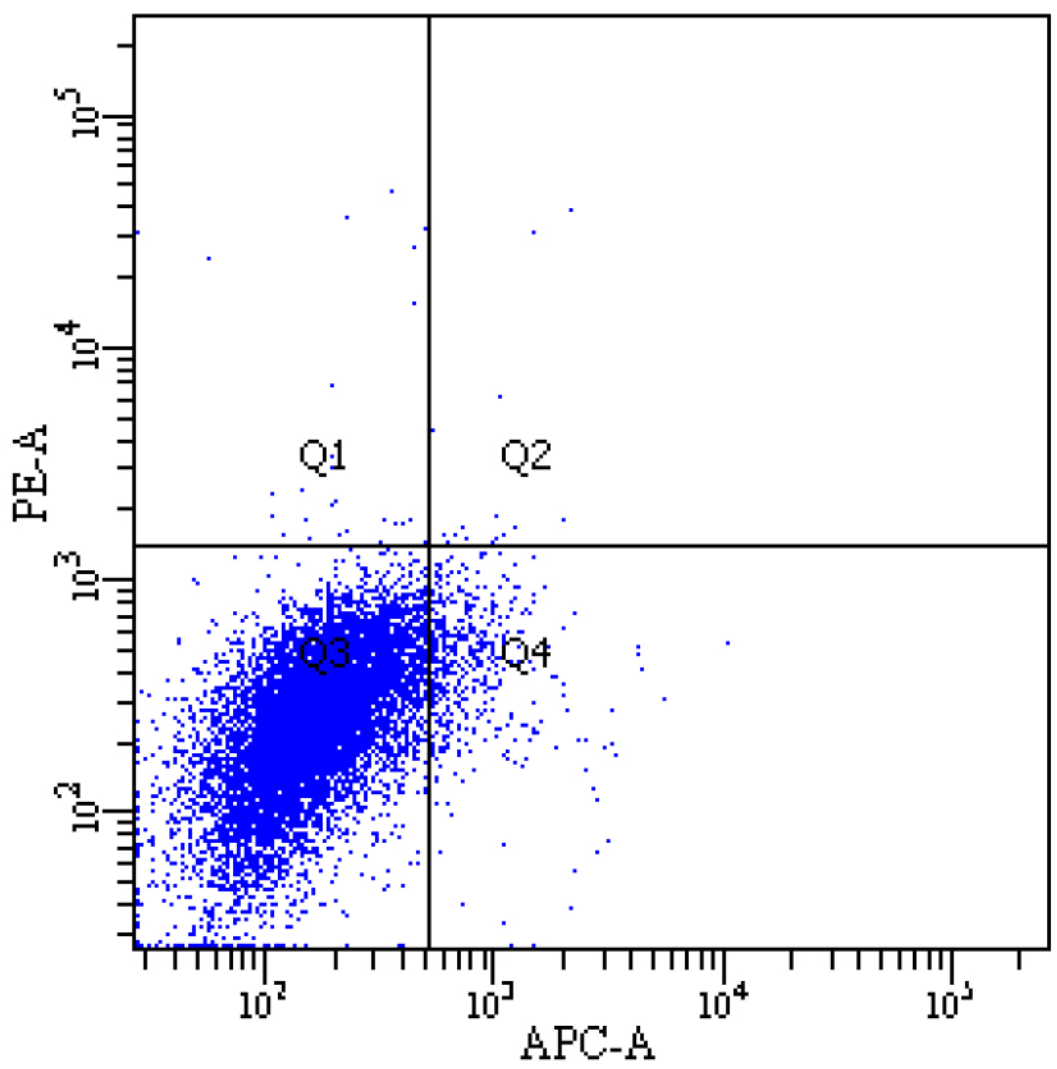

Tube: PBS-2

Population	#Events	%Parent	%Total
All Events	13,365	####	100.0
P1	10,217	76.4	76.4
P2	9,835	96.3	73.6
Q1	29	0.3	0.2
Q2	16	0.2	0.1
Q3	9,300	94.6	69.6
Q4	490	5.0	3.7

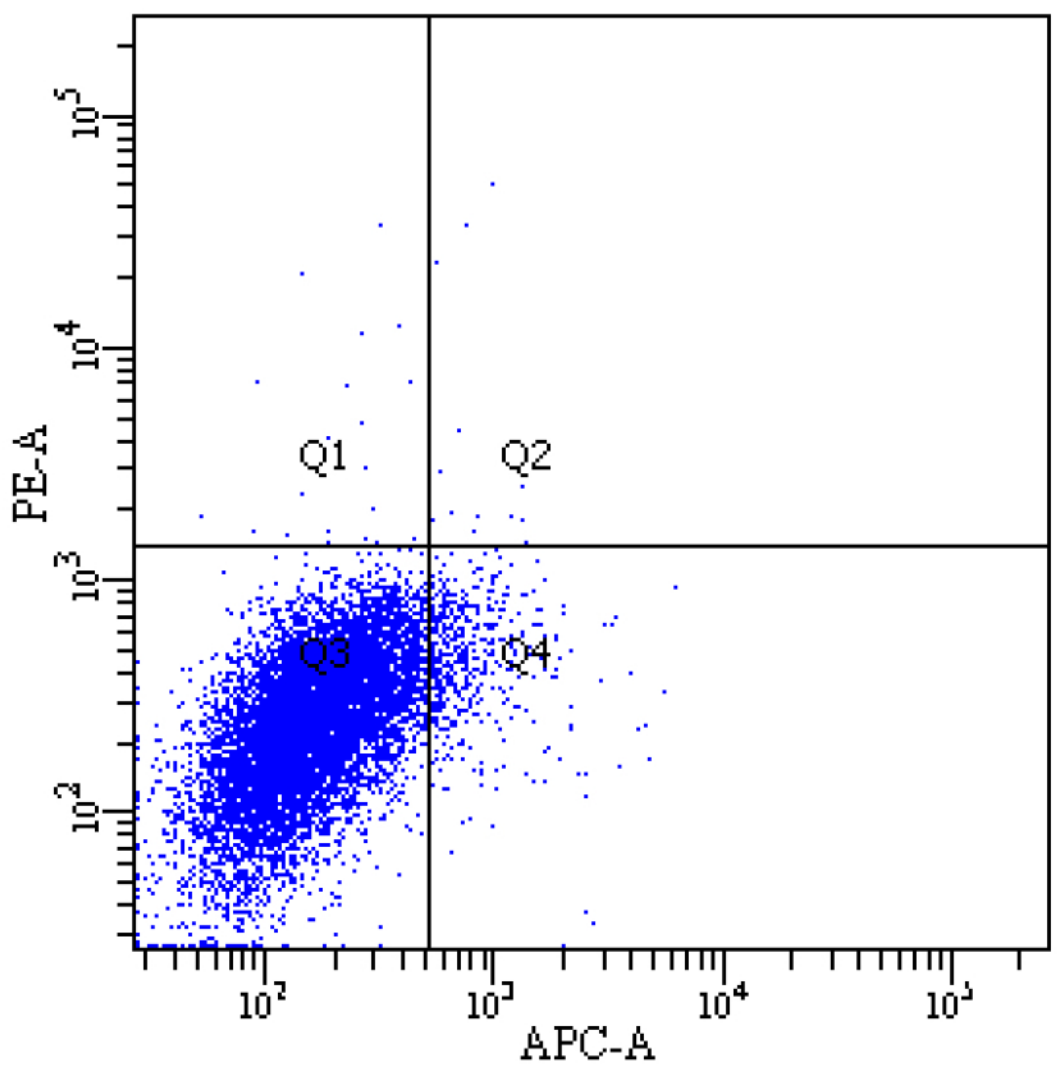

Tube: PBS-3

Population	#Events	%Parent	%Total
All Events	13,185	####	100.0
P1	10,174	77.2	77.2
P2	9,759	95.9	74.0
Q1	21	0.2	0.2
Q2	13	0.1	0.1
Q3	9,224	94.5	70.0
Q4	501	5.1	3.8
