# Supplementary material for: CDC73 suppresses lung adenocarcinoma via the PTEN/AKT pathway and exhibits biomarker and functional potential in extracellular vesicles
Source: Cell Death Discov. 2026 Aug 14;12:412. doi: 10.1038/s41420-026-03305-6 (PMC13652814; doi:10.1038/s41420-026-03305-6)
Supplement: Supplementary file 2 [file 41420_2026_3305_MOESM3_ESM.pdf]

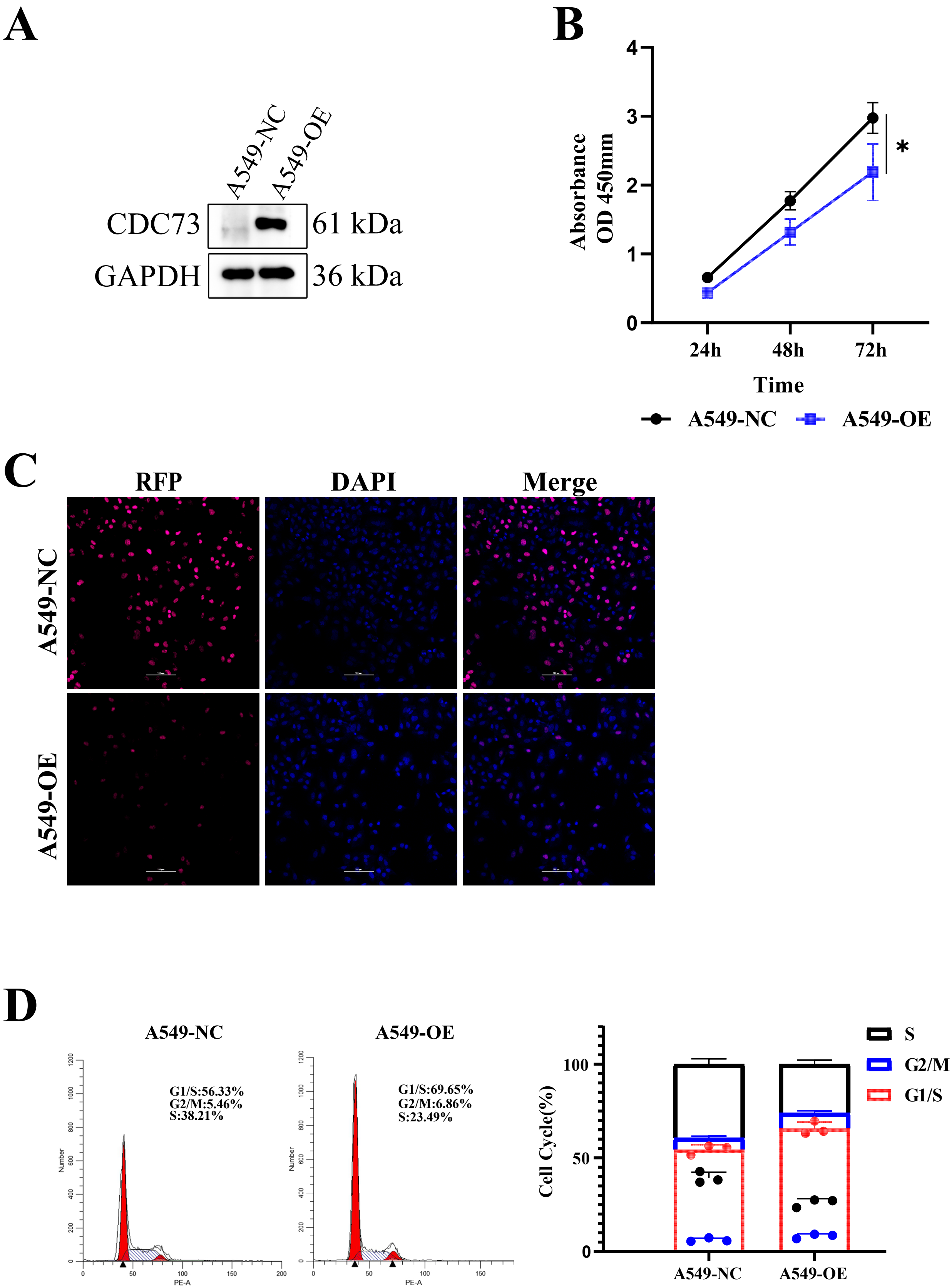

A
A549-NC
A549-OE
CDC73
61 kDa
GAPDH
36 kDa
B
Absorbance
OD 450mm
4
3
2
1
0
*
24h
48h
72h
Time
A549-NC
A549-OE
C
RFP
DAPI
Merge
A549-NC
A549-OE
D
A549-NC
A549-OE
G1/S:56.33%
G2/M:5.46%
S:38.21%
G1/S:69.65%
G2/M:6.86%
S:23.49%
Number
PE-A
Cell Cycle(%)
100
50
0
S
G2/M
G1/S
A549-NC
A549-OE
