# Supplementary material for: CDC73 suppresses lung adenocarcinoma via the PTEN/AKT pathway and exhibits biomarker and functional potential in extracellular vesicles
Source: Cell Death Discov. 2026 Aug 14;12:412. doi: 10.1038/s41420-026-03305-6 (PMC13652814; doi:10.1038/s41420-026-03305-6)
Supplement: Supplementary file 3 [file 41420_2026_3305_MOESM4_ESM.pdf]

A

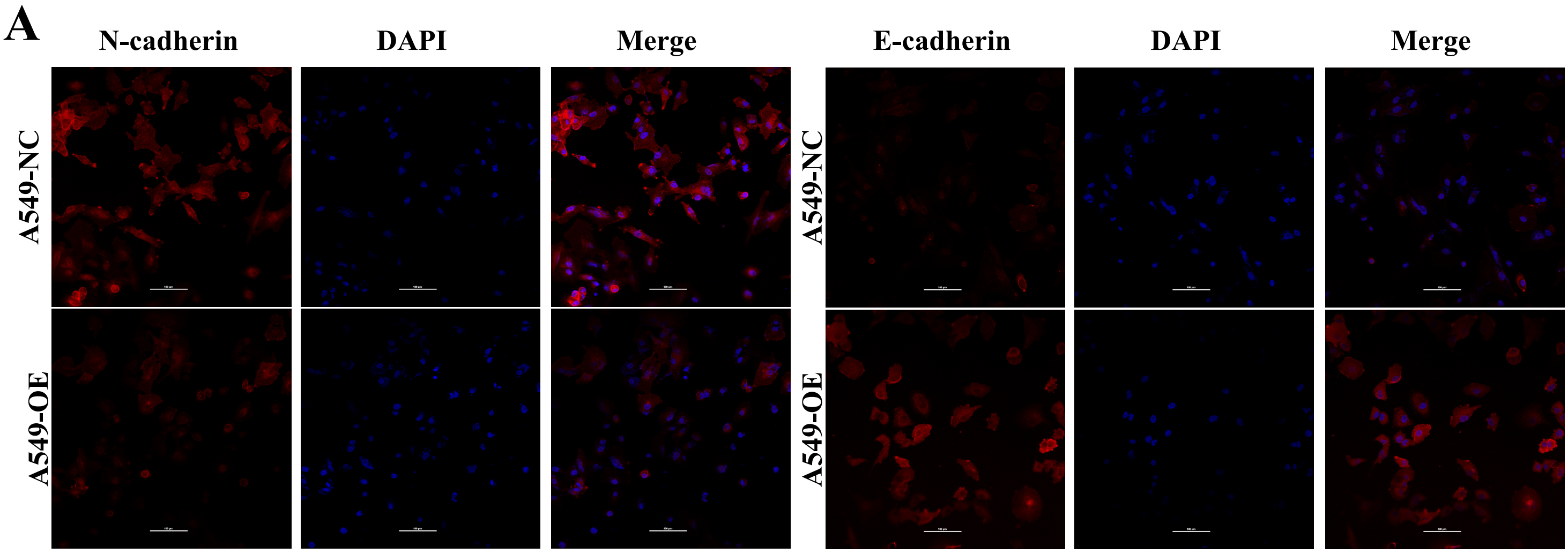
N-cadherin
DAPI
Merge
E-cadherin
DAPI
Merge
A549-NC
A549-OE
A549-NC
A549-OE

B

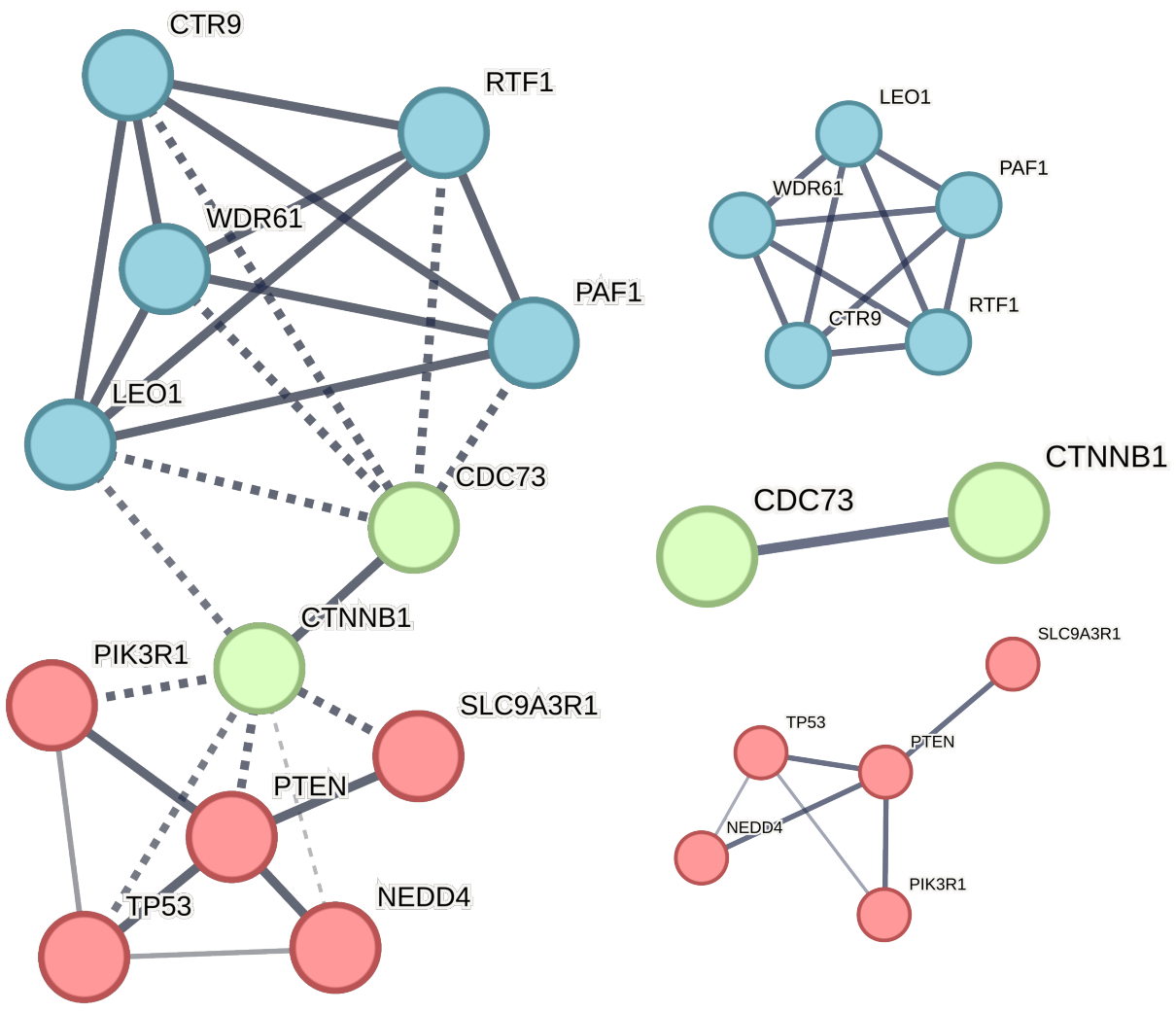
CTR9
RTF1
WDR61
PAF1
LEO1
CDC73
CTNNB1
PIK3R1
SLC9A3R1
PTEN
TP53
NEDD4
LEO1
WDR61
PAF1
CTR9
RTF1
CDC73
CTNNB1
SLC9A3R1
TP53
PTEN
NEDD4
PIK3R1

C

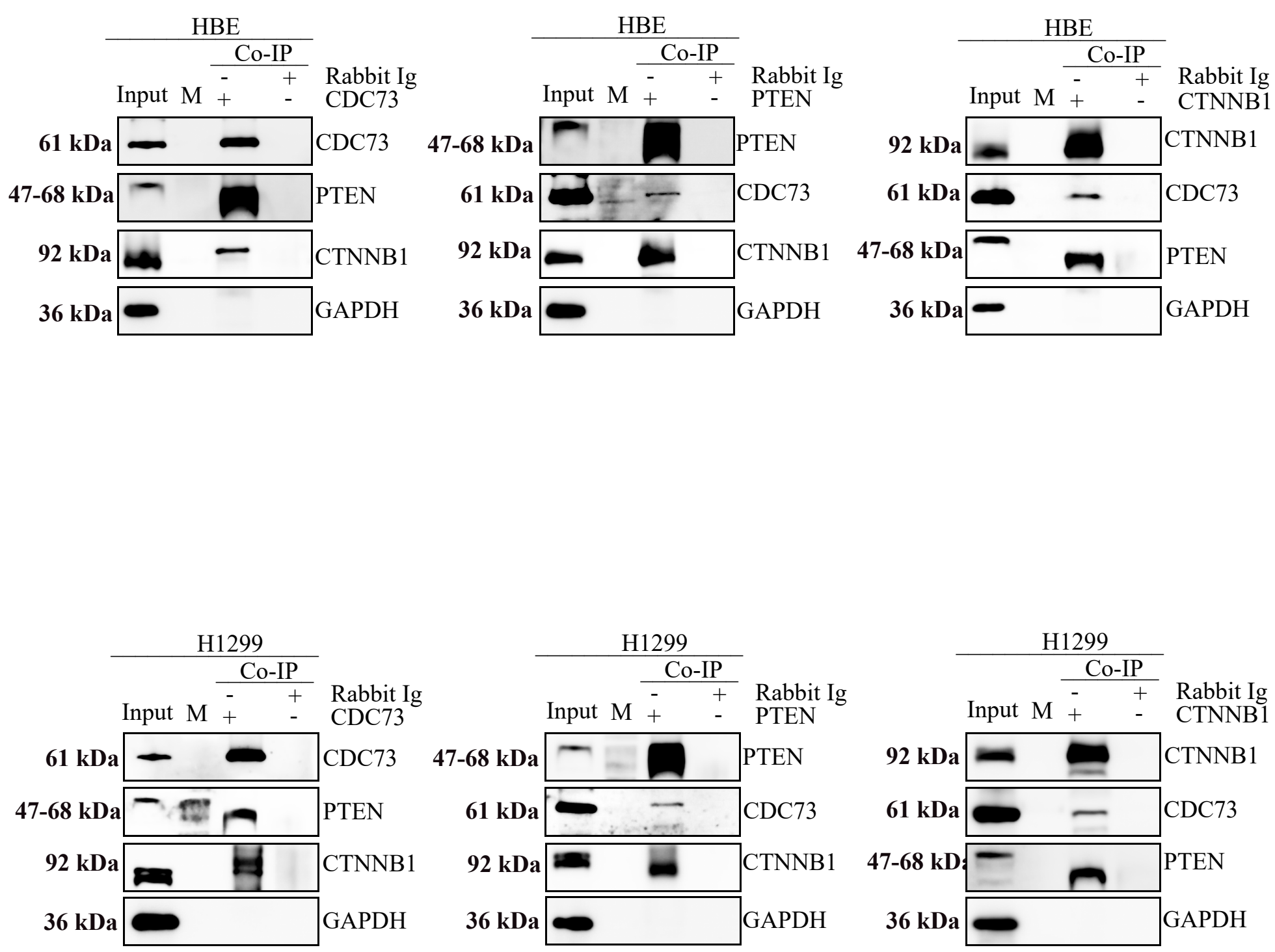
HBE
Co-IP
Input M
- +
+ -
Rabbit Ig
CDC73
61 kDa
47-68 kDa
92 kDa
36 kDa
CDC73
PTEN
CTNNB1
GAPDH
HBE
Co-IP
Rabbit Ig
PTEN
47-68 kDa
61 kDa
92 kDa
36 kDa
PTEN
CDC73
CTNNB1
GAPDH
HBE
Co-IP
Rabbit Ig
CTNNB1
92 kDa
61 kDa
47-68 kDa
36 kDa
CTNNB1
CDC73
PTEN
GAPDH
H1299
Co-IP
Rabbit Ig
CDC73
61 kDa
47-68 kDa
92 kDa
36 kDa
CDC73
PTEN
CTNNB1
GAPDH
H1299
Co-IP
Rabbit Ig
PTEN
47-68 kDa
61 kDa
92 kDa
36 kDa
PTEN
CDC73
CTNNB1
GAPDH
H1299
Co-IP
Rabbit Ig
CTNNB1
92 kDa
61 kDa
47-68 kDa
36 kDa
CTNNB1
CDC73
PTEN
GAPDH
