# Supplementary material for: CDC73 suppresses lung adenocarcinoma via the PTEN/AKT pathway and exhibits biomarker and functional potential in extracellular vesicles
Source: Cell Death Discov. 2026 Aug 14;12:412. doi: 10.1038/s41420-026-03305-6 (PMC13652814; doi:10.1038/s41420-026-03305-6)
Supplement: Supplementary file 4 [file 41420_2026_3305_MOESM5_ESM.pdf]

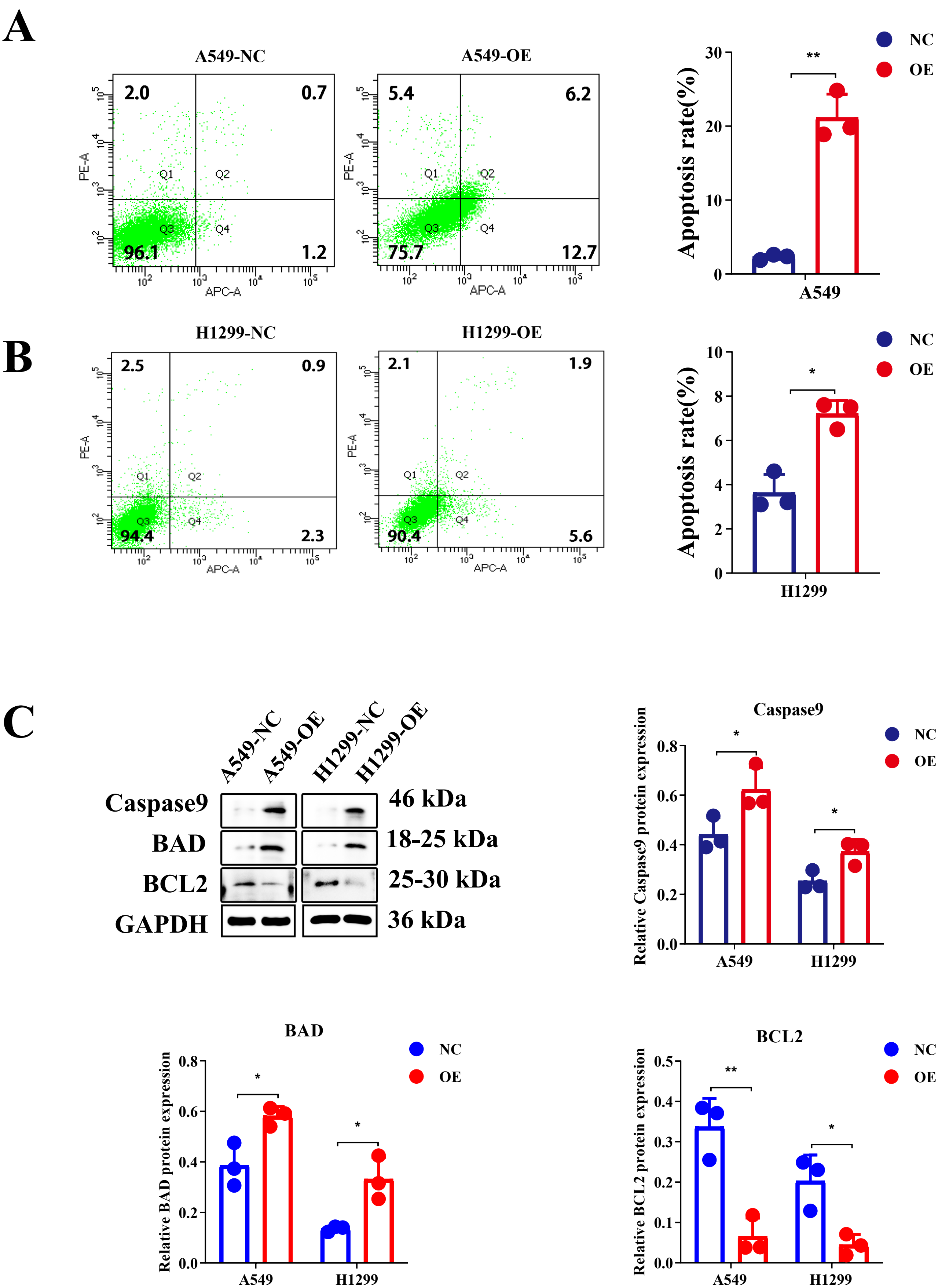
A
A549-NC
A549-OE
2.0
0.7
96.1
1.2
5.4
6.2
75.7
12.7
Q1
Q2
Q3
Q4
PE-A
APC-A
NC
OE
**
Apoptosis rate(%)
A549
B
H1299-NC
H1299-OE
2.5
0.9
94.4
2.3
2.1
1.9
90.4
5.6
*
H1299
C
A549-NC
A549-OE
H1299-NC
H1299-OE
Caspase9
BAD
BCL2
GAPDH
46 kDa
18-25 kDa
25-30 kDa
36 kDa
Caspase9
Relative Caspase9 protein expression
BAD
Relative BAD protein expression
BCL2
Relative BCL2 protein expression
