# Supplementary material for: CDC73 suppresses lung adenocarcinoma via the PTEN/AKT pathway and exhibits biomarker and functional potential in extracellular vesicles
Source: Cell Death Discov. 2026 Aug 14;12:412. doi: 10.1038/s41420-026-03305-6 (PMC13652814; doi:10.1038/s41420-026-03305-6)
Supplement: Supplementary file 5 [file 41420_2026_3305_MOESM6_ESM.pdf]

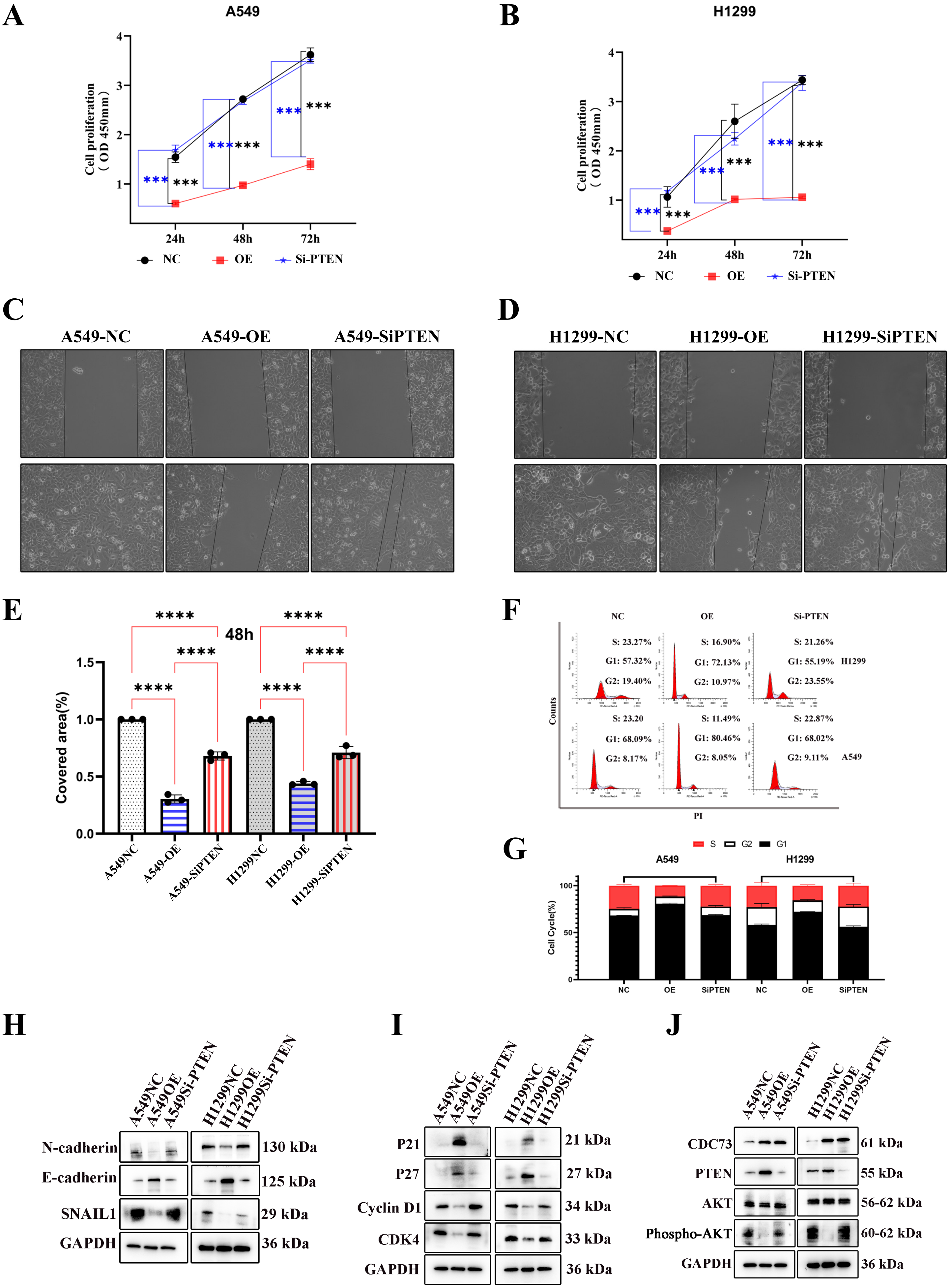
A
A549
Cell proliferation (OD 450mm)
24h
48h
72h
NC
OE
Si-PTEN
B
H1299
Cell proliferation (OD 450mm)
C
A549-NC
A549-OE
A549-SiPTEN
D
H1299-NC
H1299-OE
H1299-SiPTEN
E
48h
Covered area(%)
A549NC
A549-OE
A549-SiPTEN
H1299NC
H1299-OE
H1299-SiPTEN
F
NC
OE
Si-PTEN
S: 23.27%
G1: 57.32%
G2: 19.40%
S: 16.90%
G1: 72.13%
G2: 10.97%
S: 21.26%
G1: 55.19%
G2: 23.55%
H1299
S: 23.20
G1: 68.09%
G2: 8.17%
S: 11.49%
G1: 80.46%
G2: 8.05%
S: 22.87%
G1: 68.02%
G2: 9.11%
A549
Counts
PI
G
S
G2
G1
A549
H1299
Cell Cycle(%)
NC
OE
SiPTEN
H
A549NC
A549OE
A549Si-PTEN
H1299NC
H1299OE
H1299Si-PTEN
N-cadherin
E-cadherin
SNAIL1
GAPDH
130 kDa
125 kDa
29 kDa
36 kDa
I
P21
P27
Cyclin D1
CDK4
GAPDH
21 kDa
27 kDa
34 kDa
33 kDa
36 kDa
J
CDC73
PTEN
AKT
Phospho-AKT
GAPDH
61 kDa
55 kDa
56-62 kDa
60-62 kDa
36 kDa
