# Supplementary material for: CDC73 suppresses lung adenocarcinoma via the PTEN/AKT pathway and exhibits biomarker and functional potential in extracellular vesicles
Source: Cell Death Discov. 2026 Aug 14;12:412. doi: 10.1038/s41420-026-03305-6 (PMC13652814; doi:10.1038/s41420-026-03305-6)
Supplement: Supplementary file 6 [file 41420_2026_3305_MOESM7_ESM.pdf]

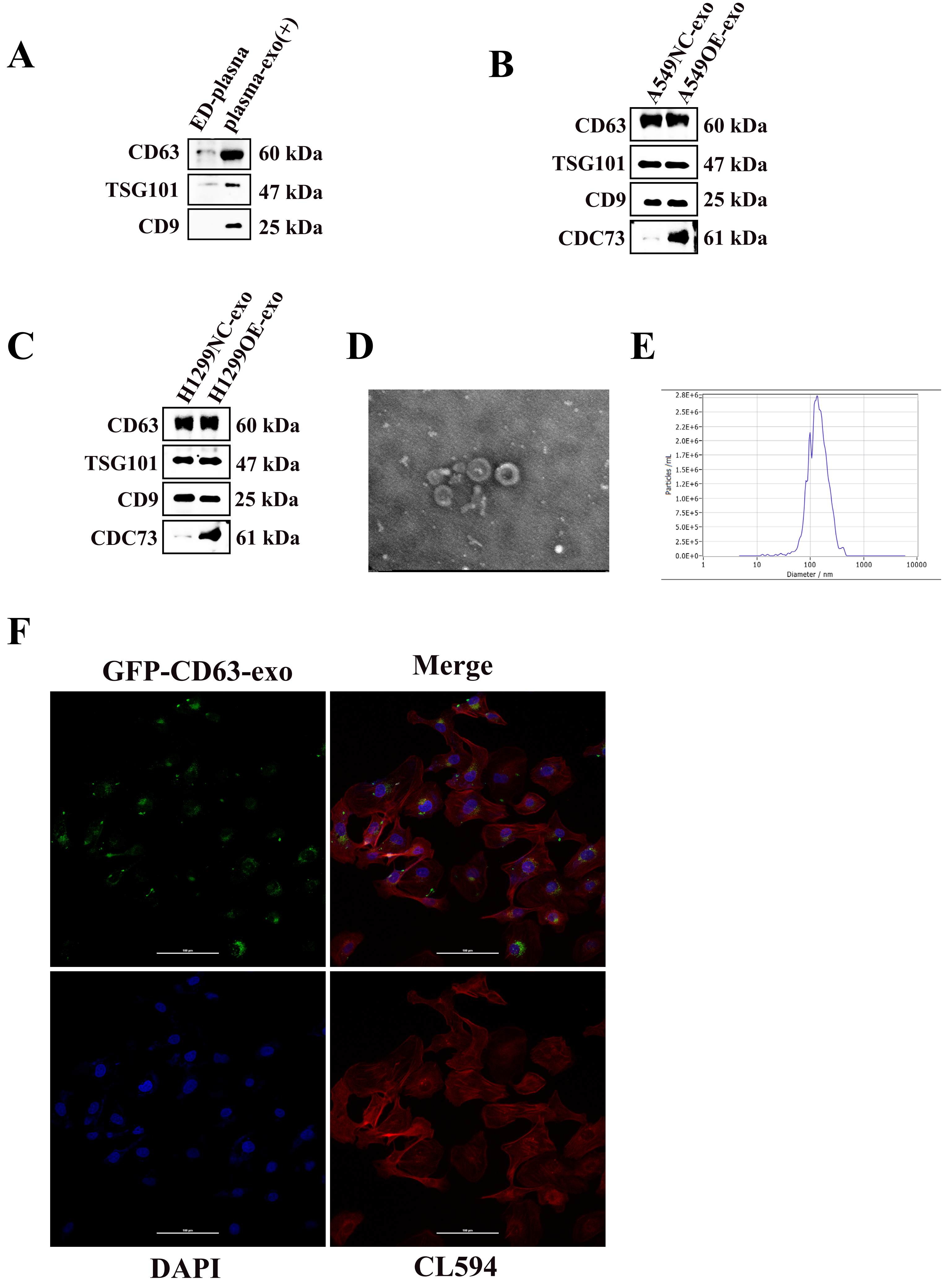
A
ED-plasna
plasma-exo(+)
CD63
60 kDa
TSG101
47 kDa
CD9
25 kDa
B
A549NC-exo
A549OE-exo
CD63
60 kDa
TSG101
47 kDa
CD9
25 kDa
CDC73
61 kDa
C
H1299NC-exo
H1299OE-exo
CD63
60 kDa
TSG101
47 kDa
CD9
25 kDa
CDC73
61 kDa
D
E
Particles /mL
Diameter / nm
1
10
100
1000
10000
F
GFP-CD63-exo
Merge
DAPI
CL594
