# Supplementary material for: CDC73 suppresses lung adenocarcinoma via the PTEN/AKT pathway and exhibits biomarker and functional potential in extracellular vesicles
Source: Cell Death Discov. 2026 Aug 14;12:412. doi: 10.1038/s41420-026-03305-6 (PMC13652814; doi:10.1038/s41420-026-03305-6)
Supplement: Supplementary file 7 [file 41420_2026_3305_MOESM8_ESM.pdf]

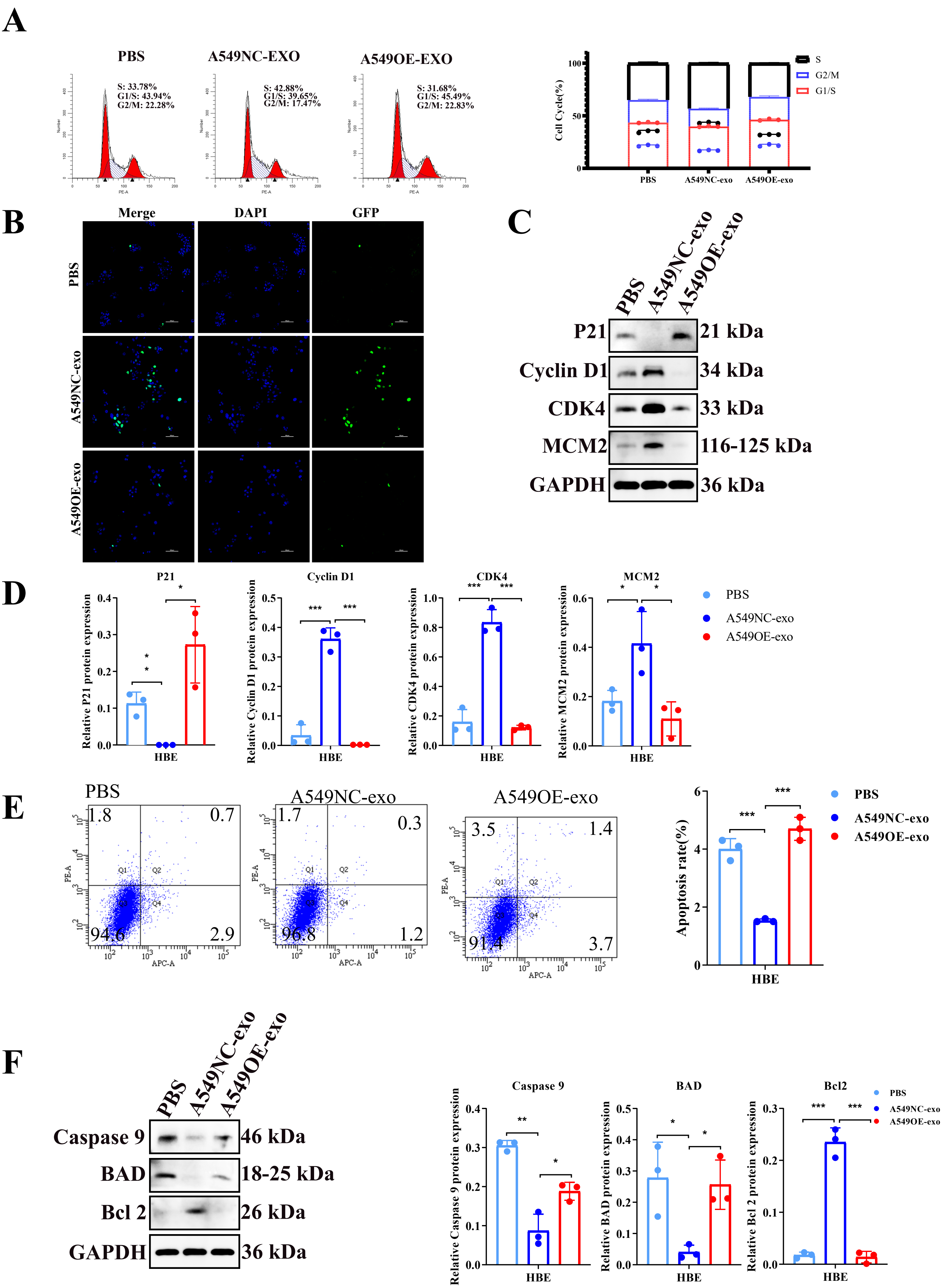

A
PBS
A549NC-EXO
A549OE-EXO
S: 33.78%
G1/S: 43.94%
G2/M: 22.28%
S: 42.88%
G1/S: 39.65%
G2/M: 17.47%
S: 31.68%
G1/S: 45.49%
G2/M: 22.83%
Cell Cycle(%)
S
G2/M
G1/S
PBS
A549NC-exo
A549OE-exo
B
Merge
DAPI
GFP
PBS
A549NC-exo
A549OE-exo
C
PBS
A549NC-exo
A549OE-exo
P21
21 kDa
Cyclin D1
34 kDa
CDK4
33 kDa
MCM2
116-125 kDa
GAPDH
36 kDa
D
P21
Relative P21 protein expression
Cyclin D1
Relative Cyclin D1 protein expression
CDK4
Relative CDK4 protein expression
MCM2
Relative MCM2 protein expression
HBE
PBS
A549NC-exo
A549OE-exo
E
PBS
A549NC-exo
A549OE-exo
1.8
0.7
94.6
2.9
1.7
0.3
96.8
1.2
3.5
1.4
91.4
3.7
PE-A
APC-A
Apoptosis rate(%)
HBE
PBS
A549NC-exo
A549OE-exo
F
PBS
A549NC-exo
A549OE-exo
Caspase 9
46 kDa
BAD
18-25 kDa
Bcl 2
26 kDa
GAPDH
36 kDa
Caspase 9
Relative Caspase 9 protein expression
BAD
Relative BAD protein expression
Bcl2
Relative Bcl 2 protein expression
HBE
PBS
A549NC-exo
A549OE-exo
