# Supplementary material for: CDC73 suppresses lung adenocarcinoma via the PTEN/AKT pathway and exhibits biomarker and functional potential in extracellular vesicles
Source: Cell Death Discov. 2026 Aug 14;12:412. doi: 10.1038/s41420-026-03305-6 (PMC13652814; doi:10.1038/s41420-026-03305-6)
Supplement: Supplementary file 8 [file 41420_2026_3305_MOESM9_ESM.pdf]

The expression of CDC73 (MW 61kDa) in clinical sample normalized to TSG101 (MW 47kDa)

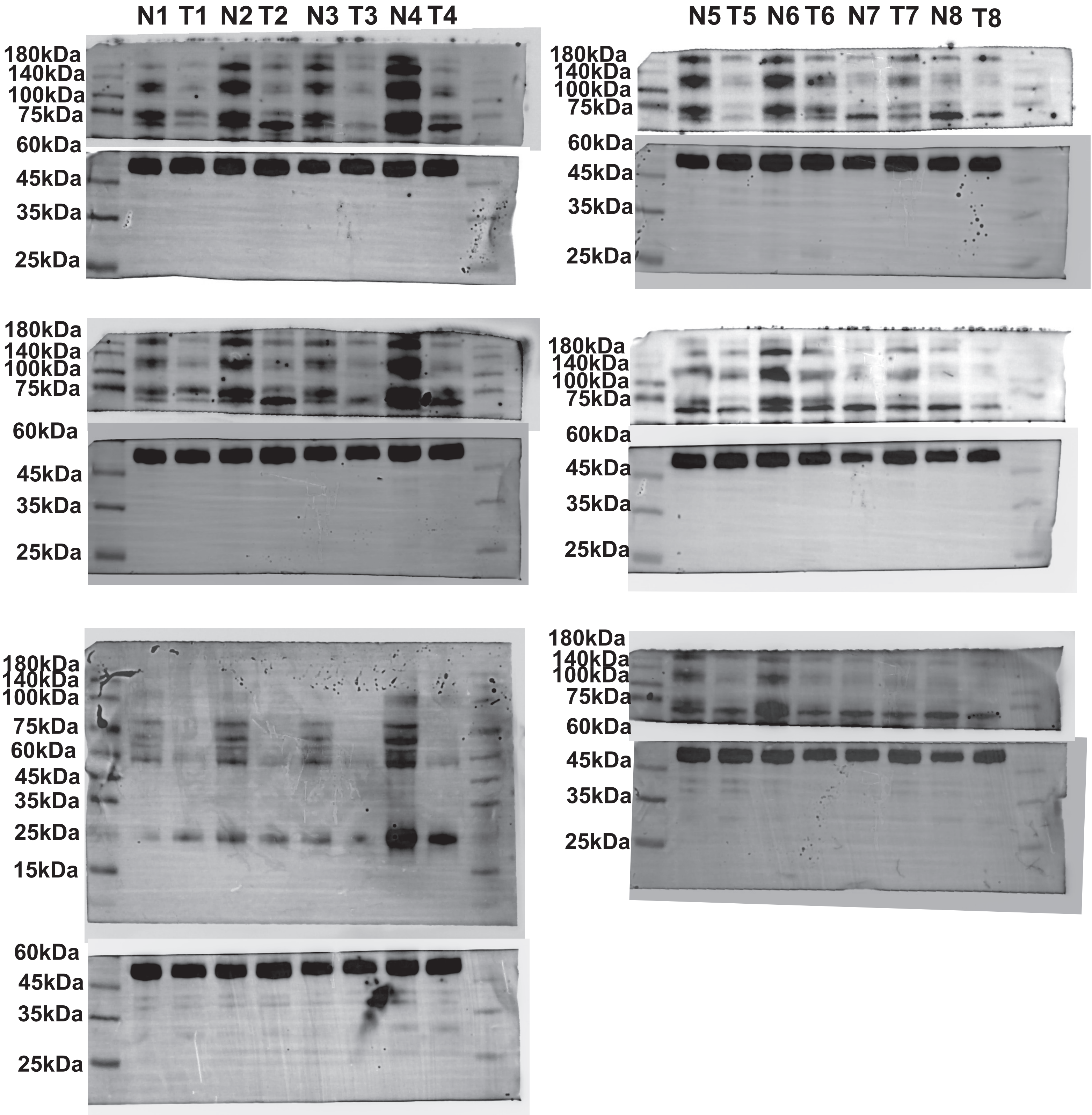

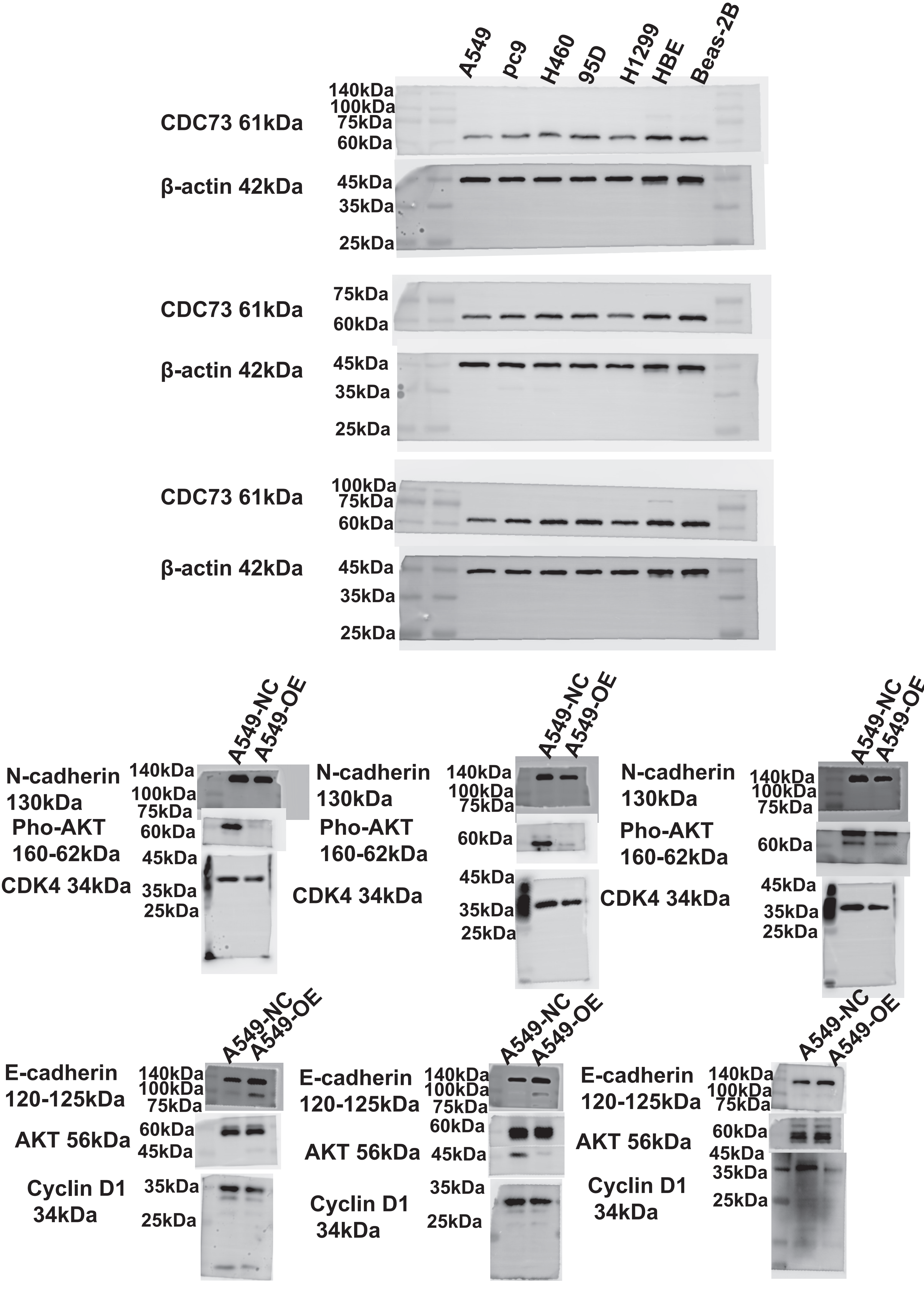

A549
pc9
H460
95D
H1299
HBE
Beas-2B
CDC73 61kDa
β-actin 42kDa
140kDa
100kDa
75kDa
60kDa
45kDa
35kDa
25kDa
A549-NC
A549-OE
N-cadherin 130kDa
Pho-AKT 160-62kDa
CDK4 34kDa
E-cadherin 120-125kDa
AKT 56kDa
Cyclin D1 34kDa

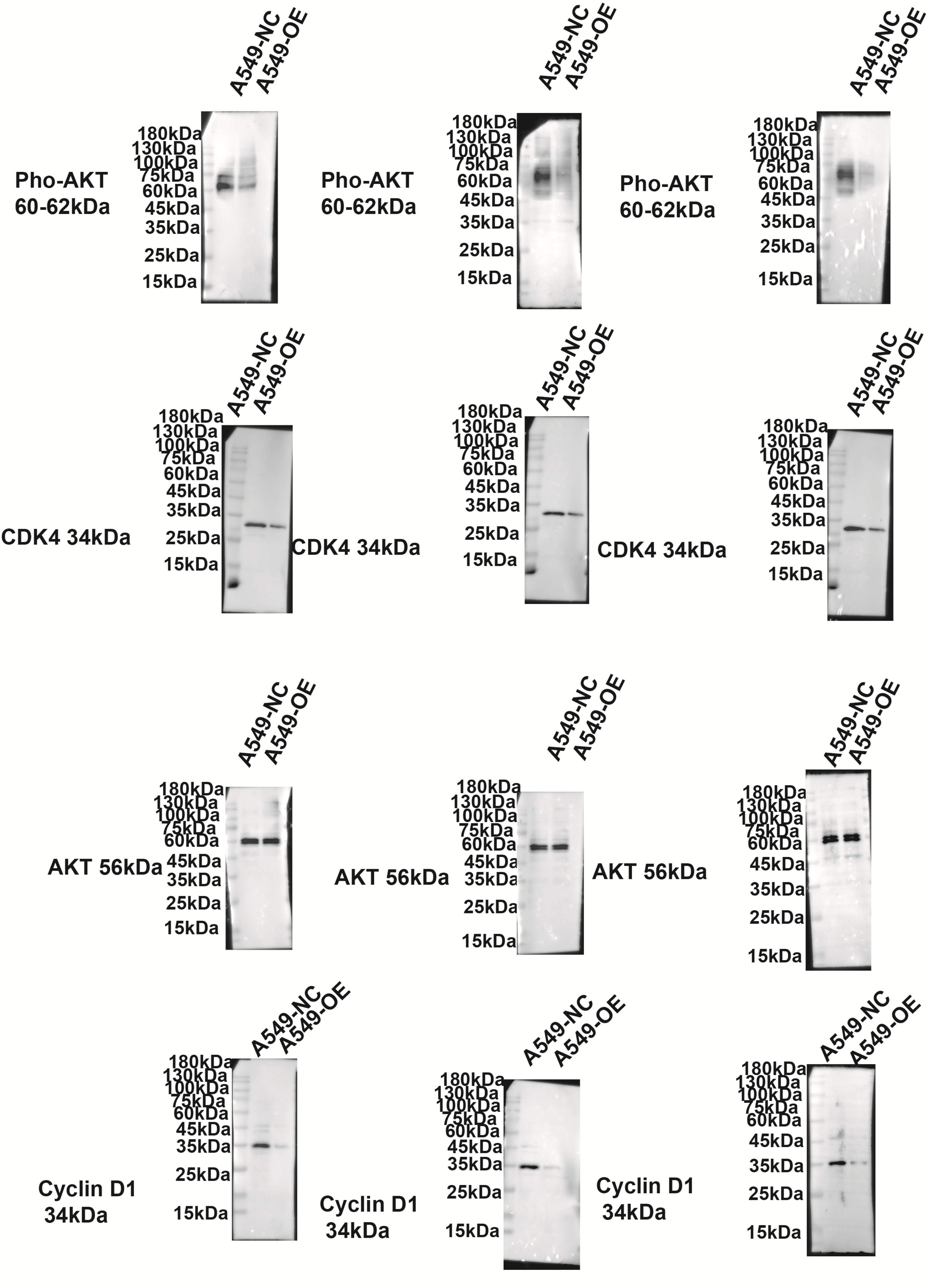
Pho-AKT 60-62kDa
A549-NC
A549-OE
180kDa
130kDa
100kDa
75kDa
60kDa
45kDa
35kDa
25kDa
15kDa
CDK4 34kDa
AKT 56kDa
Cyclin D1 34kDa

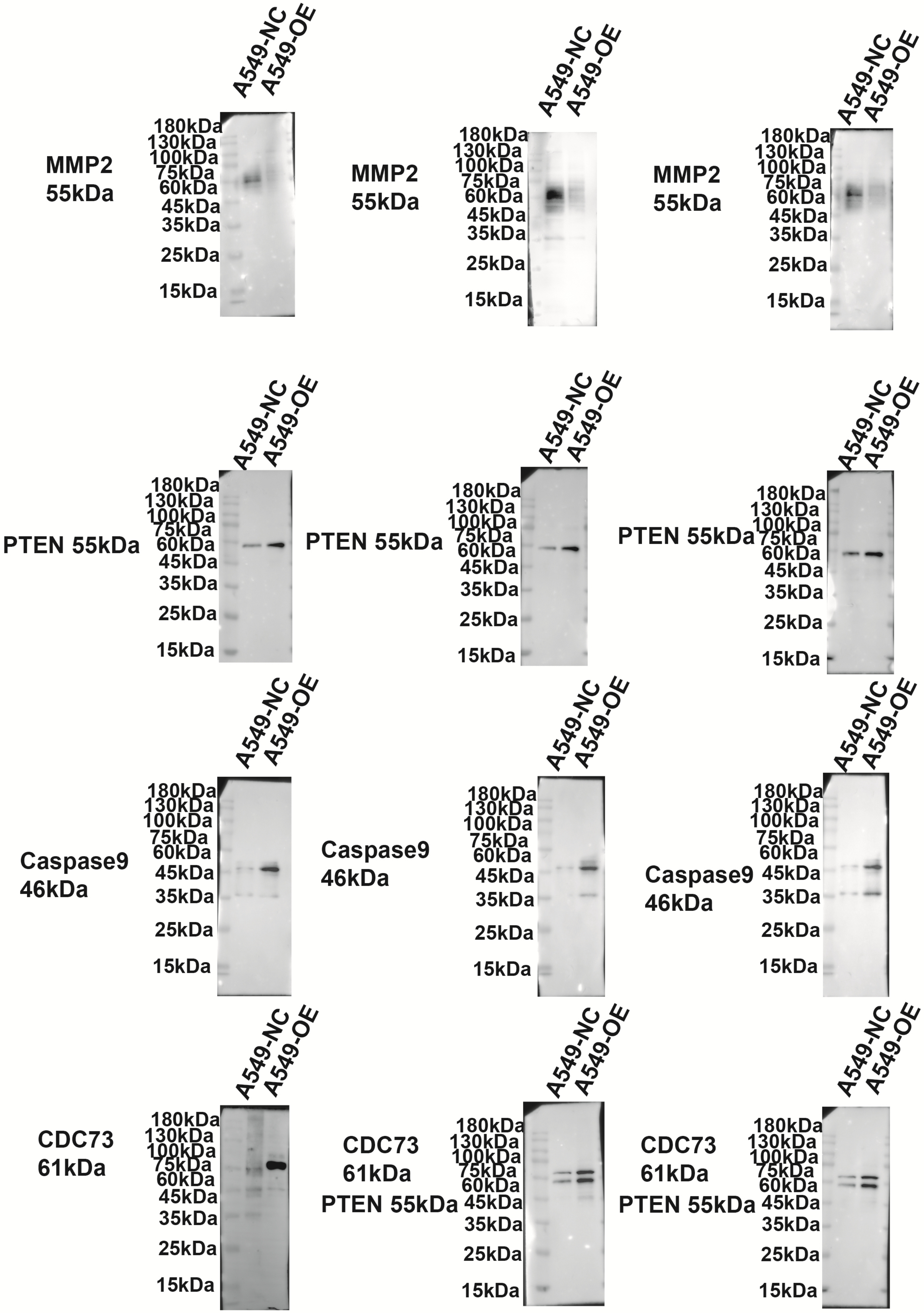

A549-NC
A549-OE
MMP2 55kDa
180kDa
130kDa
100kDa
75kDa
60kDa
45kDa
35kDa
25kDa
15kDa
A549-NC
A549-OE
MMP2 55kDa
180kDa
130kDa
100kDa
75kDa
60kDa
45kDa
35kDa
25kDa
15kDa
A549-NC
A549-OE
MMP2 55kDa
180kDa
130kDa
100kDa
75kDa
60kDa
45kDa
35kDa
25kDa
15kDa
A549-NC
A549-OE
PTEN 55kDa
180kDa
130kDa
100kDa
75kDa
60kDa
45kDa
35kDa
25kDa
15kDa
A549-NC
A549-OE
PTEN 55kDa
180kDa
130kDa
100kDa
75kDa
60kDa
45kDa
35kDa
25kDa
15kDa
A549-NC
A549-OE
PTEN 55kDa
180kDa
130kDa
100kDa
75kDa
60kDa
45kDa
35kDa
25kDa
15kDa
A549-NC
A549-OE
Caspase9 46kDa
180kDa
130kDa
100kDa
75kDa
60kDa
45kDa
35kDa
25kDa
15kDa
A549-NC
A549-OE
Caspase9 46kDa
180kDa
130kDa
100kDa
75kDa
60kDa
45kDa
35kDa
25kDa
15kDa
A549-NC
A549-OE
Caspase9 46kDa
180kDa
130kDa
100kDa
75kDa
60kDa
45kDa
35kDa
25kDa
15kDa
A549-NC
A549-OE
CDC73 61kDa
180kDa
130kDa
100kDa
75kDa
60kDa
45kDa
35kDa
25kDa
15kDa
A549-NC
A549-OE
CDC73 61kDa
PTEN 55kDa
180kDa
130kDa
100kDa
75kDa
60kDa
45kDa
35kDa
25kDa
15kDa
A549-NC
A549-OE
CDC73 61kDa
PTEN 55kDa
180kDa
130kDa
100kDa
75kDa
60kDa
45kDa
35kDa
25kDa
15kDa

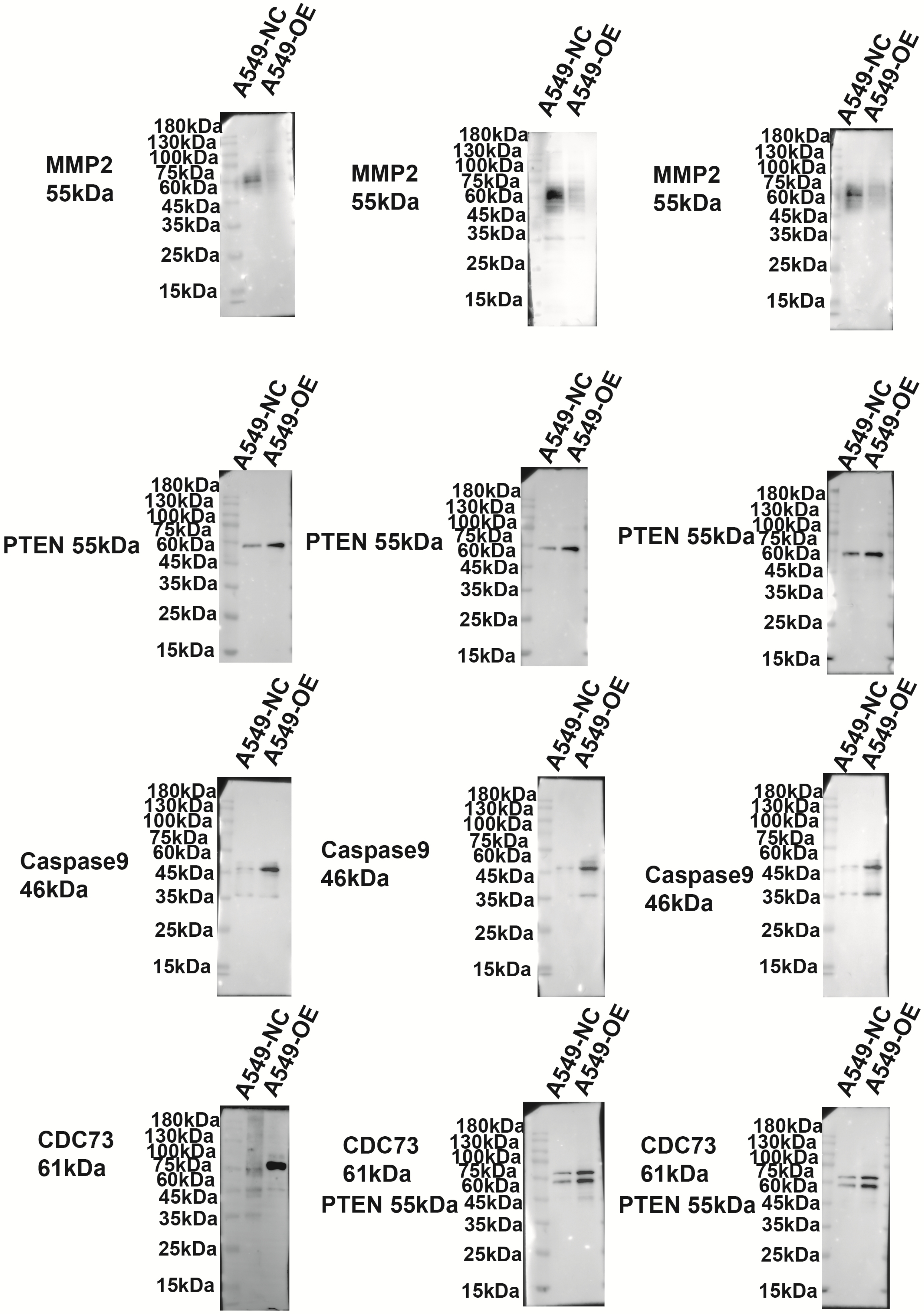
A549-NC
A549-OE
MMP2
55kDa
180kDa
130kDa
100kDa
75kDa
60kDa
45kDa
35kDa
25kDa
15kDa
A549-NC
A549-OE
MMP2
55kDa
180kDa
130kDa
100kDa
75kDa
60kDa
45kDa
35kDa
25kDa
15kDa
A549-NC
A549-OE
MMP2
55kDa
180kDa
130kDa
100kDa
75kDa
60kDa
45kDa
35kDa
25kDa
15kDa
A549-NC
A549-OE
PTEN 55kDa
180kDa
130kDa
100kDa
75kDa
60kDa
45kDa
35kDa
25kDa
15kDa
A549-NC
A549-OE
PTEN 55kDa
180kDa
130kDa
100kDa
75kDa
60kDa
45kDa
35kDa
25kDa
15kDa
A549-NC
A549-OE
PTEN 55kDa
180kDa
130kDa
100kDa
75kDa
60kDa
45kDa
35kDa
25kDa
15kDa
A549-NC
A549-OE
Caspase9
46kDa
180kDa
130kDa
100kDa
75kDa
60kDa
45kDa
35kDa
25kDa
15kDa
A549-NC
A549-OE
Caspase9
46kDa
180kDa
130kDa
100kDa
75kDa
60kDa
45kDa
35kDa
25kDa
15kDa
A549-NC
A549-OE
Caspase9
46kDa
180kDa
130kDa
100kDa
75kDa
60kDa
45kDa
35kDa
25kDa
15kDa
A549-NC
A549-OE
CDC73
61kDa
180kDa
130kDa
100kDa
75kDa
60kDa
45kDa
35kDa
25kDa
15kDa
A549-NC
A549-OE
CDC73
61kDa
PTEN 55kDa
180kDa
130kDa
100kDa
75kDa
60kDa
45kDa
35kDa
25kDa
15kDa
A549-NC
A549-OE
CDC73
61kDa
PTEN 55kDa
180kDa
130kDa
100kDa
75kDa
60kDa
45kDa
35kDa
25kDa
15kDa

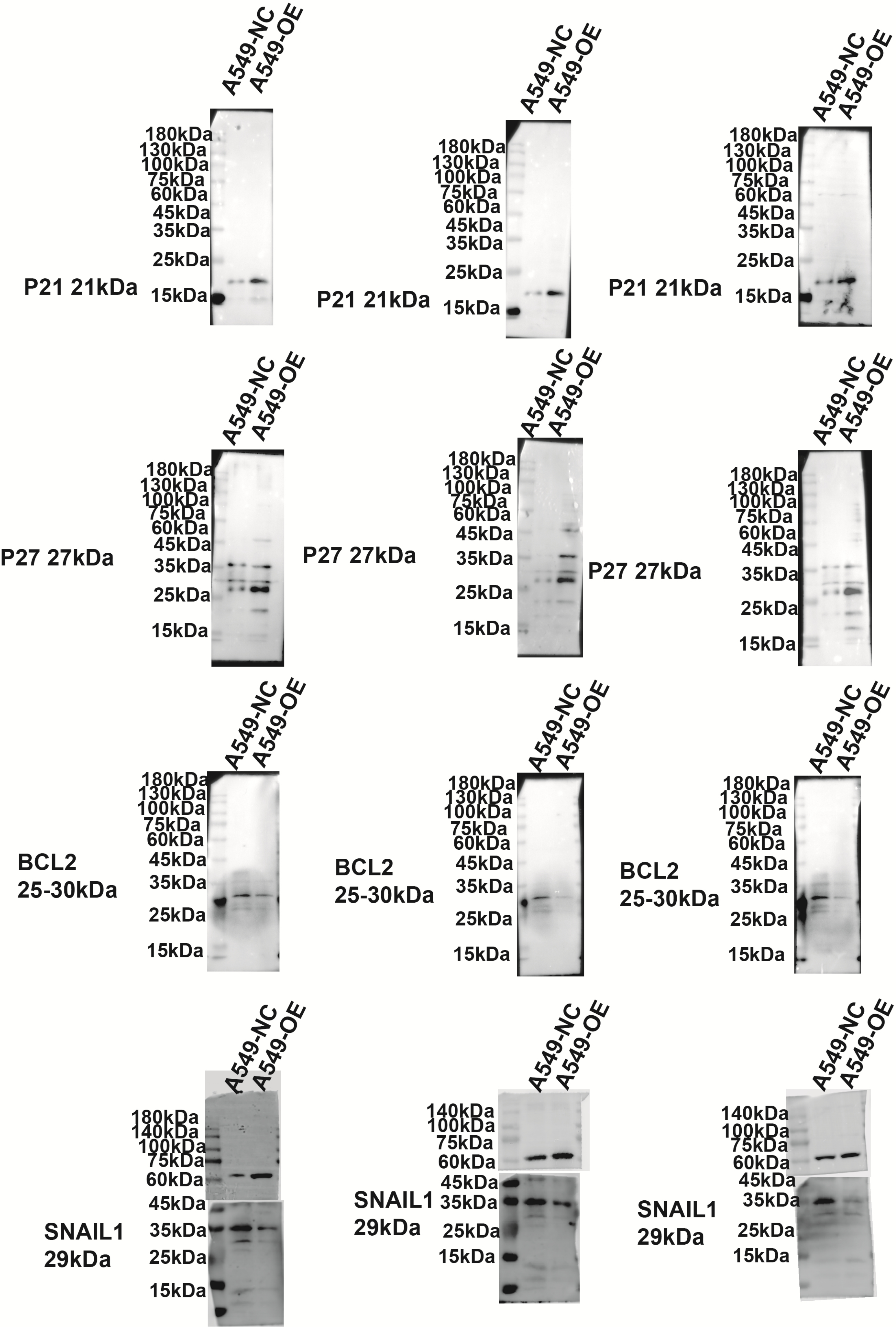
A549-NC
A549-OE
180kDa
130kDa
100kDa
75kDa
60kDa
45kDa
35kDa
25kDa
15kDa
P21 21kDa
A549-NC
A549-OE
180kDa
130kDa
100kDa
75kDa
60kDa
45kDa
35kDa
25kDa
15kDa
P21 21kDa
A549-NC
A549-OE
180kDa
130kDa
100kDa
75kDa
60kDa
45kDa
35kDa
25kDa
15kDa
P21 21kDa
A549-NC
A549-OE
180kDa
130kDa
100kDa
75kDa
60kDa
45kDa
35kDa
25kDa
15kDa
P27 27kDa
A549-NC
A549-OE
180kDa
130kDa
100kDa
75kDa
60kDa
45kDa
35kDa
25kDa
15kDa
P27 27kDa
A549-NC
A549-OE
180kDa
130kDa
100kDa
75kDa
60kDa
45kDa
35kDa
25kDa
15kDa
P27 27kDa
A549-NC
A549-OE
180kDa
130kDa
100kDa
75kDa
60kDa
45kDa
35kDa
25kDa
15kDa
BCL2
25-30kDa
A549-NC
A549-OE
180kDa
130kDa
100kDa
75kDa
60kDa
45kDa
35kDa
25kDa
15kDa
BCL2
25-30kDa
A549-NC
A549-OE
180kDa
130kDa
100kDa
75kDa
60kDa
45kDa
35kDa
25kDa
15kDa
BCL2
25-30kDa
A549-NC
A549-OE
180kDa
140kDa
100kDa
75kDa
60kDa
45kDa
35kDa
25kDa
15kDa
SNAIL1
29kDa
A549-NC
A549-OE
140kDa
100kDa
75kDa
60kDa
45kDa
35kDa
25kDa
15kDa
SNAIL1
29kDa
A549-NC
A549-OE
140kDa
100kDa
75kDa
60kDa
45kDa
35kDa
25kDa
15kDa
SNAIL1
29kDa

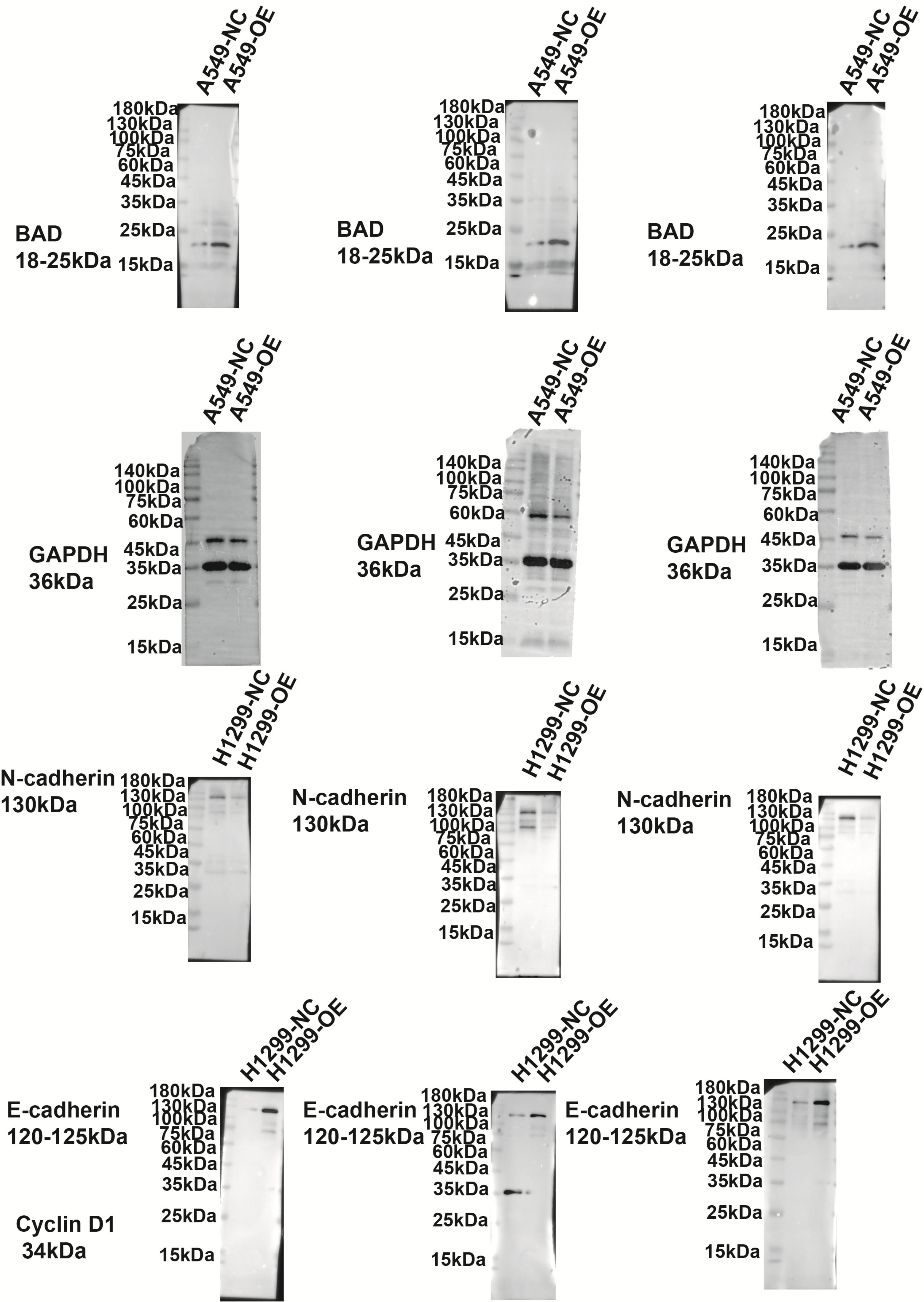

A549-NC
A549-OE
BAD
18-25kDa
180kDa
130kDa
100kDa
75kDa
60kDa
45kDa
35kDa
25kDa
15kDa
A549-NC
A549-OE
BAD
18-25kDa
180kDa
130kDa
100kDa
75kDa
60kDa
45kDa
35kDa
25kDa
15kDa
A549-NC
A549-OE
BAD
18-25kDa
180kDa
130kDa
100kDa
75kDa
60kDa
45kDa
35kDa
25kDa
15kDa
A549-NC
A549-OE
GAPDH
36kDa
140kDa
100kDa
75kDa
60kDa
45kDa
35kDa
25kDa
15kDa
A549-NC
A549-OE
GAPDH
36kDa
140kDa
100kDa
75kDa
60kDa
45kDa
35kDa
25kDa
15kDa
A549-NC
A549-OE
GAPDH
36kDa
140kDa
100kDa
75kDa
60kDa
45kDa
35kDa
25kDa
15kDa
H1299-NC
H1299-OE
N-cadherin
130kDa
180kDa
130kDa
100kDa
75kDa
60kDa
45kDa
35kDa
25kDa
15kDa
H1299-NC
H1299-OE
N-cadherin
130kDa
180kDa
130kDa
100kDa
75kDa
60kDa
45kDa
35kDa
25kDa
15kDa
H1299-NC
H1299-OE
N-cadherin
130kDa
180kDa
130kDa
100kDa
75kDa
60kDa
45kDa
35kDa
25kDa
15kDa
H1299-NC
H1299-OE
E-cadherin
120-125kDa
Cyclin D1
34kDa
180kDa
130kDa
100kDa
75kDa
60kDa
45kDa
35kDa
25kDa
15kDa
H1299-NC
H1299-OE
E-cadherin
120-125kDa
180kDa
130kDa
100kDa
75kDa
60kDa
45kDa
35kDa
25kDa
15kDa
H1299-NC
H1299-OE
E-cadherin
120-125kDa
180kDa
130kDa
100kDa
75kDa
60kDa
45kDa
35kDa
25kDa
15kDa

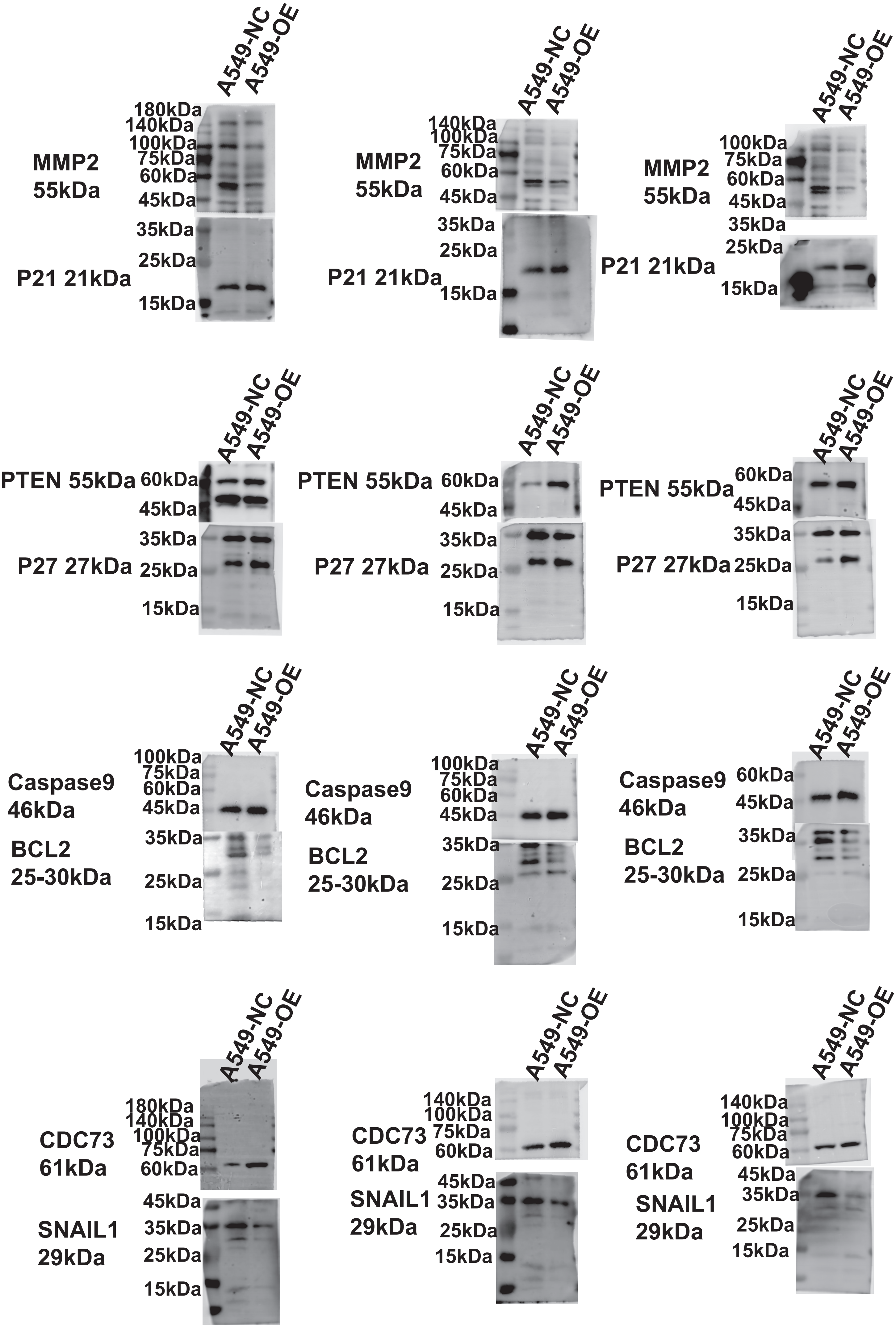

A549-NC
A549-OE
MMP2 55kDa
180kDa
140kDa
100kDa
75kDa
60kDa
45kDa
P21 21kDa
35kDa
25kDa
15kDa
A549-NC
A549-OE
MMP2 55kDa
140kDa
100kDa
75kDa
60kDa
45kDa
P21 21kDa
35kDa
25kDa
15kDa
A549-NC
A549-OE
MMP2 55kDa
100kDa
75kDa
60kDa
45kDa
35kDa
P21 21kDa
25kDa
15kDa
A549-NC
A549-OE
PTEN 55kDa
60kDa
45kDa
P27 27kDa
35kDa
25kDa
15kDa
A549-NC
A549-OE
PTEN 55kDa
60kDa
45kDa
P27 27kDa
35kDa
25kDa
15kDa
A549-NC
A549-OE
PTEN 55kDa
60kDa
45kDa
P27 27kDa
35kDa
25kDa
15kDa
A549-NC
A549-OE
Caspase9 46kDa
100kDa
75kDa
60kDa
45kDa
BCL2 25-30kDa
35kDa
25kDa
15kDa
A549-NC
A549-OE
Caspase9 46kDa
100kDa
75kDa
60kDa
45kDa
BCL2 25-30kDa
35kDa
25kDa
15kDa
A549-NC
A549-OE
Caspase9 46kDa
60kDa
45kDa
BCL2 25-30kDa
35kDa
25kDa
15kDa
A549-NC
A549-OE
CDC73 61kDa
180kDa
140kDa
100kDa
75kDa
60kDa
SNAIL1 29kDa
45kDa
35kDa
25kDa
15kDa
A549-NC
A549-OE
CDC73 61kDa
140kDa
100kDa
75kDa
60kDa
SNAIL1 29kDa
45kDa
35kDa
25kDa
15kDa
A549-NC
A549-OE
CDC73 61kDa
140kDa
100kDa
75kDa
60kDa
SNAIL1 29kDa
45kDa
35kDa
25kDa
15kDa

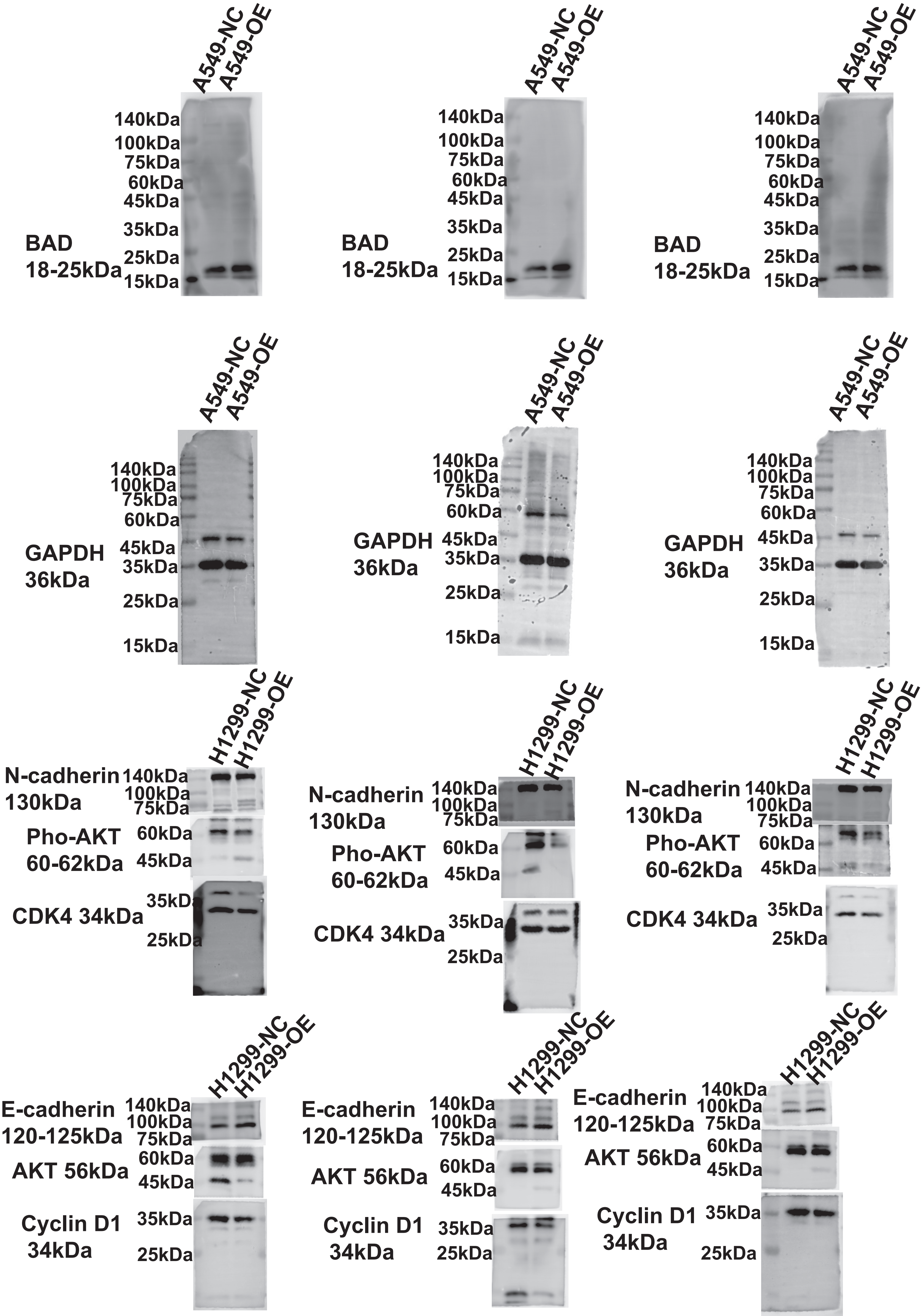

A549-NC
A549-OE
BAD
18-25kDa
140kDa
100kDa
75kDa
60kDa
45kDa
35kDa
25kDa
15kDa
A549-NC
A549-OE
BAD
18-25kDa
140kDa
100kDa
75kDa
60kDa
45kDa
35kDa
25kDa
15kDa
A549-NC
A549-OE
BAD
18-25kDa
140kDa
100kDa
75kDa
60kDa
45kDa
35kDa
25kDa
15kDa
A549-NC
A549-OE
GAPDH
36kDa
140kDa
100kDa
75kDa
60kDa
45kDa
35kDa
25kDa
15kDa
A549-NC
A549-OE
GAPDH
36kDa
140kDa
100kDa
75kDa
60kDa
45kDa
35kDa
25kDa
15kDa
A549-NC
A549-OE
GAPDH
36kDa
140kDa
100kDa
75kDa
60kDa
45kDa
35kDa
25kDa
15kDa
H1299-NC
H1299-OE
N-cadherin
130kDa
140kDa
100kDa
75kDa
Pho-AKT
60-62kDa
60kDa
45kDa
CDK4 34kDa
35kDa
25kDa
H1299-NC
H1299-OE
N-cadherin
130kDa
140kDa
100kDa
75kDa
Pho-AKT
60-62kDa
60kDa
45kDa
CDK4 34kDa
35kDa
25kDa
H1299-NC
H1299-OE
N-cadherin
130kDa
140kDa
100kDa
75kDa
Pho-AKT
60-62kDa
60kDa
45kDa
CDK4 34kDa
35kDa
25kDa
H1299-NC
H1299-OE
E-cadherin
120-125kDa
140kDa
100kDa
75kDa
AKT 56kDa
60kDa
45kDa
Cyclin D1
34kDa
35kDa
25kDa
H1299-NC
H1299-OE
E-cadherin
120-125kDa
140kDa
100kDa
75kDa
AKT 56kDa
60kDa
45kDa
Cyclin D1
34kDa
35kDa
25kDa
H1299-NC
H1299-OE
E-cadherin
120-125kDa
140kDa
100kDa
75kDa
AKT 56kDa
60kDa
45kDa
Cyclin D1
34kDa
35kDa
25kDa

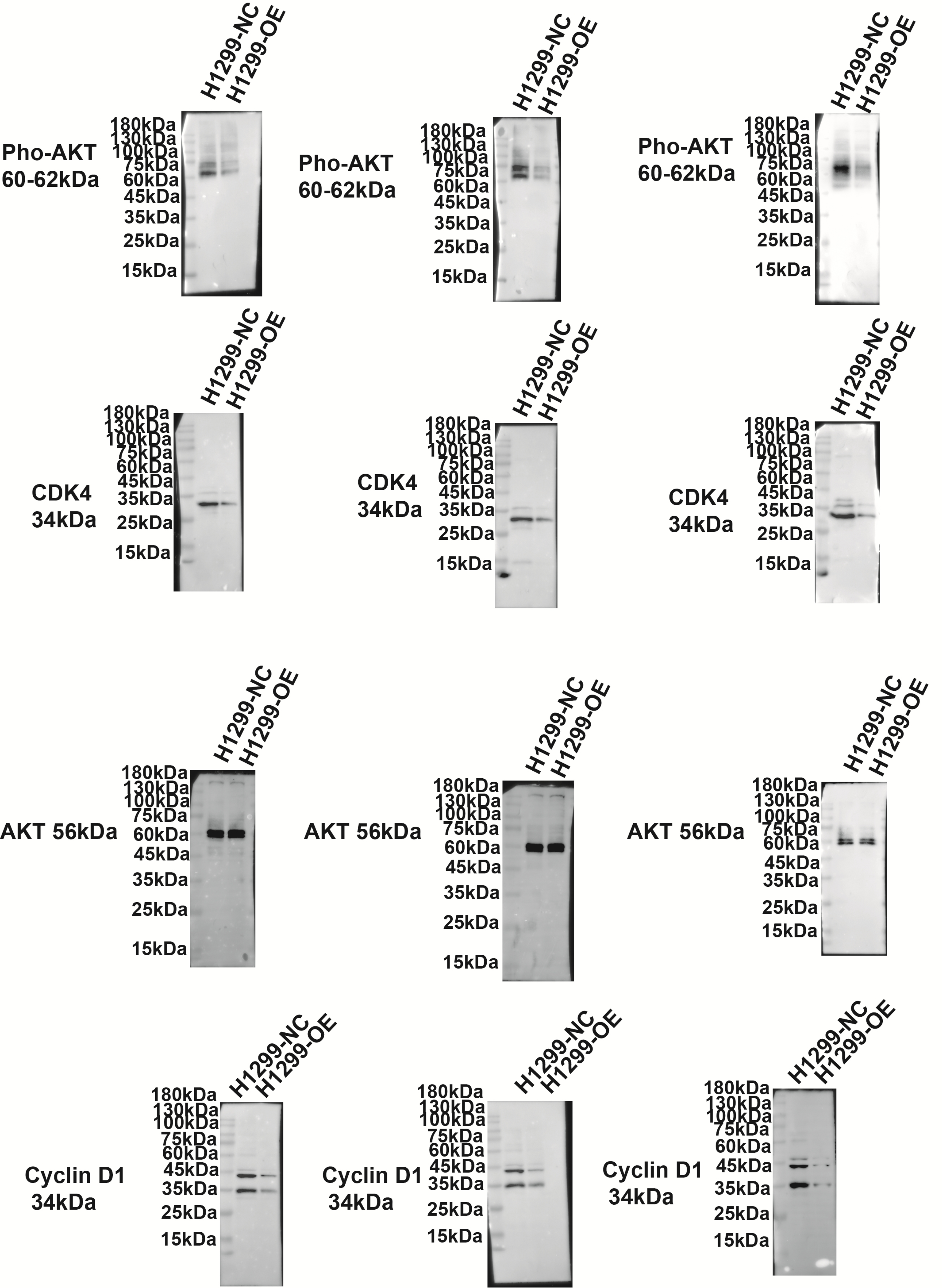
H1299-NC
H1299-OE
Pho-AKT
60-62kDa
180kDa
130kDa
100kDa
75kDa
60kDa
45kDa
35kDa
25kDa
15kDa
H1299-NC
H1299-OE
Pho-AKT
60-62kDa
180kDa
130kDa
100kDa
75kDa
60kDa
45kDa
35kDa
25kDa
15kDa
H1299-NC
H1299-OE
Pho-AKT
60-62kDa
180kDa
130kDa
100kDa
75kDa
60kDa
45kDa
35kDa
25kDa
15kDa
H1299-NC
H1299-OE
CDK4
34kDa
180kDa
130kDa
100kDa
75kDa
60kDa
45kDa
35kDa
25kDa
15kDa
H1299-NC
H1299-OE
CDK4
34kDa
180kDa
130kDa
100kDa
75kDa
60kDa
45kDa
35kDa
25kDa
15kDa
H1299-NC
H1299-OE
CDK4
34kDa
180kDa
130kDa
100kDa
75kDa
60kDa
45kDa
35kDa
25kDa
15kDa
H1299-NC
H1299-OE
AKT 56kDa
180kDa
130kDa
100kDa
75kDa
60kDa
45kDa
35kDa
25kDa
15kDa
H1299-NC
H1299-OE
AKT 56kDa
180kDa
130kDa
100kDa
75kDa
60kDa
45kDa
35kDa
25kDa
15kDa
H1299-NC
H1299-OE
AKT 56kDa
180kDa
130kDa
100kDa
75kDa
60kDa
45kDa
35kDa
25kDa
15kDa
H1299-NC
H1299-OE
Cyclin D1
34kDa
180kDa
130kDa
100kDa
75kDa
60kDa
45kDa
35kDa
25kDa
15kDa
H1299-NC
H1299-OE
Cyclin D1
34kDa
180kDa
130kDa
100kDa
75kDa
60kDa
45kDa
35kDa
25kDa
15kDa
H1299-NC
H1299-OE
Cyclin D1
34kDa
180kDa
130kDa
100kDa
75kDa
60kDa
45kDa
35kDa
25kDa
15kDa

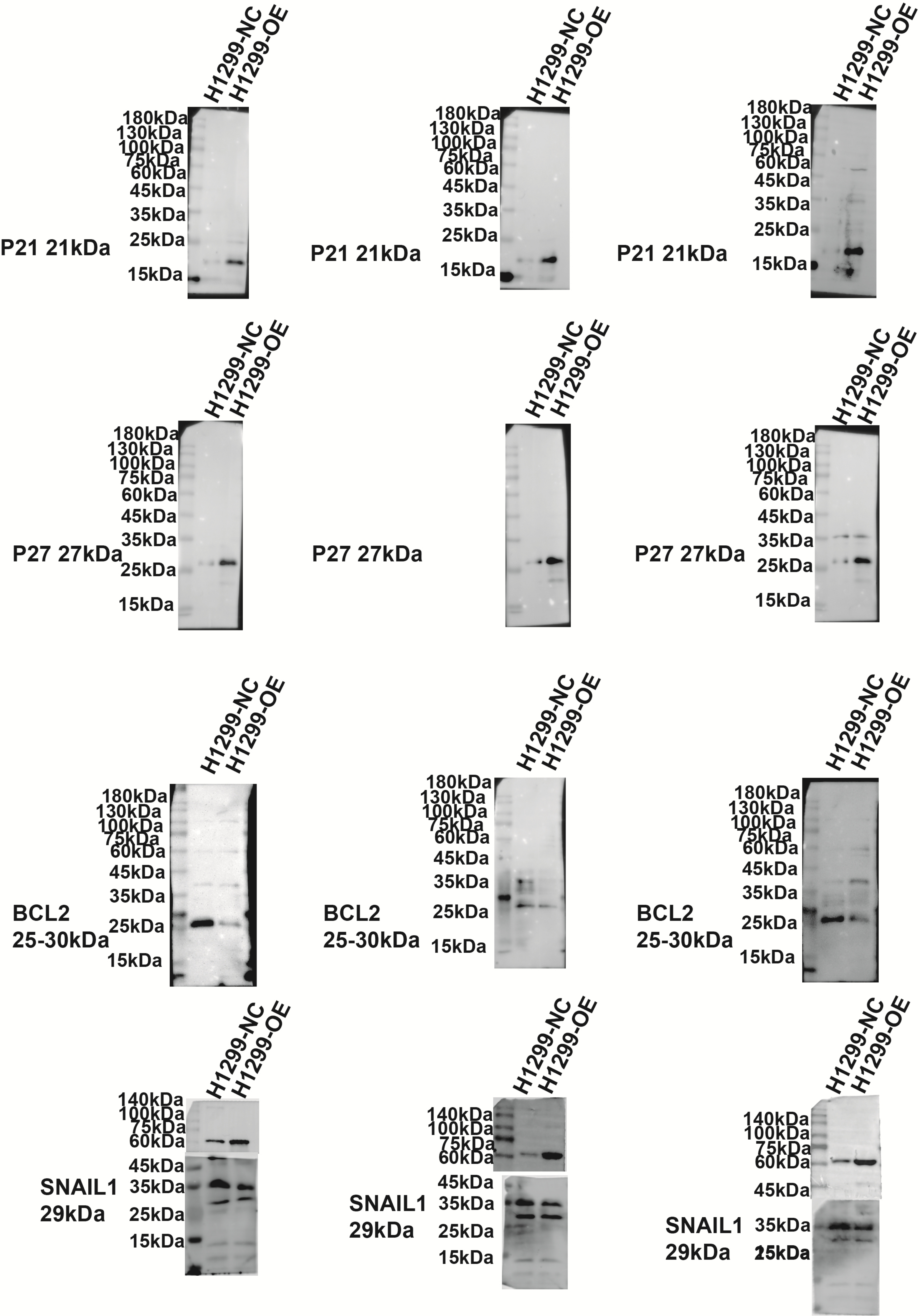
H1299-NC
H1299-OE
180kDa
130kDa
100kDa
75kDa
60kDa
45kDa
35kDa
25kDa
15kDa
P21 21kDa
H1299-NC
H1299-OE
180kDa
130kDa
100kDa
75kDa
60kDa
45kDa
35kDa
25kDa
15kDa
P21 21kDa
H1299-NC
H1299-OE
180kDa
130kDa
100kDa
75kDa
60kDa
45kDa
35kDa
25kDa
15kDa
P21 21kDa
H1299-NC
H1299-OE
180kDa
130kDa
100kDa
75kDa
60kDa
45kDa
35kDa
25kDa
15kDa
P27 27kDa
H1299-NC
H1299-OE
P27 27kDa
H1299-NC
H1299-OE
180kDa
130kDa
100kDa
75kDa
60kDa
45kDa
35kDa
25kDa
15kDa
P27 27kDa
H1299-NC
H1299-OE
180kDa
130kDa
100kDa
75kDa
60kDa
45kDa
35kDa
25kDa
15kDa
BCL2
25-30kDa
H1299-NC
H1299-OE
180kDa
130kDa
100kDa
75kDa
60kDa
45kDa
35kDa
25kDa
15kDa
BCL2
25-30kDa
H1299-NC
H1299-OE
180kDa
130kDa
100kDa
75kDa
60kDa
45kDa
35kDa
25kDa
15kDa
BCL2
25-30kDa
H1299-NC
H1299-OE
140kDa
100kDa
75kDa
60kDa
45kDa
35kDa
25kDa
15kDa
SNAIL1
29kDa
H1299-NC
H1299-OE
140kDa
100kDa
75kDa
60kDa
45kDa
35kDa
25kDa
15kDa
SNAIL1
29kDa
H1299-NC
H1299-OE
140kDa
100kDa
75kDa
60kDa
45kDa
35kDa
25kDa
SNAIL1
29kDa

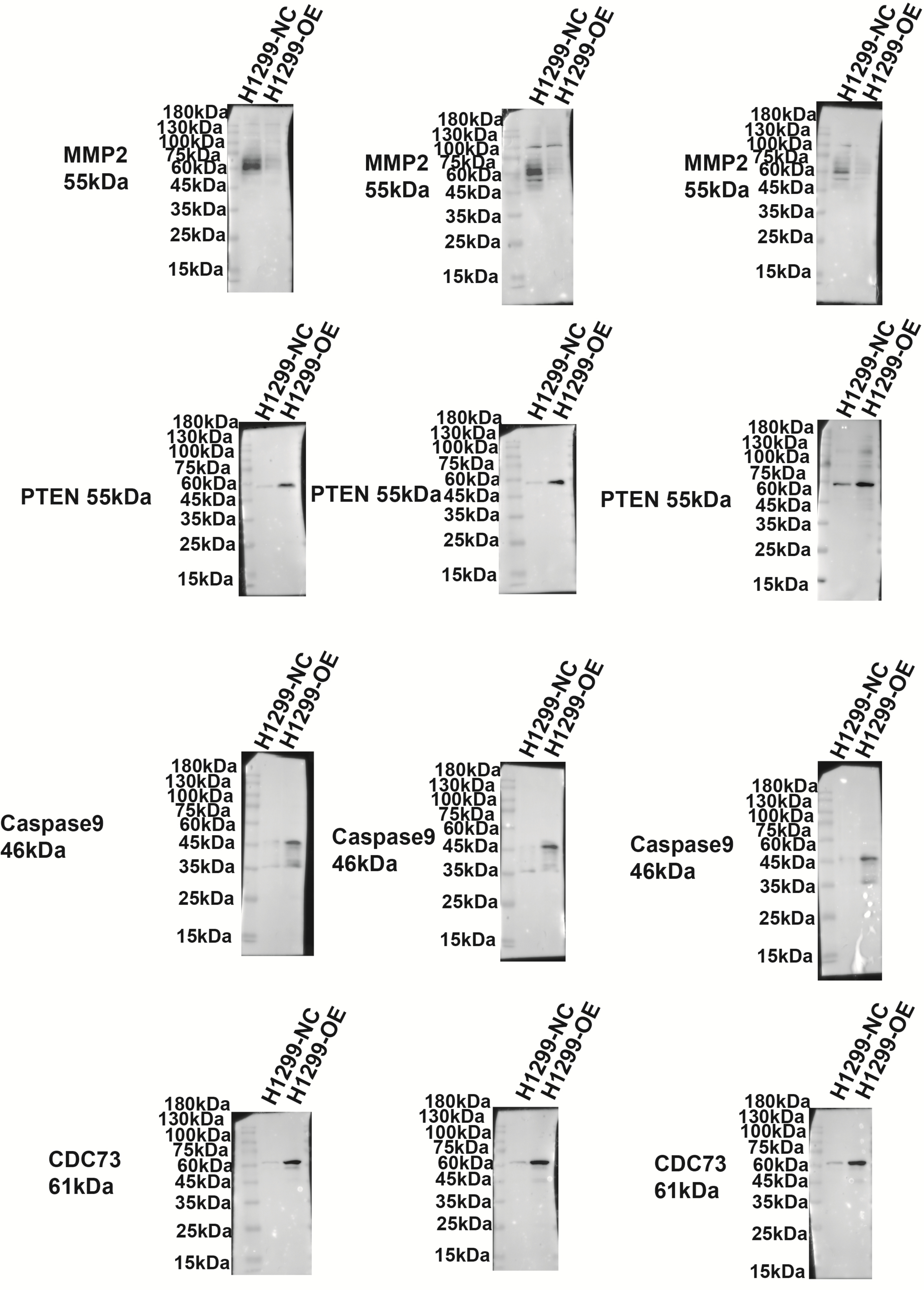
MMP2 55kDa
H1299-NC
H1299-OE
180kDa
130kDa
100kDa
75kDa
60kDa
45kDa
35kDa
25kDa
15kDa
PTEN 55kDa
Caspase9 46kDa
CDC73 61kDa

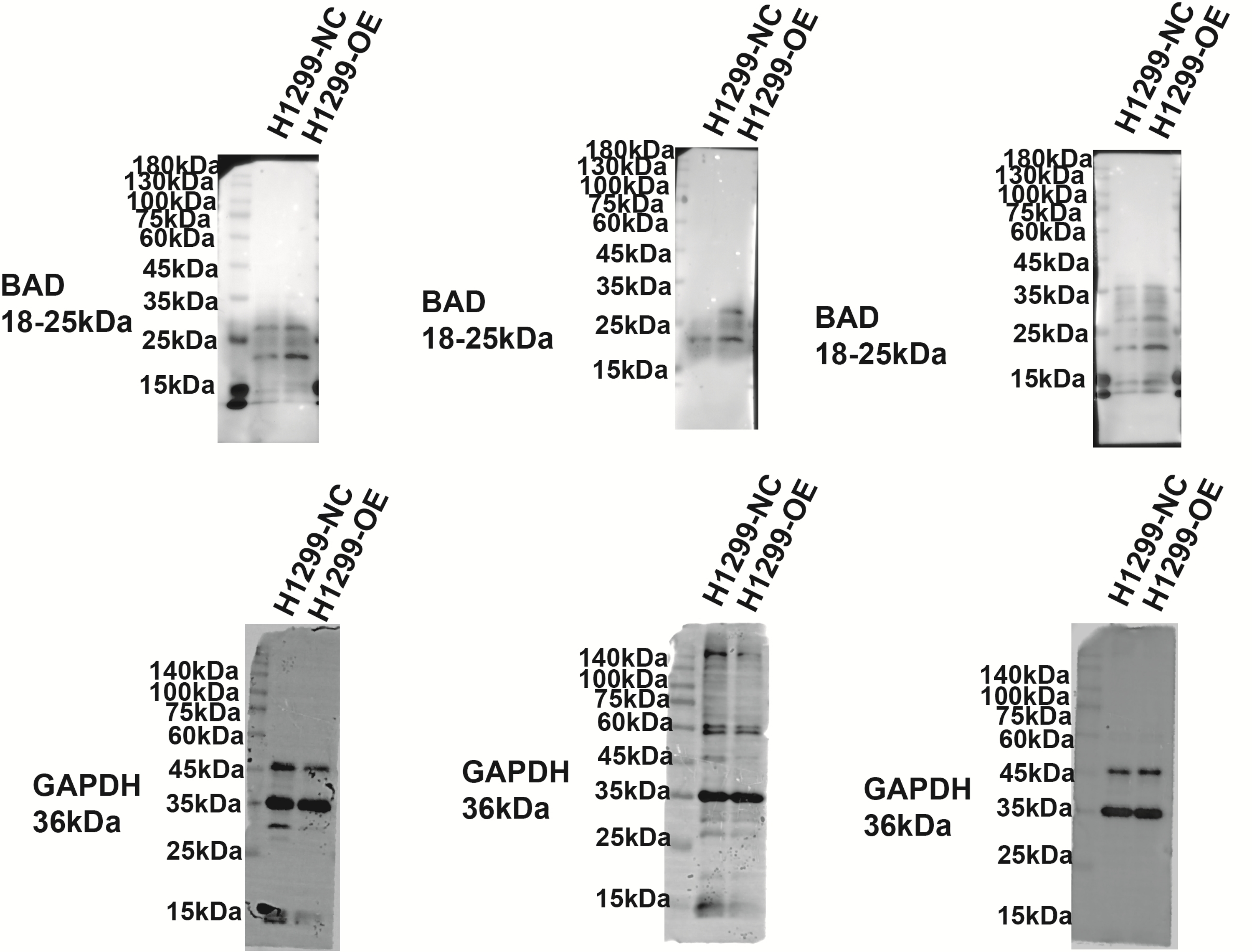
H1299-NC
H1299-OE
BAD
18-25kDa
180kDa
130kDa
100kDa
75kDa
60kDa
45kDa
35kDa
25kDa
15kDa
GAPDH
36kDa
140kDa
100kDa
75kDa
60kDa
45kDa
35kDa
25kDa
15kDa

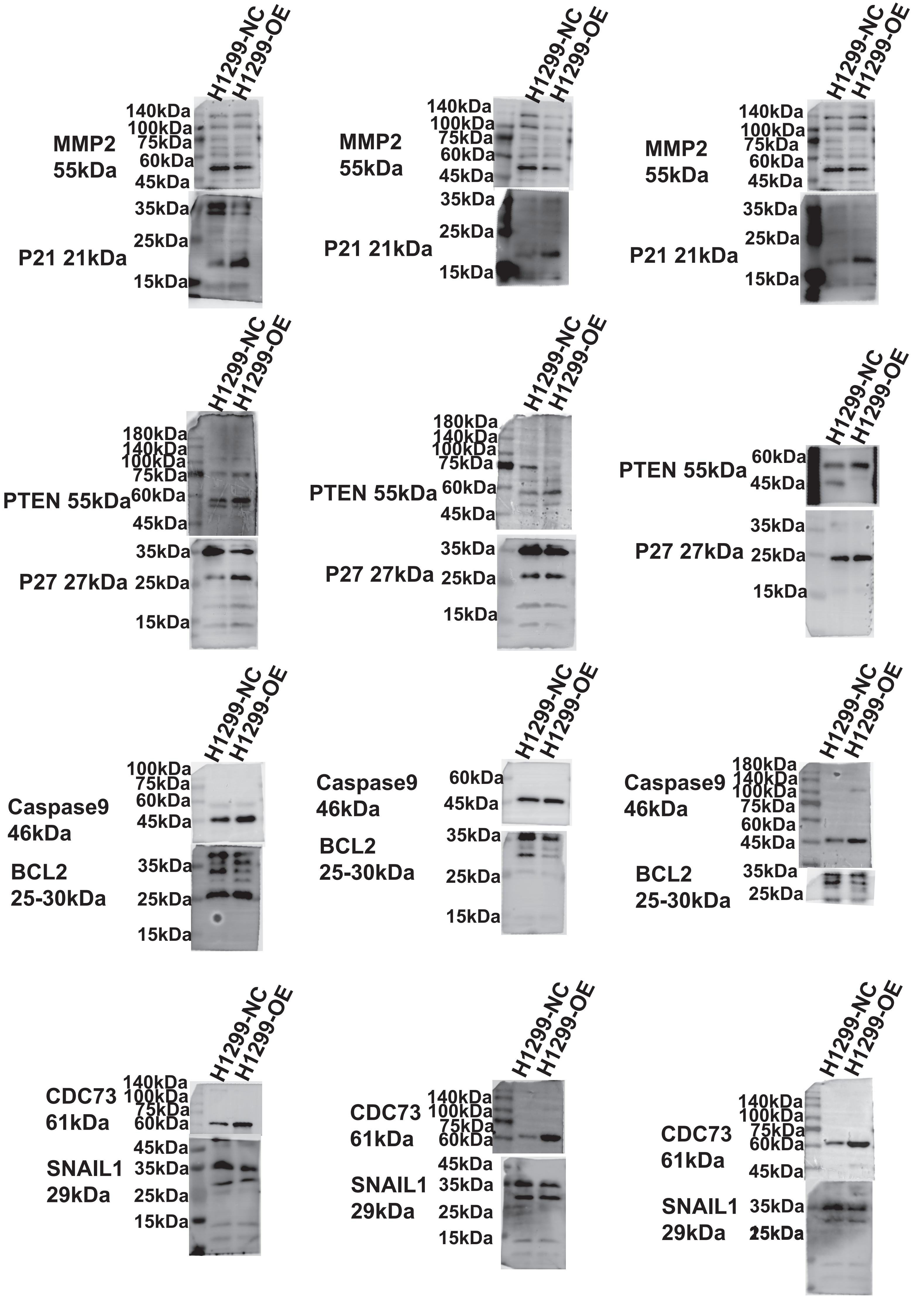

H1299-NC
H1299-OE
MMP2 55kDa
P21 21kDa
PTEN 55kDa
P27 27kDa
Caspase9 46kDa
BCL2 25-30kDa
CDC73 61kDa
SNAIL1 29kDa
180kDa
140kDa
100kDa
75kDa
60kDa
45kDa
35kDa
25kDa
15kDa

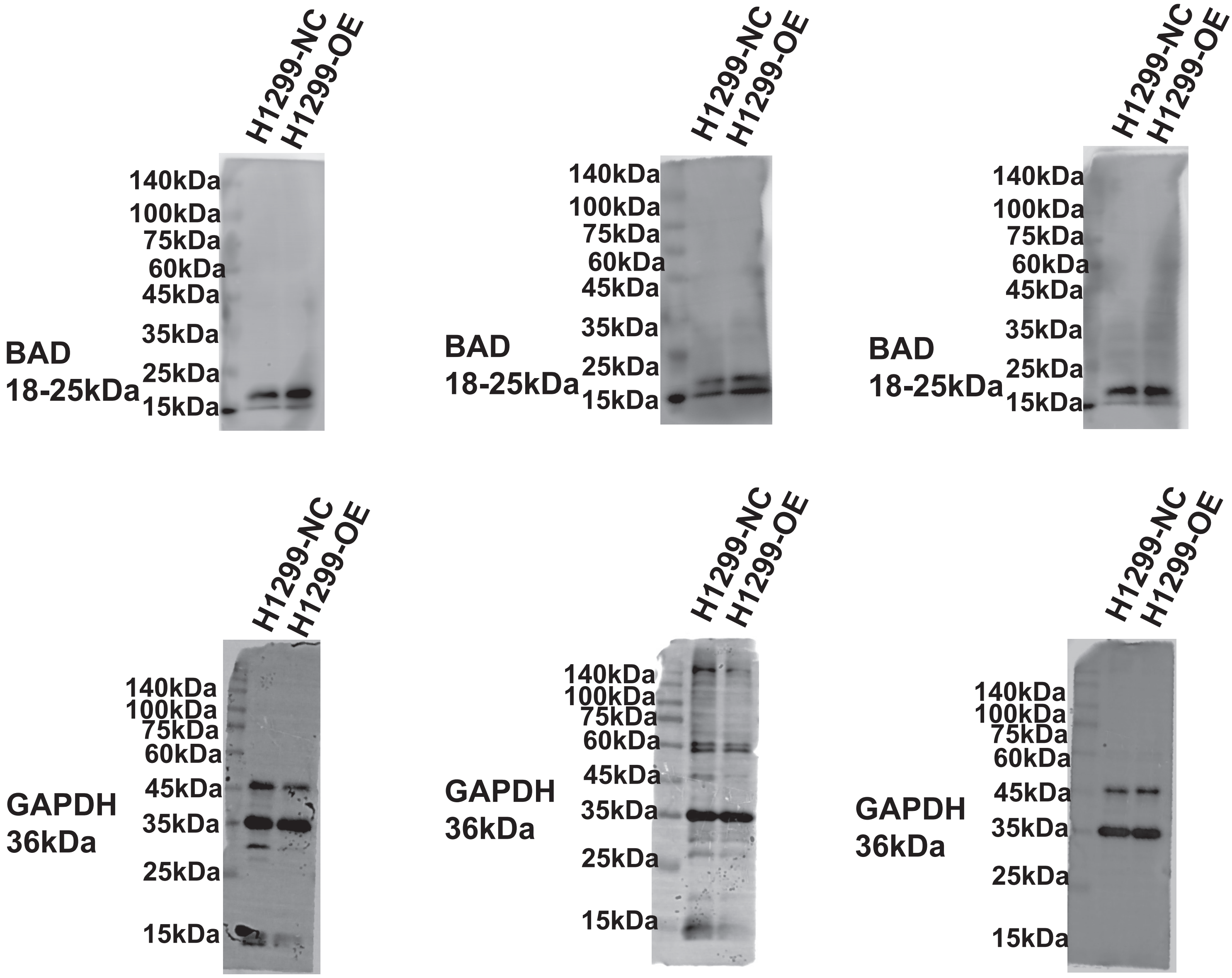
H1299-NC
H1299-OE
BAD
18-25kDa
140kDa
100kDa
75kDa
60kDa
45kDa
35kDa
25kDa
15kDa
H1299-NC
H1299-OE
BAD
18-25kDa
140kDa
100kDa
75kDa
60kDa
45kDa
35kDa
25kDa
15kDa
H1299-NC
H1299-OE
BAD
18-25kDa
140kDa
100kDa
75kDa
60kDa
45kDa
35kDa
25kDa
15kDa
H1299-NC
H1299-OE
GAPDH
36kDa
140kDa
100kDa
75kDa
60kDa
45kDa
35kDa
25kDa
15kDa
H1299-NC
H1299-OE
GAPDH
36kDa
140kDa
100kDa
75kDa
60kDa
45kDa
35kDa
25kDa
15kDa
H1299-NC
H1299-OE
GAPDH
36kDa
140kDa
100kDa
75kDa
60kDa
45kDa
35kDa
25kDa
15kDa

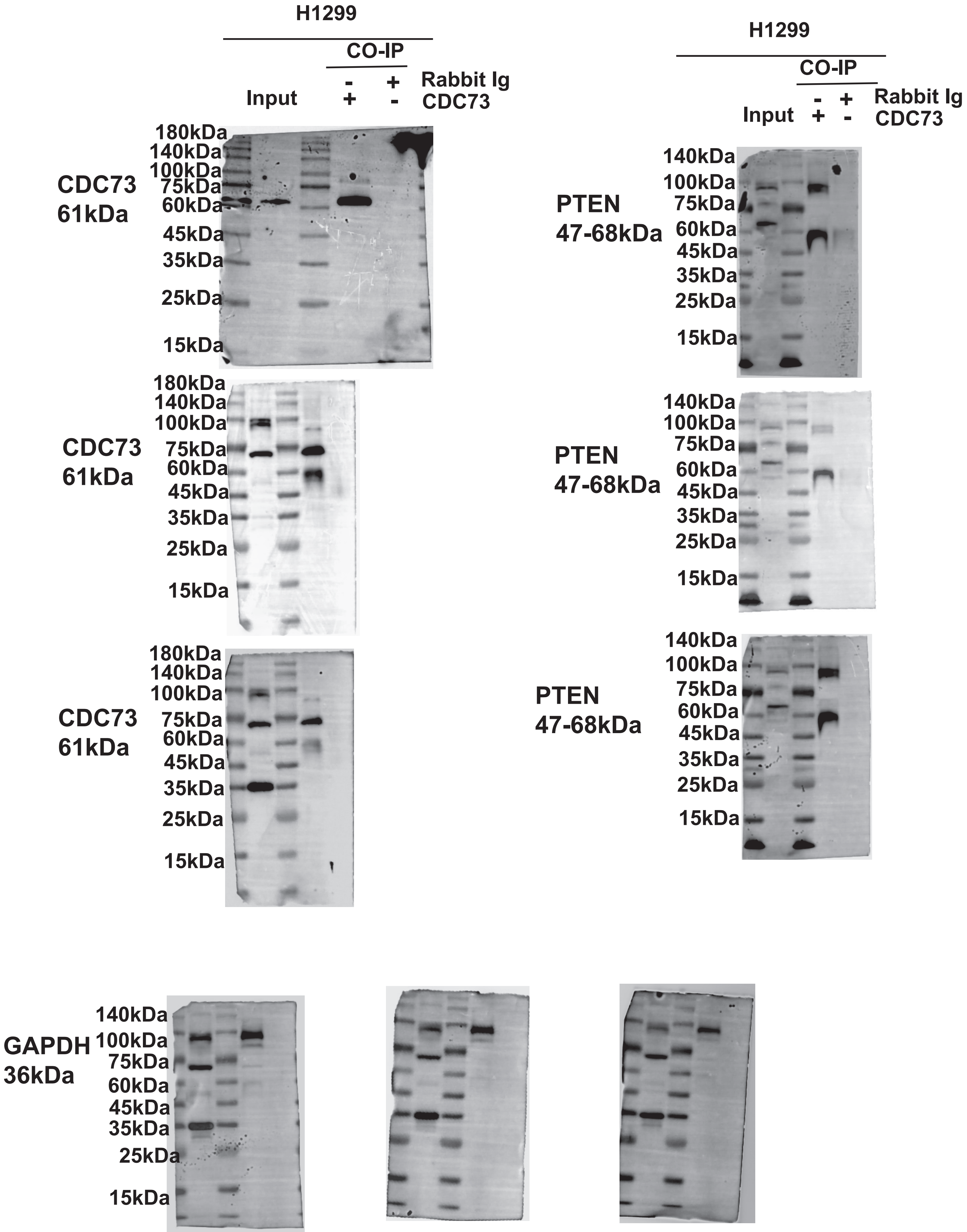
H1299
CO-IP
Input
- + Rabbit Ig
+ - CDC73
CDC73
61kDa
180kDa
140kDa
100kDa
75kDa
60kDa
45kDa
35kDa
25kDa
15kDa
PTEN
47-68kDa
GAPDH
36kDa

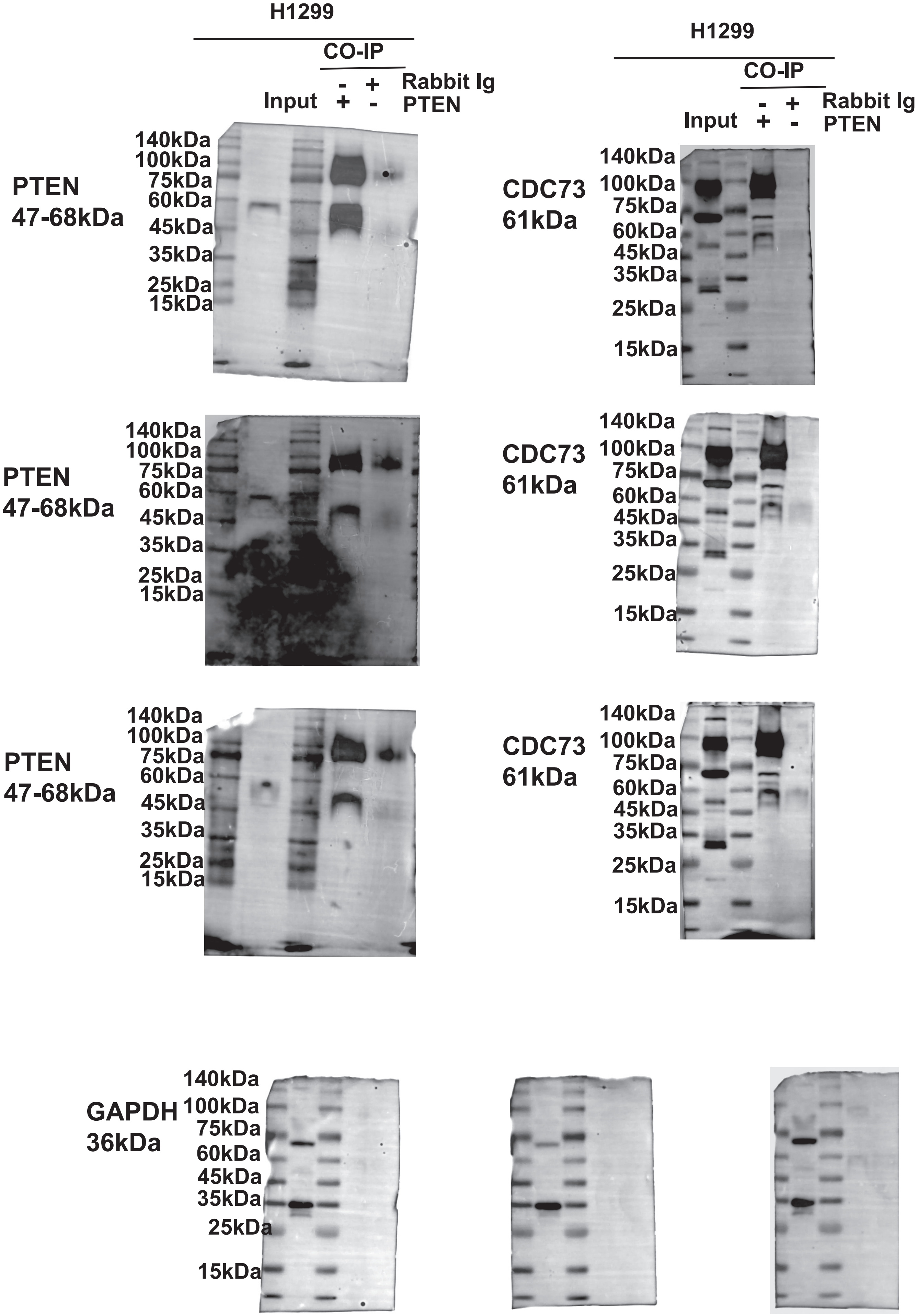

H1299
CO-IP
- + Rabbit Ig
Input + - PTEN
PTEN
47-68kDa
CDC73
61kDa
GAPDH
36kDa
140kDa
100kDa
75kDa
60kDa
45kDa
35kDa
25kDa
15kDa

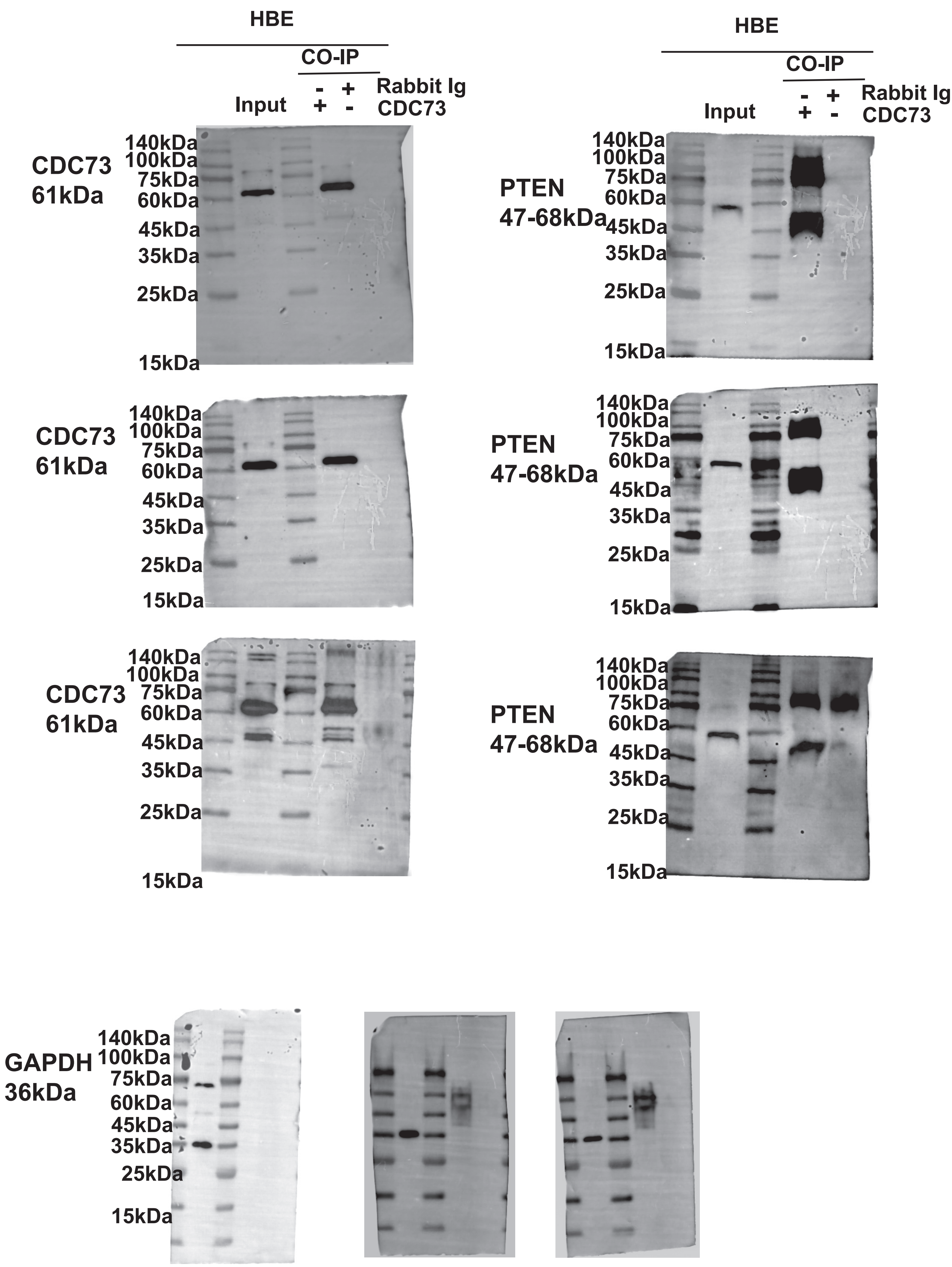

HBE
CO-IP
Input
- + Rabbit Ig
+ - CDC73
CDC73 61kDa
PTEN 47-68kDa
GAPDH 36kDa
140kDa
100kDa
75kDa
60kDa
45kDa
35kDa
25kDa
15kDa

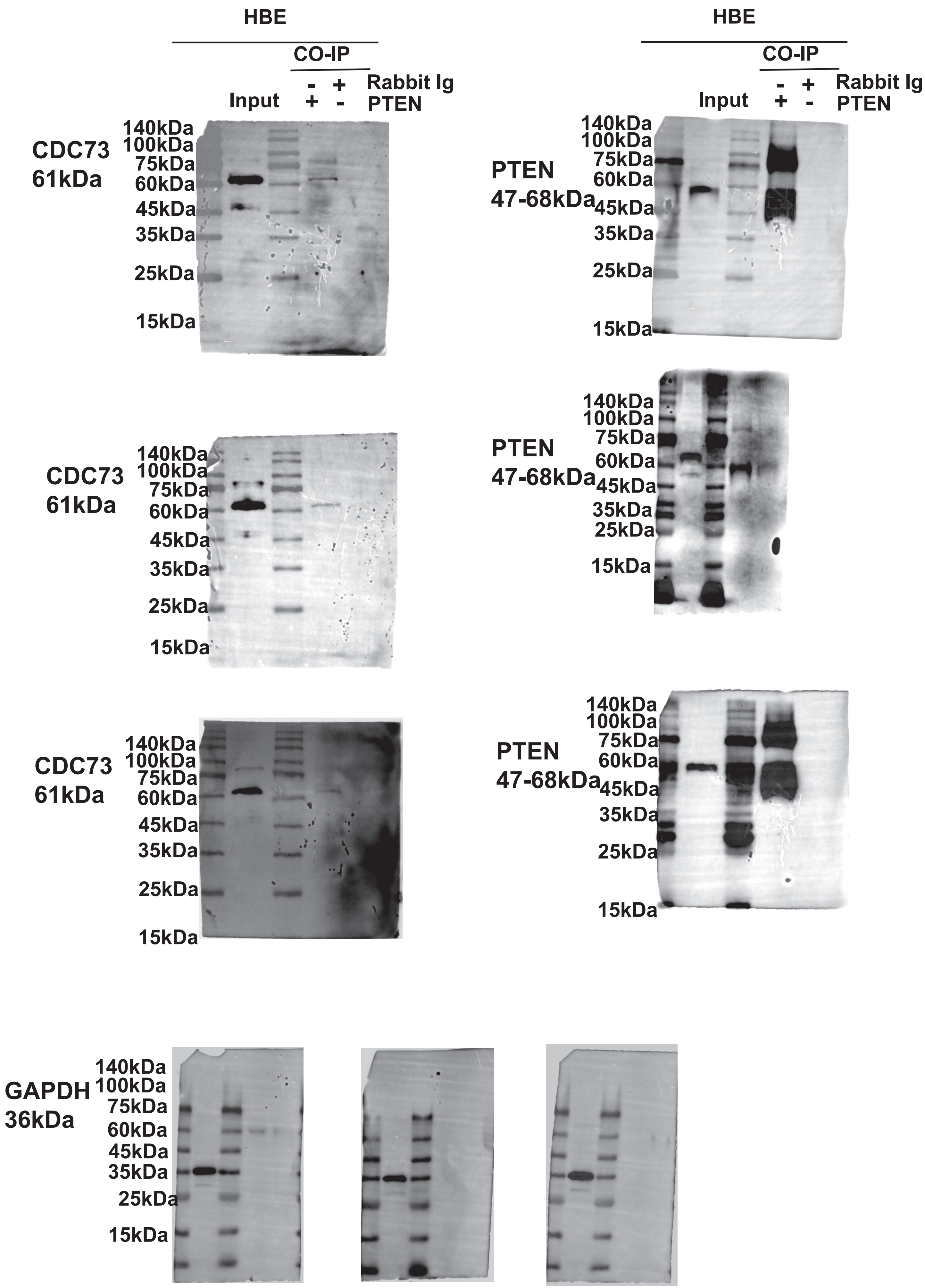
HBE
CO-IP
Input
- + Rabbit Ig
+ - PTEN
CDC73
61kDa
PTEN
47-68kDa
GAPDH
36kDa
140kDa
100kDa
75kDa
60kDa
45kDa
35kDa
25kDa
15kDa

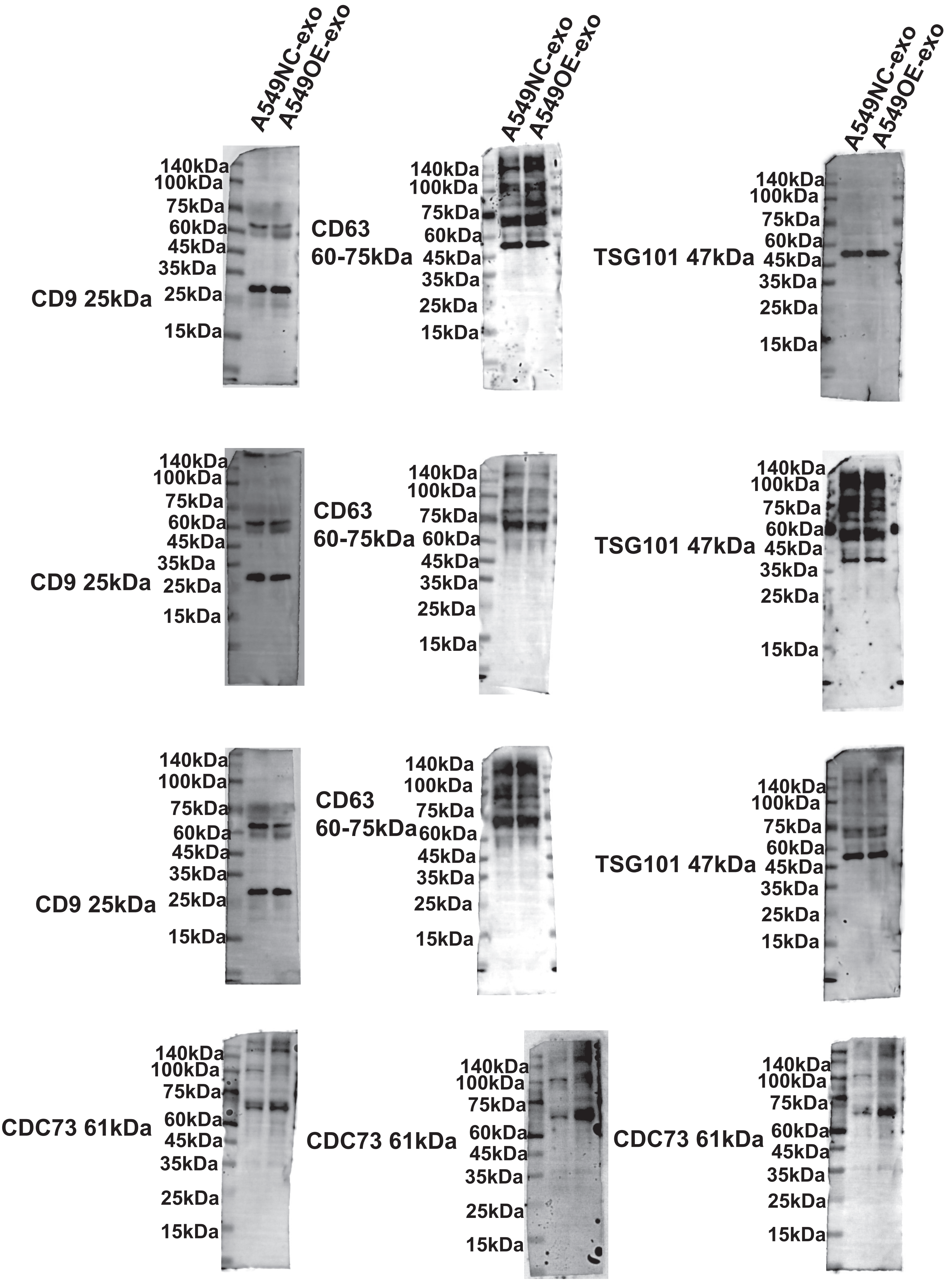
A549NC-exo
A549OE-exo
140kDa
100kDa
75kDa
60kDa
45kDa
35kDa
25kDa
15kDa
CD9 25kDa
CD63 60-75kDa
TSG101 47kDa
CD9 25kDa
CD63 60-75kDa
TSG101 47kDa
CD9 25kDa
CD63 60-75kDa
TSG101 47kDa
CDC73 61kDa
CDC73 61kDa
CDC73 61kDa

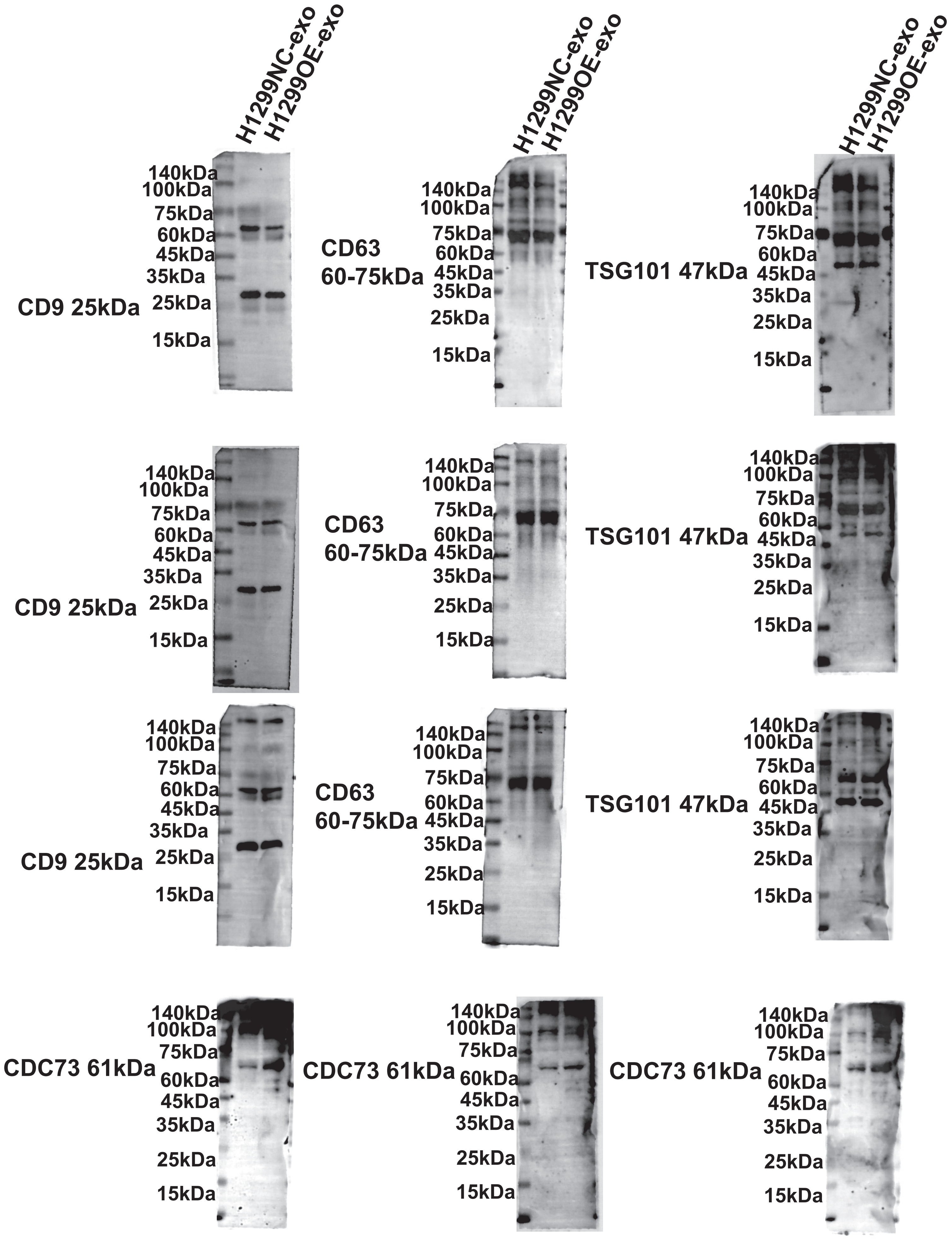
H1299NC-exo
H1299OE-exo
CD9 25kDa
CD63
60-75kDa
TSG101 47kDa
CDC73 61kDa
140kDa
100kDa
75kDa
60kDa
45kDa
35kDa
25kDa
15kDa

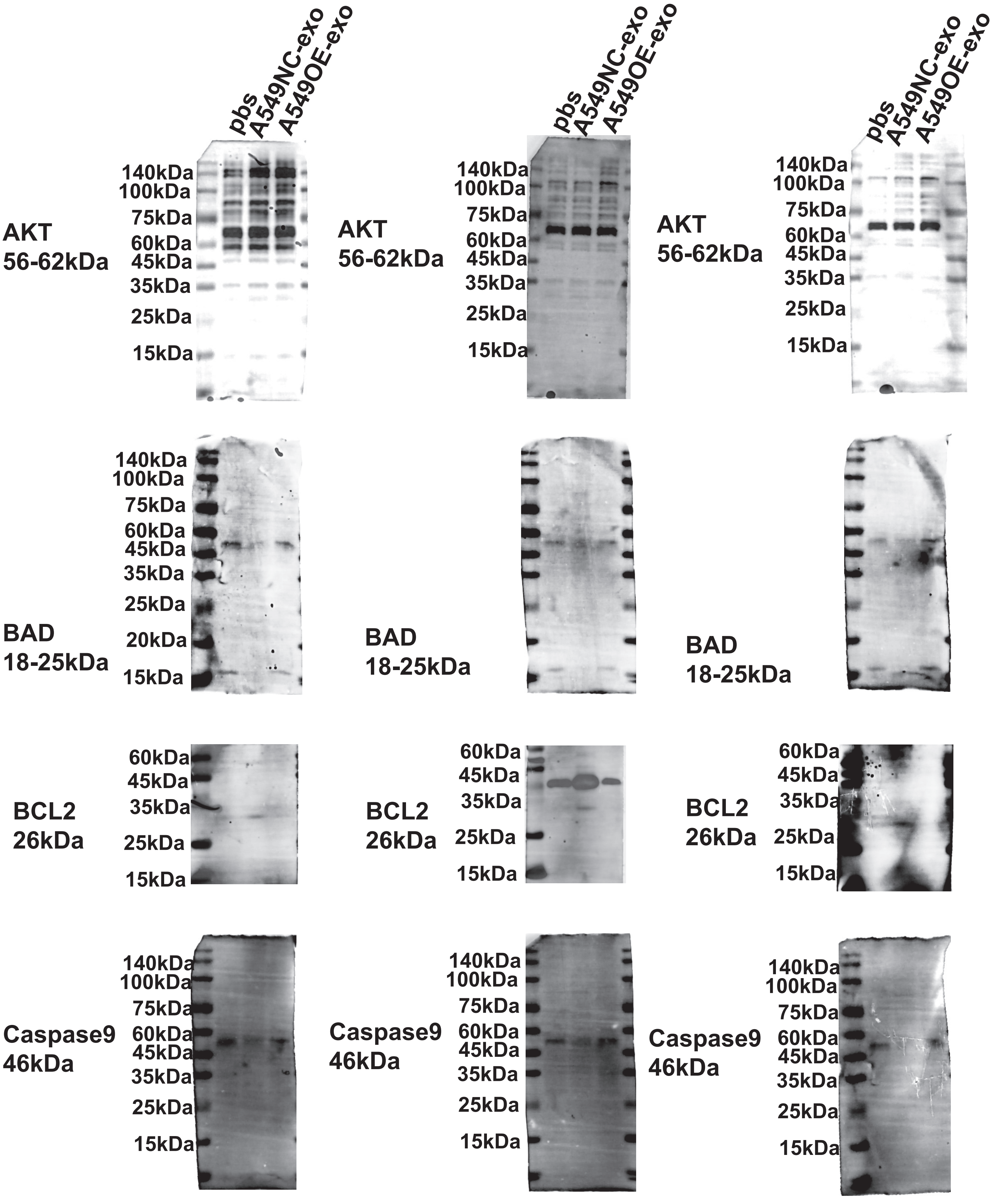
pbs
A549NC-exo
A549OE-exo
AKT
56-62kDa
BAD
18-25kDa
BCL2
26kDa
Caspase9
46kDa
140kDa
100kDa
75kDa
60kDa
45kDa
35kDa
25kDa
20kDa
15kDa

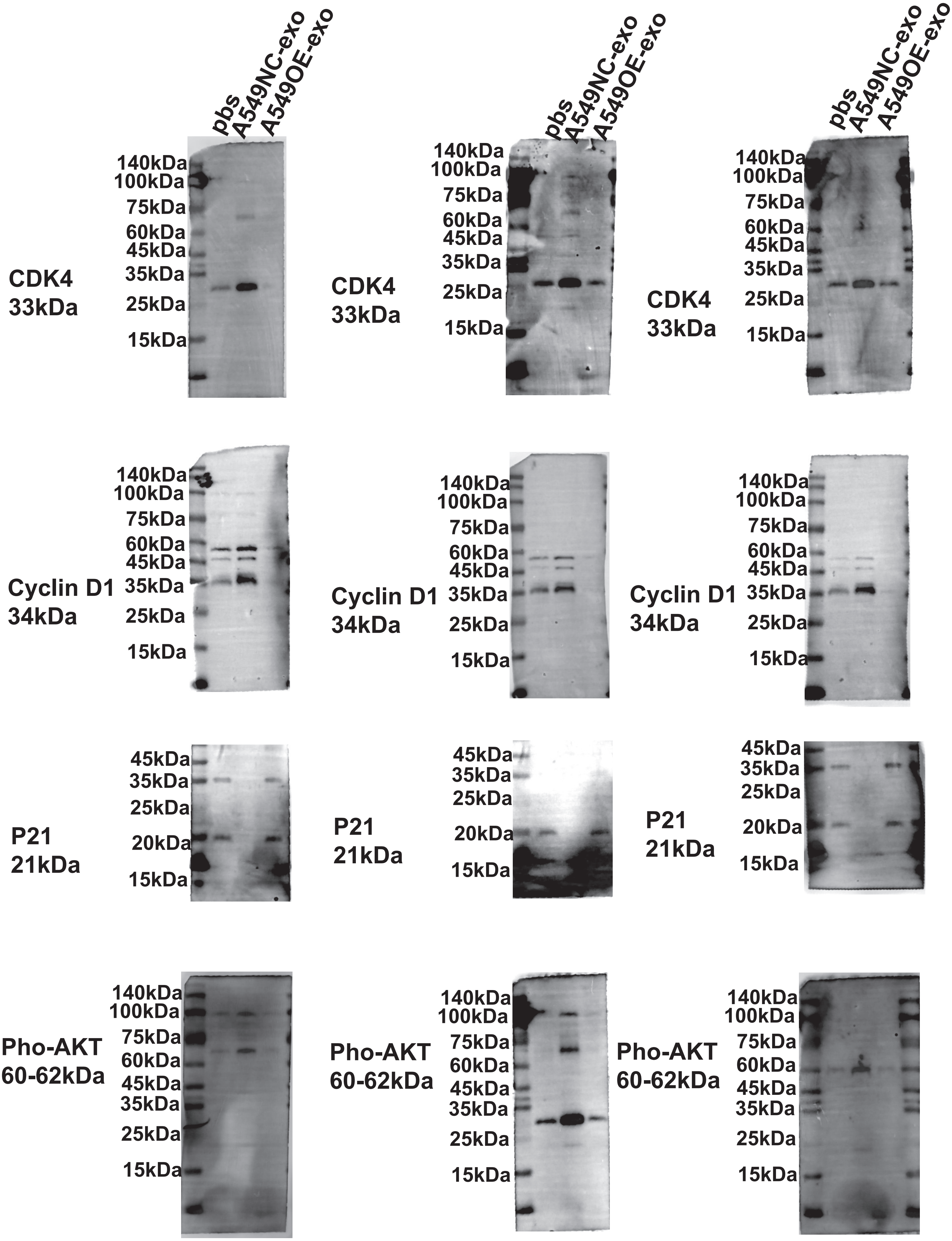
pbs
A549NC-exo
A549OE-exo
CDK4
33kDa
140kDa
100kDa
75kDa
60kDa
45kDa
35kDa
25kDa
15kDa
pbs
A549NC-exo
A549OE-exo
CDK4
33kDa
140kDa
100kDa
75kDa
60kDa
45kDa
35kDa
25kDa
15kDa
pbs
A549NC-exo
A549OE-exo
CDK4
33kDa
140kDa
100kDa
75kDa
60kDa
45kDa
35kDa
25kDa
15kDa
Cyclin D1
34kDa
140kDa
100kDa
75kDa
60kDa
45kDa
35kDa
25kDa
15kDa
Cyclin D1
34kDa
140kDa
100kDa
75kDa
60kDa
45kDa
35kDa
25kDa
15kDa
Cyclin D1
34kDa
140kDa
100kDa
75kDa
60kDa
45kDa
35kDa
25kDa
15kDa
P21
21kDa
45kDa
35kDa
25kDa
20kDa
15kDa
P21
21kDa
45kDa
35kDa
25kDa
20kDa
15kDa
P21
21kDa
45kDa
35kDa
25kDa
20kDa
15kDa
Pho-AKT
60-62kDa
140kDa
100kDa
75kDa
60kDa
45kDa
35kDa
25kDa
15kDa
Pho-AKT
60-62kDa
140kDa
100kDa
75kDa
60kDa
45kDa
35kDa
25kDa
15kDa
Pho-AKT
60-62kDa
140kDa
100kDa
75kDa
60kDa
45kDa
35kDa
25kDa
15kDa

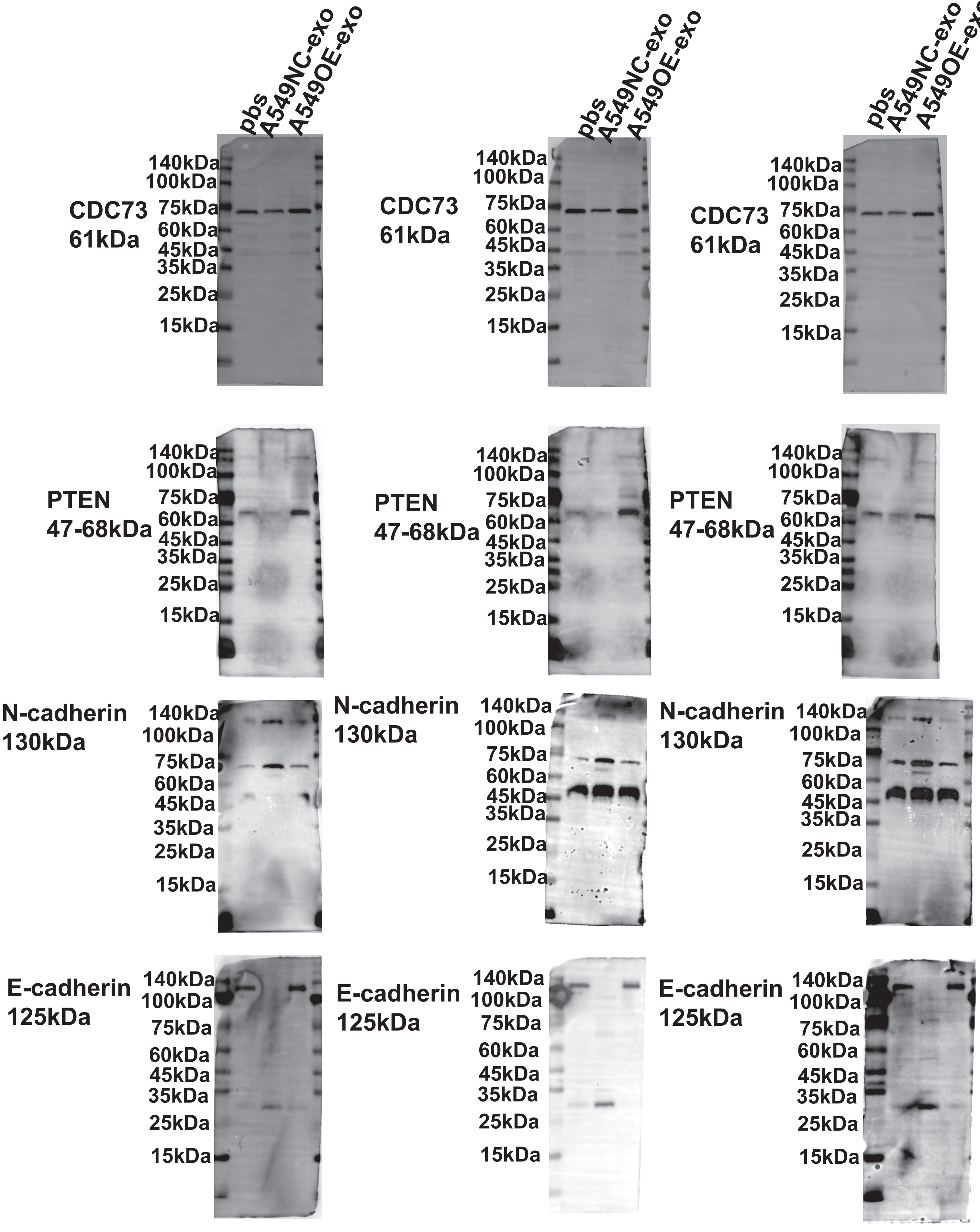

pbs
A549NC-exo
A549OE-exo
CDC73
61kDa
PTEN
47-68kDa
N-cadherin
130kDa
E-cadherin
125kDa
140kDa
100kDa
75kDa
60kDa
45kDa
35kDa
25kDa
15kDa

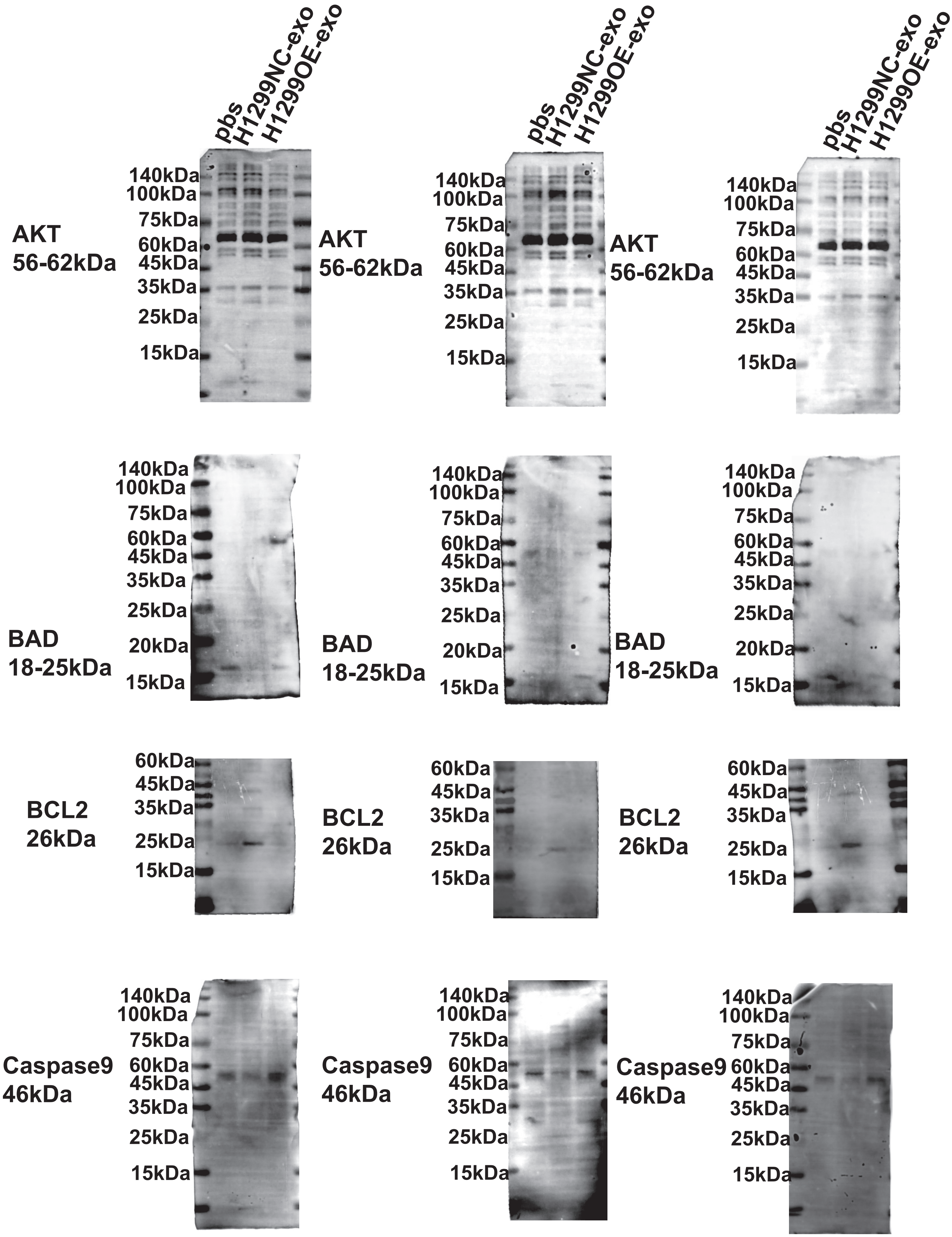
pbs
H1299NC-exo
H1299OE-exo
AKT
56-62kDa
BAD
18-25kDa
BCL2
26kDa
Caspase9
46kDa
140kDa
100kDa
75kDa
60kDa
45kDa
35kDa
25kDa
20kDa
15kDa

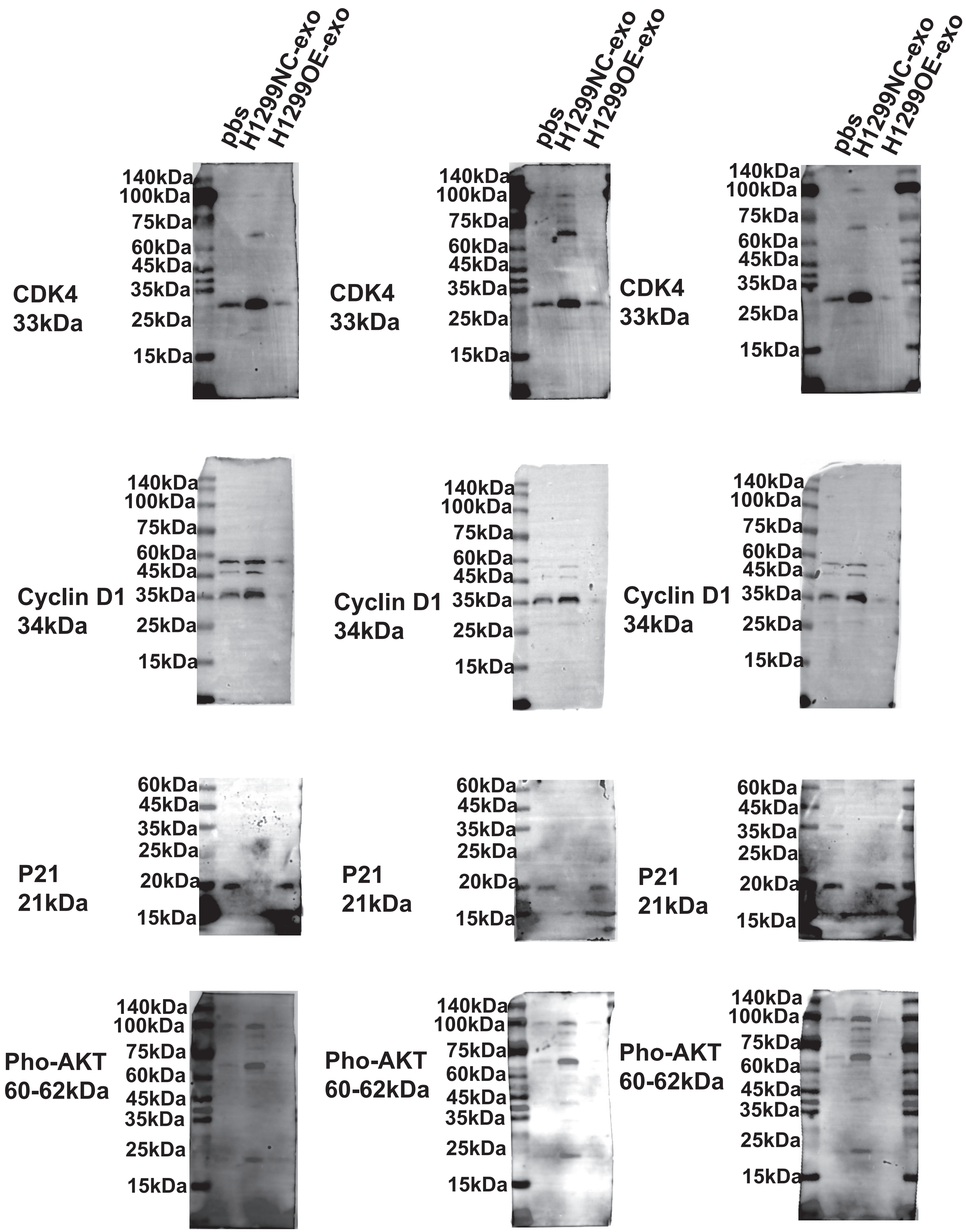

pbs
H1299NC-exo
H1299OE-exo
CDK4
33kDa
Cyclin D1
34kDa
P21
21kDa
Pho-AKT
60-62kDa
140kDa
100kDa
75kDa
60kDa
45kDa
35kDa
25kDa
20kDa
15kDa

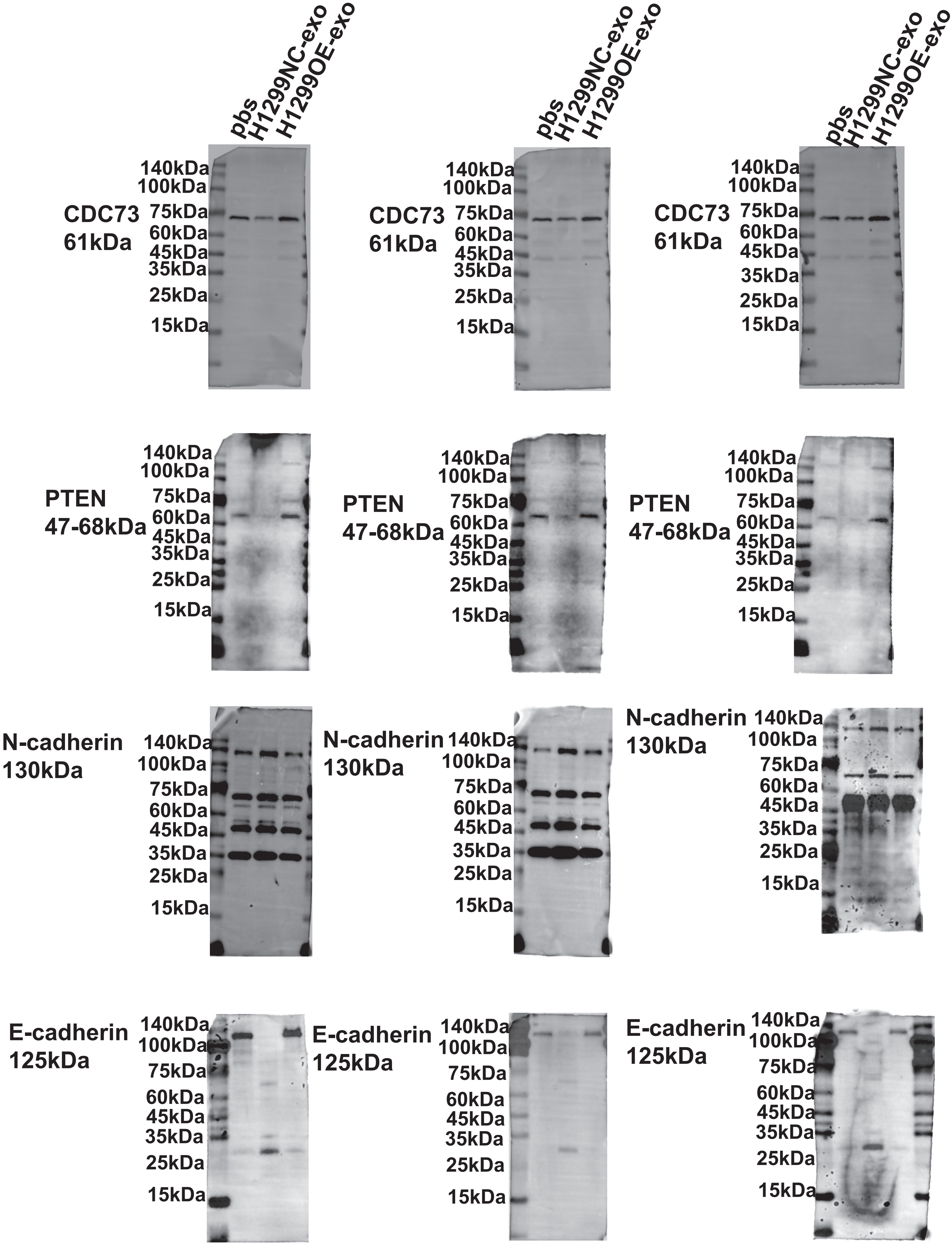
pbs
H1299NC-exo
H1299OE-exo
pbs
H1299NC-exo
H1299OE-exo
pbs
H1299NC-exo
H1299OE-exo
CDC73
61kDa
140kDa
100kDa
75kDa
60kDa
45kDa
35kDa
25kDa
15kDa
CDC73
61kDa
140kDa
100kDa
75kDa
60kDa
45kDa
35kDa
25kDa
15kDa
CDC73
61kDa
140kDa
100kDa
75kDa
60kDa
45kDa
35kDa
25kDa
15kDa
PTEN
47-68kDa
140kDa
100kDa
75kDa
60kDa
45kDa
35kDa
25kDa
15kDa
PTEN
47-68kDa
140kDa
100kDa
75kDa
60kDa
45kDa
35kDa
25kDa
15kDa
PTEN
47-68kDa
140kDa
100kDa
75kDa
60kDa
45kDa
35kDa
25kDa
15kDa
N-cadherin
130kDa
140kDa
100kDa
75kDa
60kDa
45kDa
35kDa
25kDa
15kDa
N-cadherin
130kDa
140kDa
100kDa
75kDa
60kDa
45kDa
35kDa
25kDa
15kDa
N-cadherin
130kDa
140kDa
100kDa
75kDa
60kDa
45kDa
35kDa
25kDa
15kDa
E-cadherin
125kDa
140kDa
100kDa
75kDa
60kDa
45kDa
35kDa
25kDa
15kDa
E-cadherin
125kDa
140kDa
100kDa
75kDa
60kDa
45kDa
35kDa
25kDa
15kDa
E-cadherin
125kDa
140kDa
100kDa
75kDa
60kDa
45kDa
35kDa
25kDa
15kDa

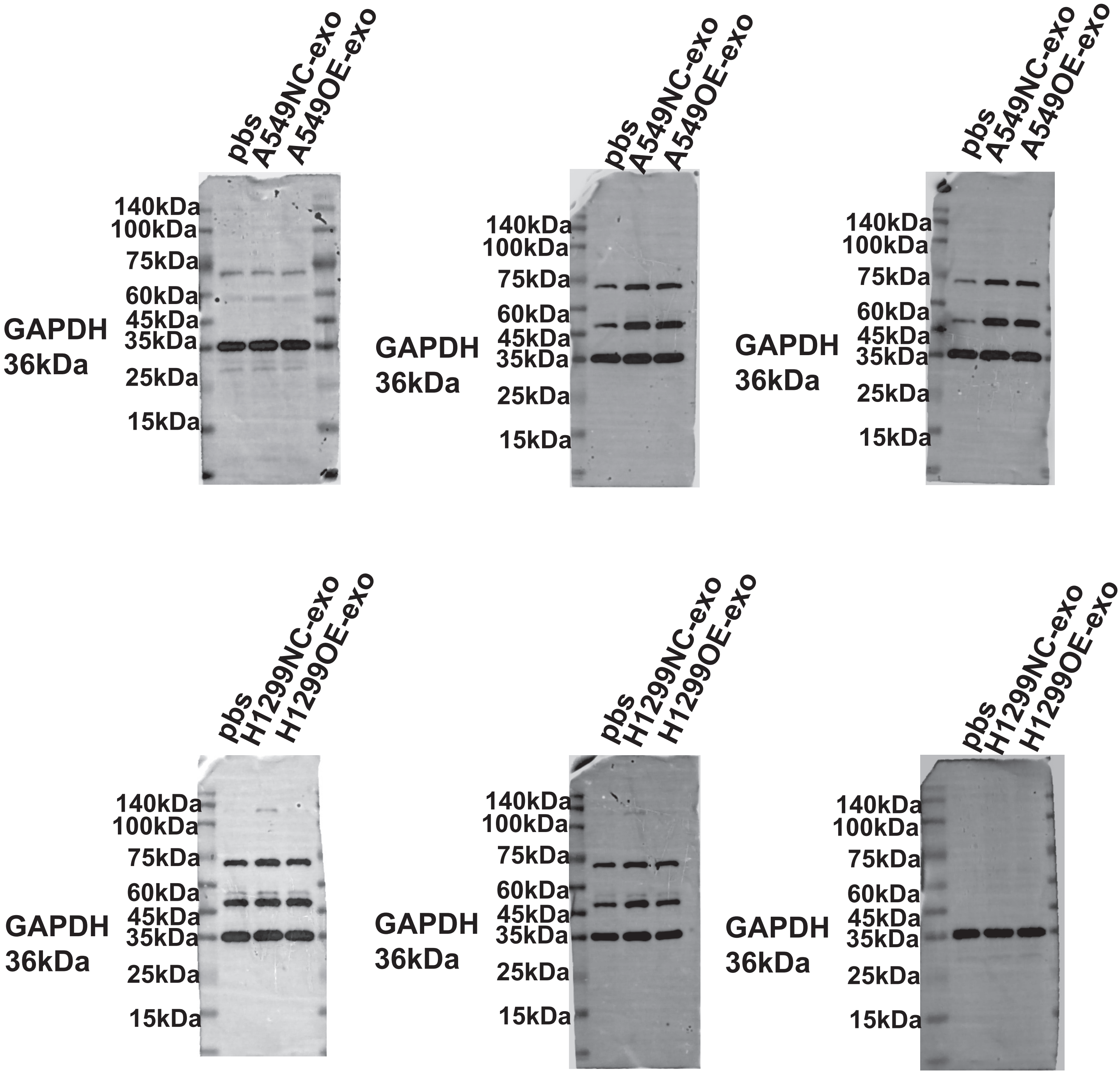

pbs
A549NC-exo
A549OE-exo
140kDa
100kDa
75kDa
60kDa
45kDa
35kDa
25kDa
15kDa
GAPDH
36kDa
pbs
A549NC-exo
A549OE-exo
140kDa
100kDa
75kDa
60kDa
45kDa
35kDa
25kDa
15kDa
GAPDH
36kDa
pbs
A549NC-exo
A549OE-exo
140kDa
100kDa
75kDa
60kDa
45kDa
35kDa
25kDa
15kDa
GAPDH
36kDa
pbs
H1299NC-exo
H1299OE-exo
140kDa
100kDa
75kDa
60kDa
45kDa
35kDa
25kDa
15kDa
GAPDH
36kDa
pbs
H1299NC-exo
H1299OE-exo
140kDa
100kDa
75kDa
60kDa
45kDa
35kDa
25kDa
15kDa
GAPDH
36kDa
pbs
H1299NC-exo
H1299OE-exo
140kDa
100kDa
75kDa
60kDa
45kDa
35kDa
25kDa
15kDa
GAPDH
36kDa

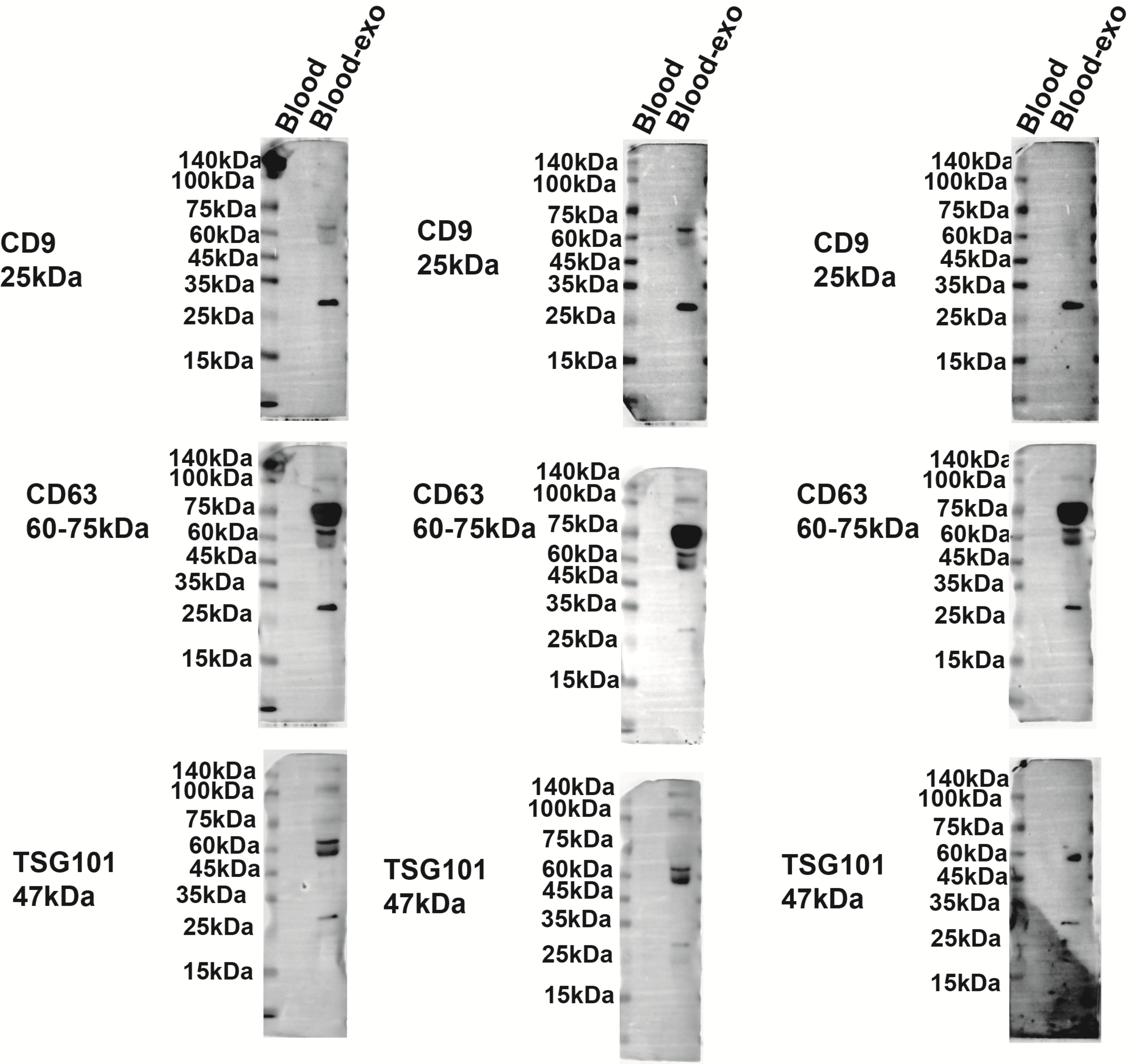

Blood
Blood-exo
Blood
Blood-exo
Blood
Blood-exo
CD9
25kDa
140kDa
100kDa
75kDa
60kDa
45kDa
35kDa
25kDa
15kDa
CD9
25kDa
140kDa
100kDa
75kDa
60kDa
45kDa
35kDa
25kDa
15kDa
CD9
25kDa
140kDa
100kDa
75kDa
60kDa
45kDa
35kDa
25kDa
15kDa
CD63
60-75kDa
140kDa
100kDa
75kDa
60kDa
45kDa
35kDa
25kDa
15kDa
CD63
60-75kDa
140kDa
100kDa
75kDa
60kDa
45kDa
35kDa
25kDa
15kDa
CD63
60-75kDa
140kDa
100kDa
75kDa
60kDa
45kDa
35kDa
25kDa
15kDa
TSG101
47kDa
140kDa
100kDa
75kDa
60kDa
45kDa
35kDa
25kDa
15kDa
TSG101
47kDa
140kDa
100kDa
75kDa
60kDa
45kDa
35kDa
25kDa
15kDa
TSG101
47kDa
140kDa
100kDa
75kDa
60kDa
45kDa
35kDa
25kDa
15kDa

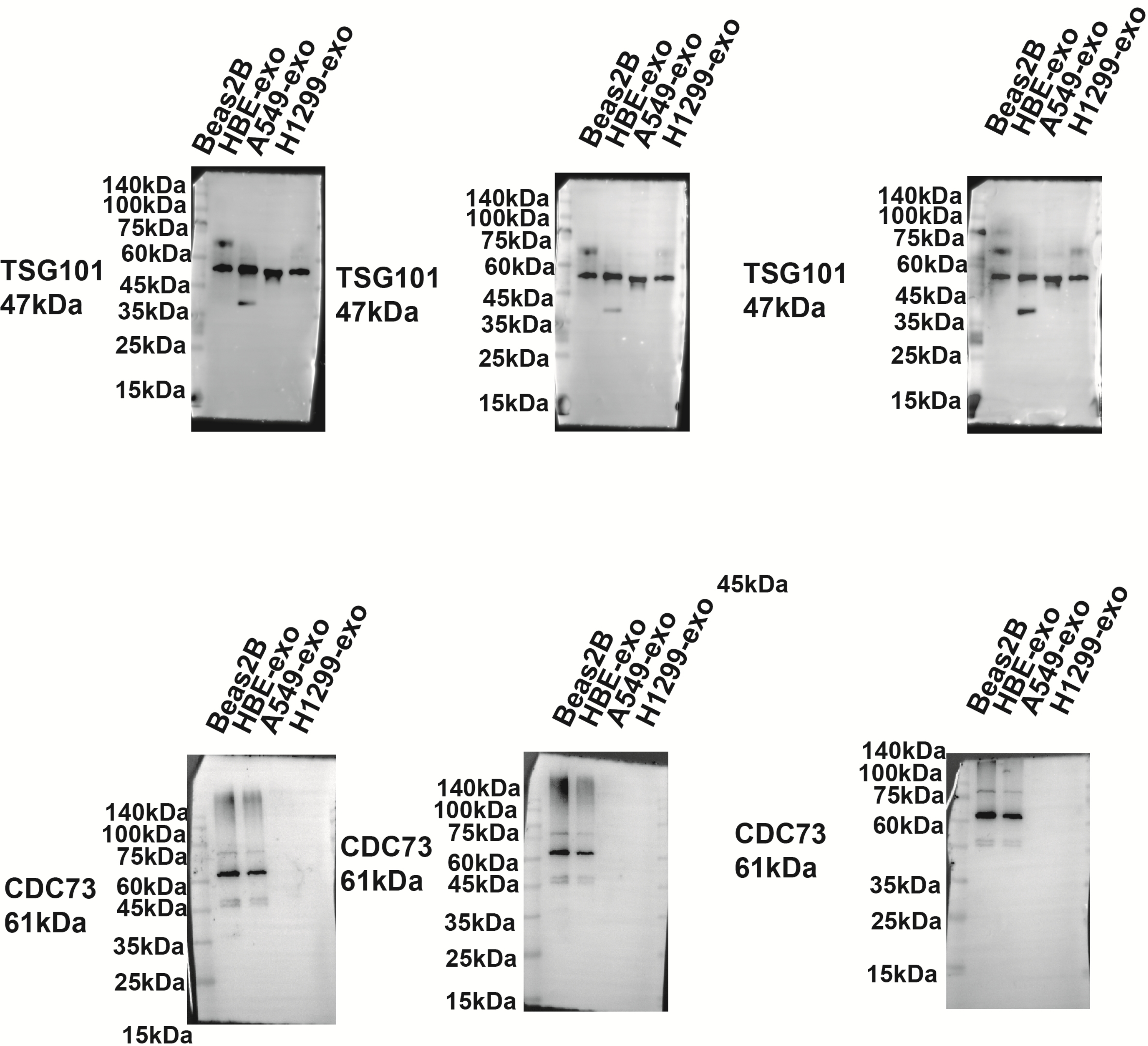
Beas2B
HBE-exo
A549-exo
H1299-exo
140kDa
100kDa
75kDa
60kDa
45kDa
35kDa
25kDa
15kDa
TSG101
47kDa
Beas2B
HBE-exo
A549-exo
H1299-exo
140kDa
100kDa
75kDa
60kDa
45kDa
35kDa
25kDa
15kDa
TSG101
47kDa
Beas2B
HBE-exo
A549-exo
H1299-exo
140kDa
100kDa
75kDa
60kDa
45kDa
35kDa
25kDa
15kDa
TSG101
47kDa
Beas2B
HBE-exo
A549-exo
H1299-exo
140kDa
100kDa
75kDa
60kDa
45kDa
35kDa
25kDa
15kDa
CDC73
61kDa
45kDa
Beas2B
HBE-exo
A549-exo
H1299-exo
140kDa
100kDa
75kDa
60kDa
45kDa
35kDa
25kDa
15kDa
CDC73
61kDa
Beas2B
HBE-exo
A549-exo
H1299-exo
140kDa
100kDa
75kDa
60kDa
35kDa
25kDa
15kDa
CDC73
61kDa

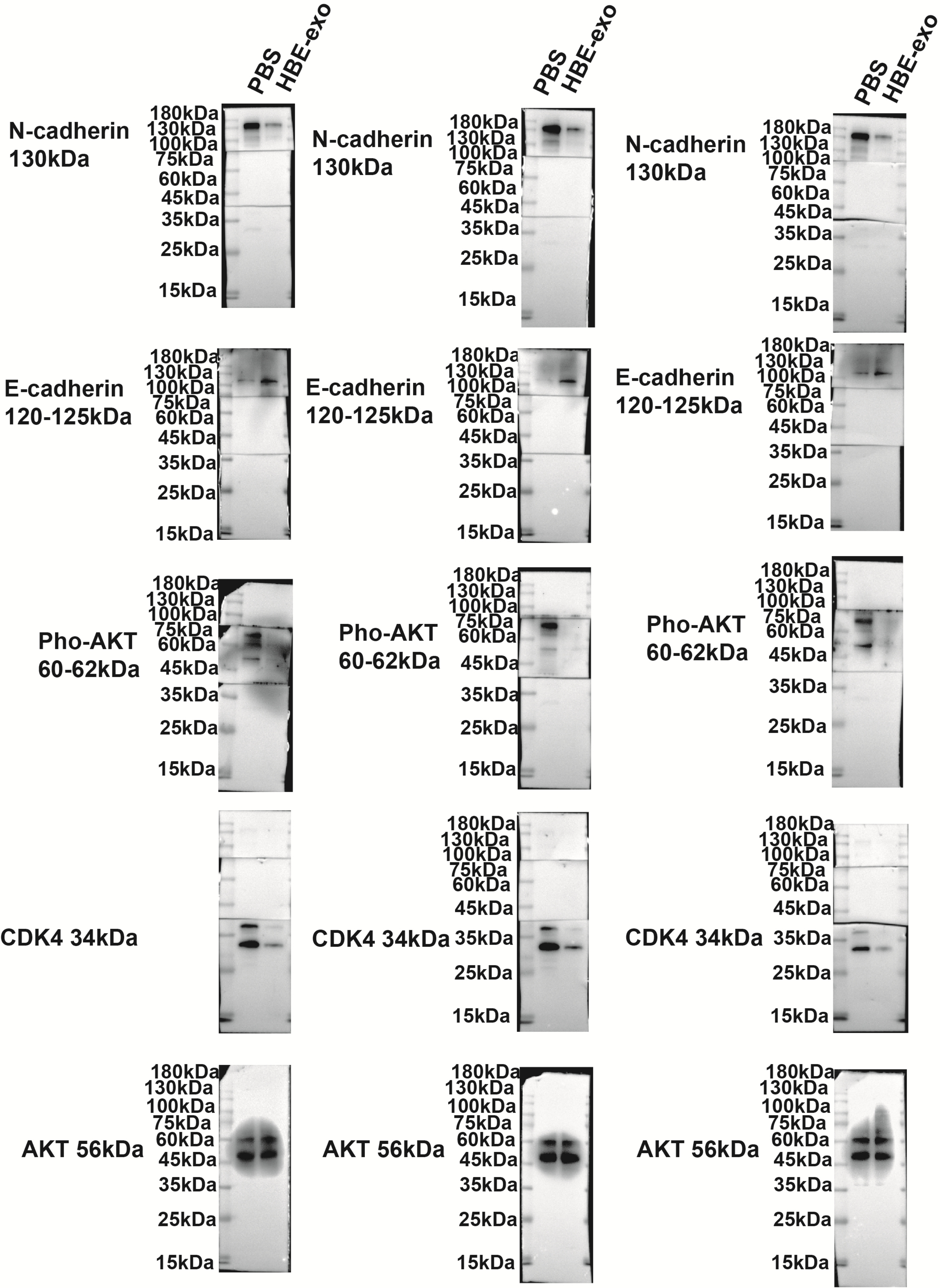
PBS
HBE-exo
N-cadherin 130kDa
E-cadherin 120-125kDa
Pho-AKT 60-62kDa
CDK4 34kDa
AKT 56kDa
180kDa
130kDa
100kDa
75kDa
60kDa
45kDa
35kDa
25kDa
15kDa

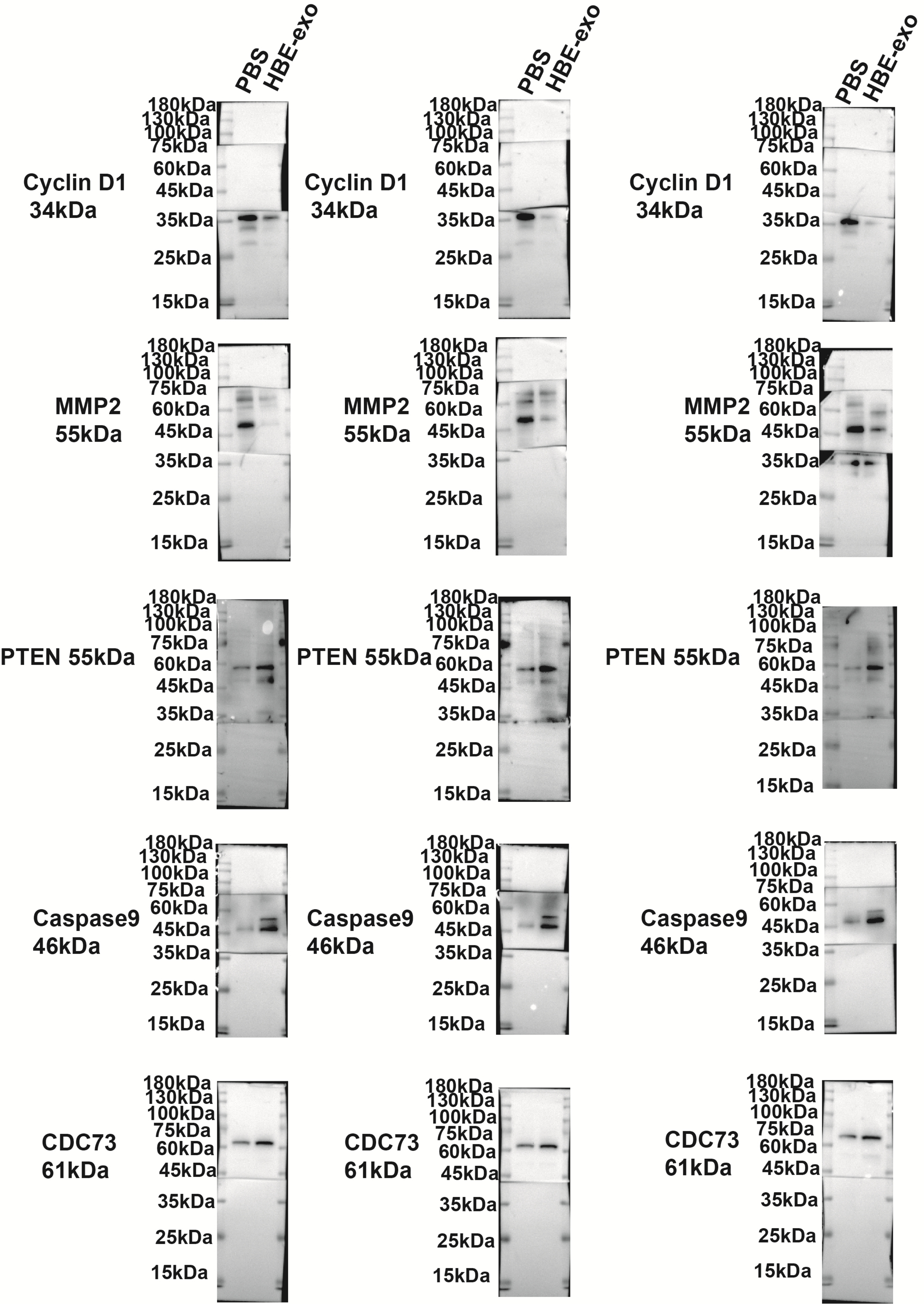

PBS
HBE-exo
Cyclin D1 34kDa
MMP2 55kDa
PTEN 55kDa
Caspase9 46kDa
CDC73 61kDa
180kDa
130kDa
100kDa
75kDa
60kDa
45kDa
35kDa
25kDa
15kDa

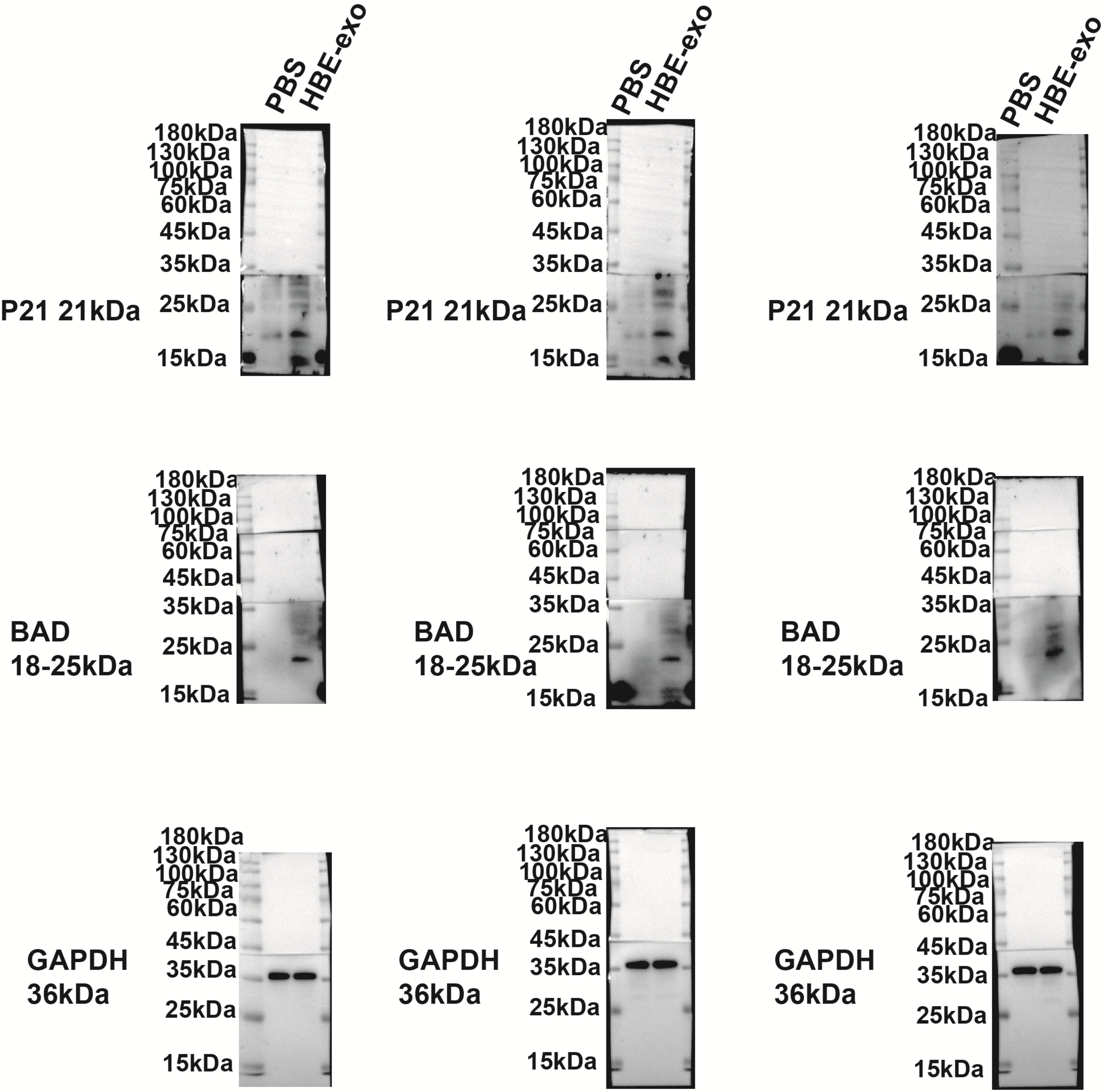
PBS
HBE-exo
P21 21kDa
BAD
18-25kDa
GAPDH
36kDa
180kDa
130kDa
100kDa
75kDa
60kDa
45kDa
35kDa
25kDa
15kDa

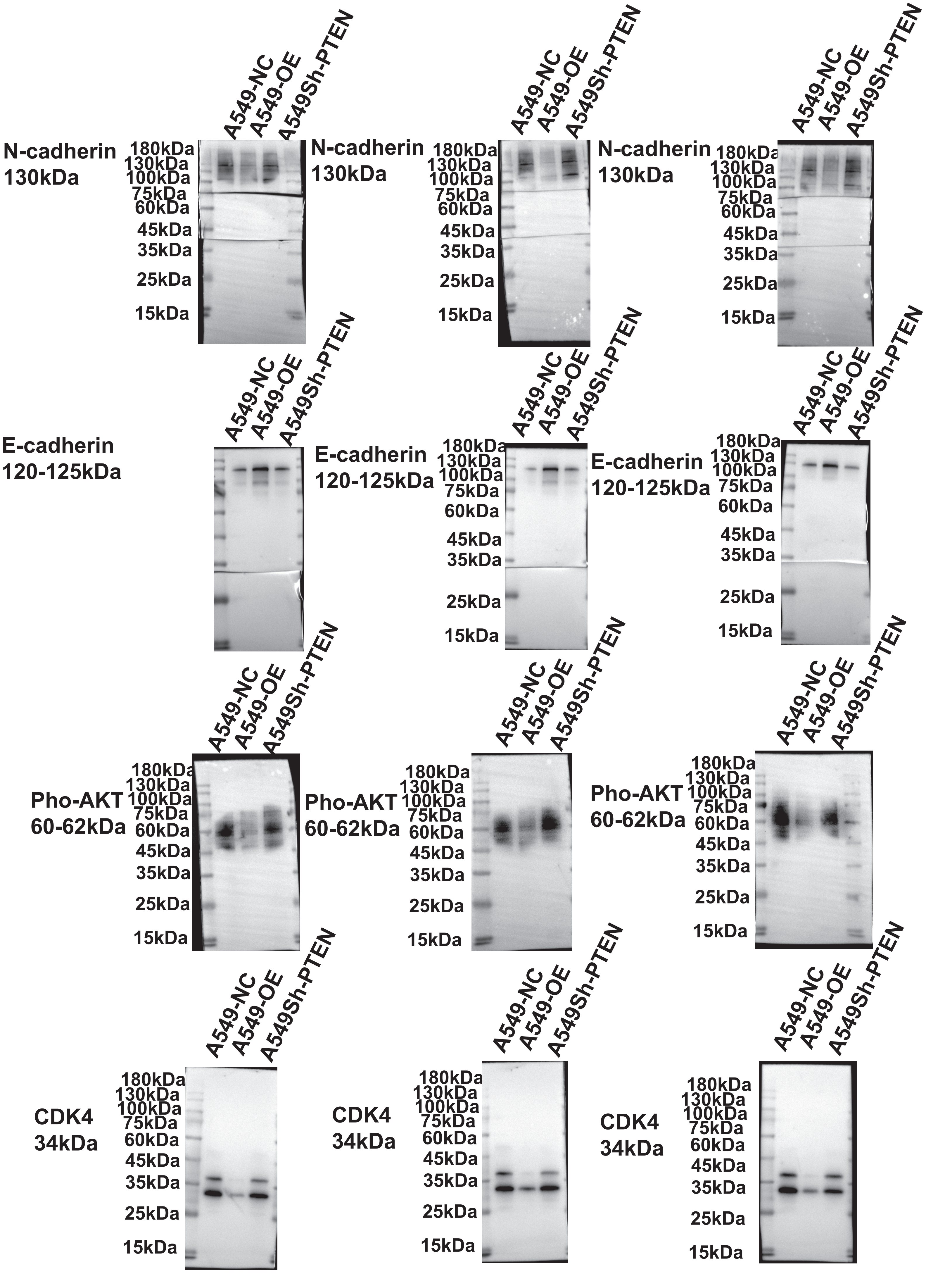

A549-NC
A549-OE
A549Sh-PTEN
N-cadherin
130kDa
180kDa
130kDa
100kDa
75kDa
60kDa
45kDa
35kDa
25kDa
15kDa
N-cadherin
130kDa
N-cadherin
130kDa
E-cadherin
120-125kDa
E-cadherin
120-125kDa
E-cadherin
120-125kDa
Pho-AKT
60-62kDa
Pho-AKT
60-62kDa
Pho-AKT
60-62kDa
CDK4
34kDa
CDK4
34kDa
CDK4
34kDa

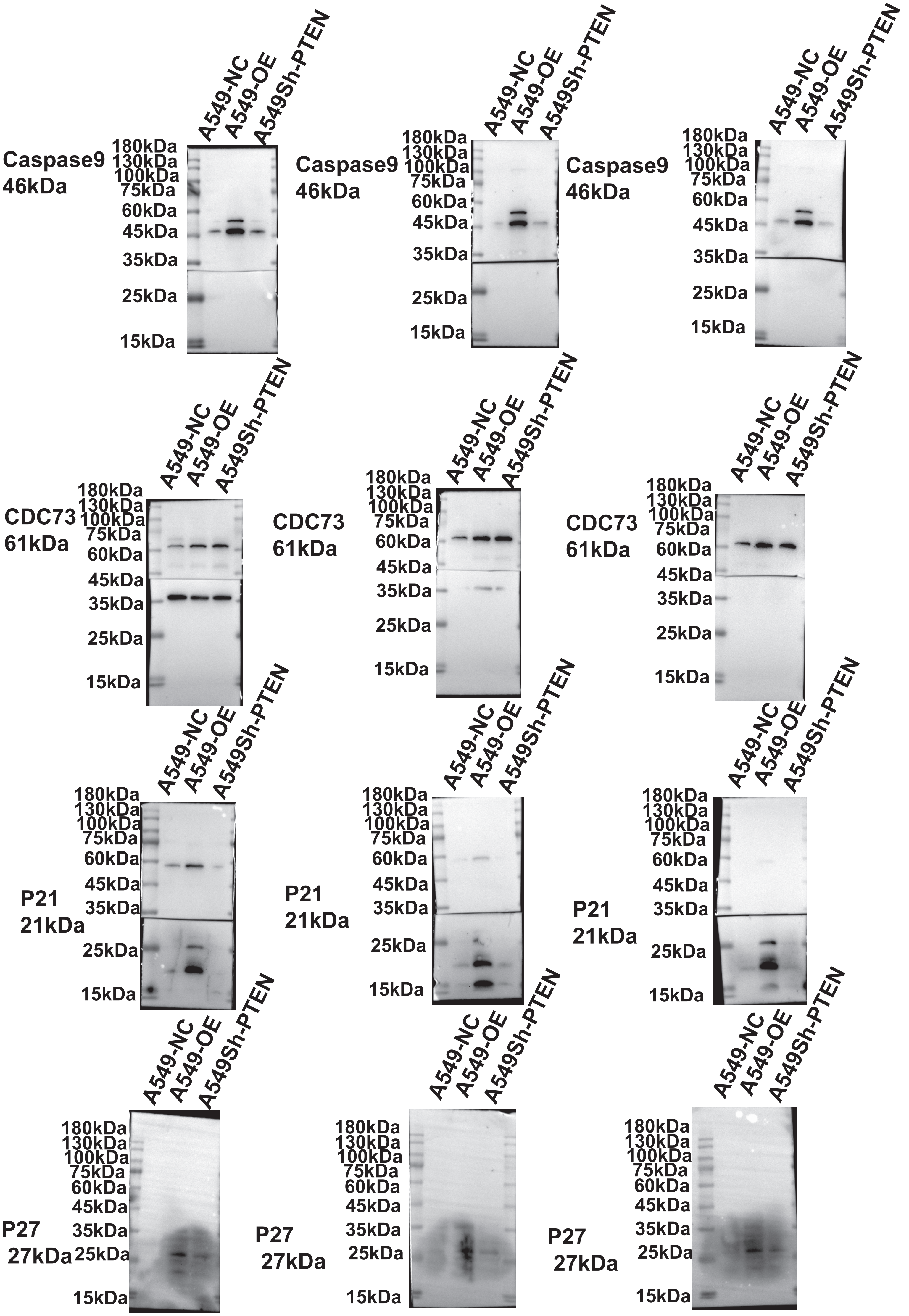
A549-NC
A549-OE
A549Sh-PTEN
Caspase9
46kDa
180kDa
130kDa
100kDa
75kDa
60kDa
45kDa
35kDa
25kDa
15kDa
A549-NC
A549-OE
A549Sh-PTEN
Caspase9
46kDa
A549-NC
A549-OE
A549Sh-PTEN
Caspase9
46kDa
A549-NC
A549-OE
A549Sh-PTEN
CDC73
61kDa
A549-NC
A549-OE
A549Sh-PTEN
CDC73
61kDa
A549-NC
A549-OE
A549Sh-PTEN
CDC73
61kDa
A549-NC
A549-OE
A549Sh-PTEN
P21
21kDa
A549-NC
A549-OE
A549Sh-PTEN
P21
21kDa
A549-NC
A549-OE
A549Sh-PTEN
P21
21kDa
A549-NC
A549-OE
A549Sh-PTEN
P27
27kDa
A549-NC
A549-OE
A549Sh-PTEN
P27
27kDa
A549-NC
A549-OE
A549Sh-PTEN
P27
27kDa

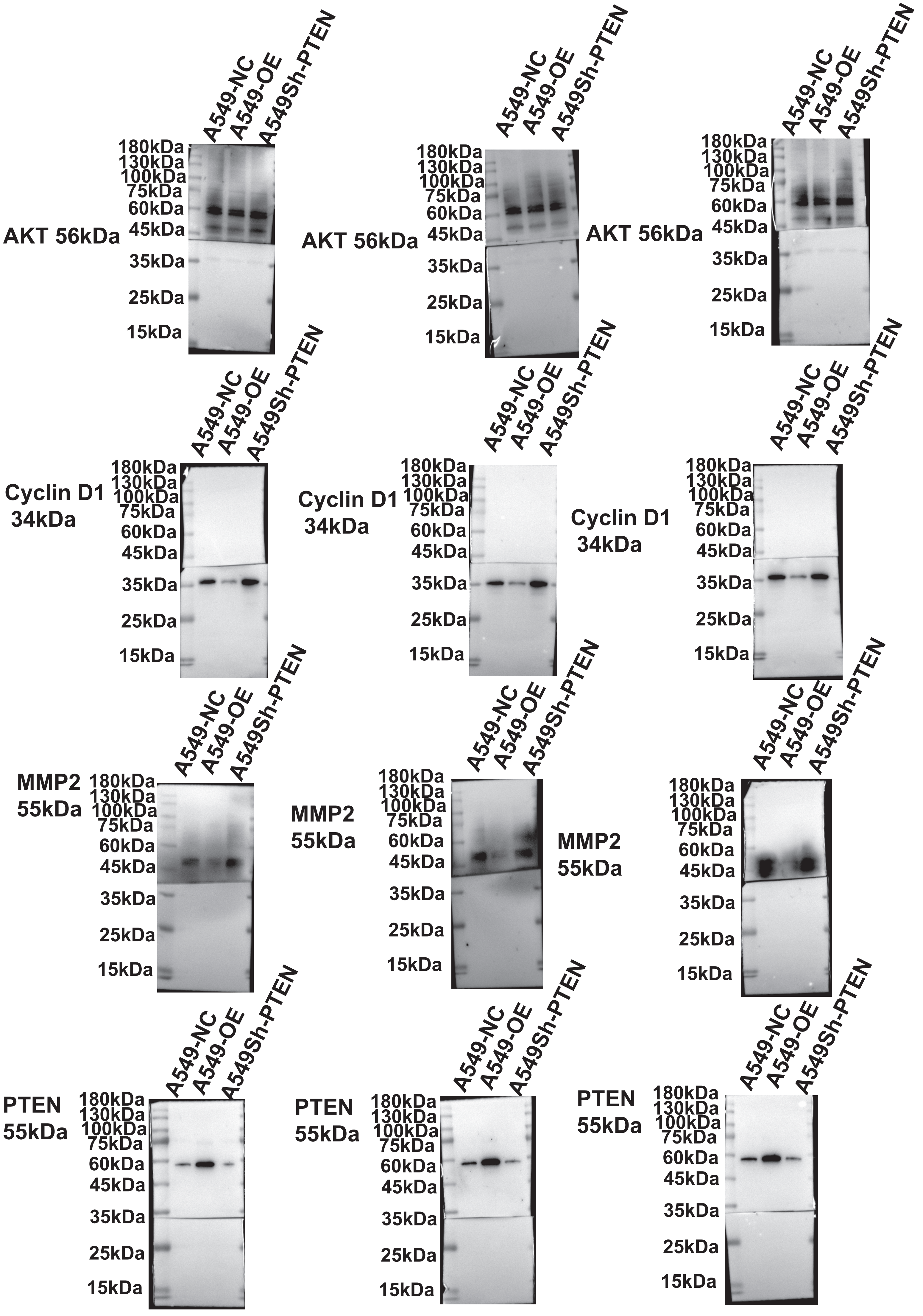
A549-NC
A549-OE
A549Sh-PTEN
AKT 56kDa
Cyclin D1 34kDa
MMP2 55kDa
PTEN 55kDa
180kDa
130kDa
100kDa
75kDa
60kDa
45kDa
35kDa
25kDa
15kDa

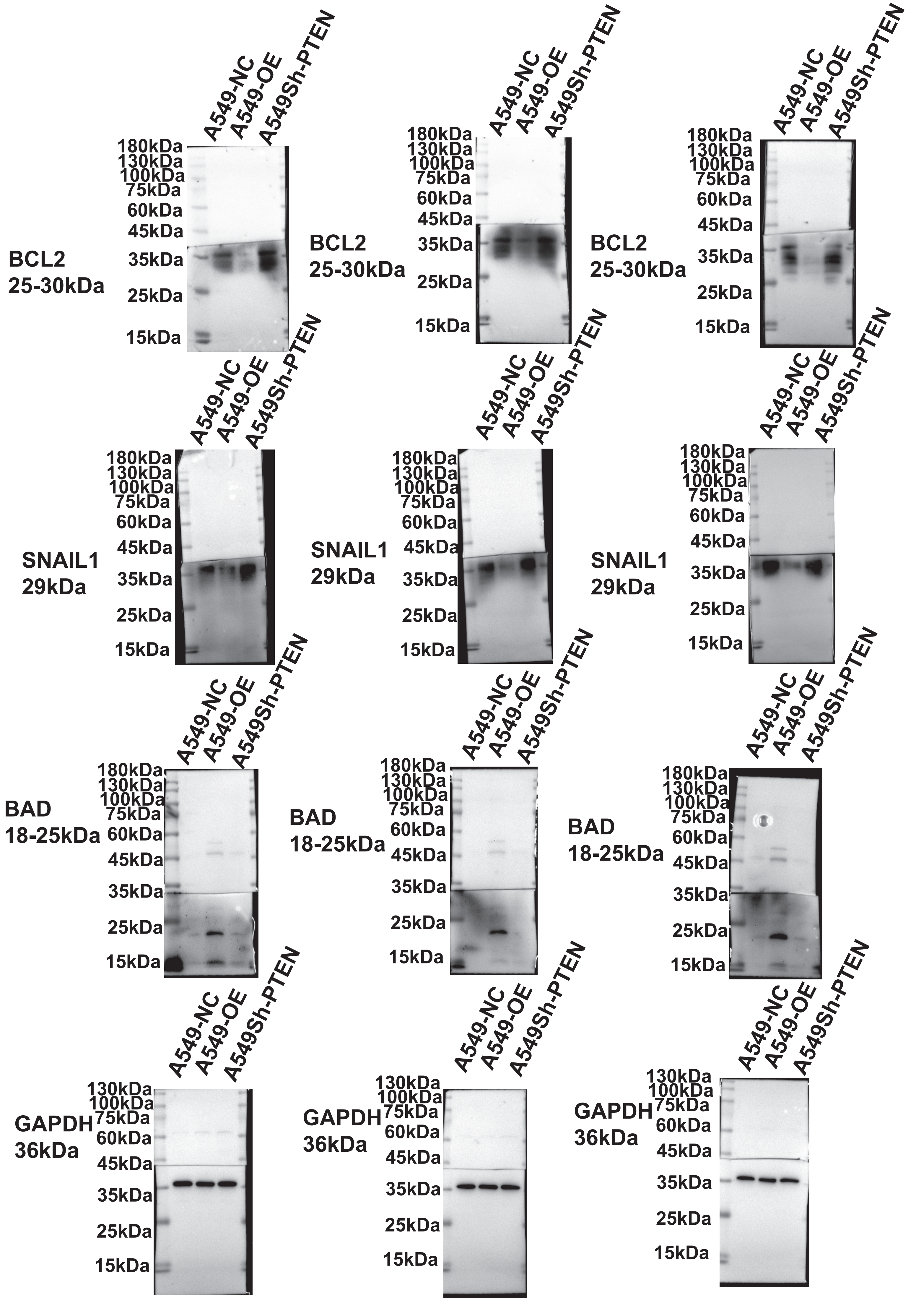

A549-NC
A549-OE
A549Sh-PTEN
180kDa
130kDa
100kDa
75kDa
60kDa
45kDa
35kDa
25kDa
15kDa
BCL2
25-30kDa
BCL2
25-30kDa
BCL2
25-30kDa
SNAIL1
29kDa
SNAIL1
29kDa
SNAIL1
29kDa
BAD
18-25kDa
BAD
18-25kDa
BAD
18-25kDa
GAPDH
36kDa
GAPDH
36kDa
GAPDH
36kDa

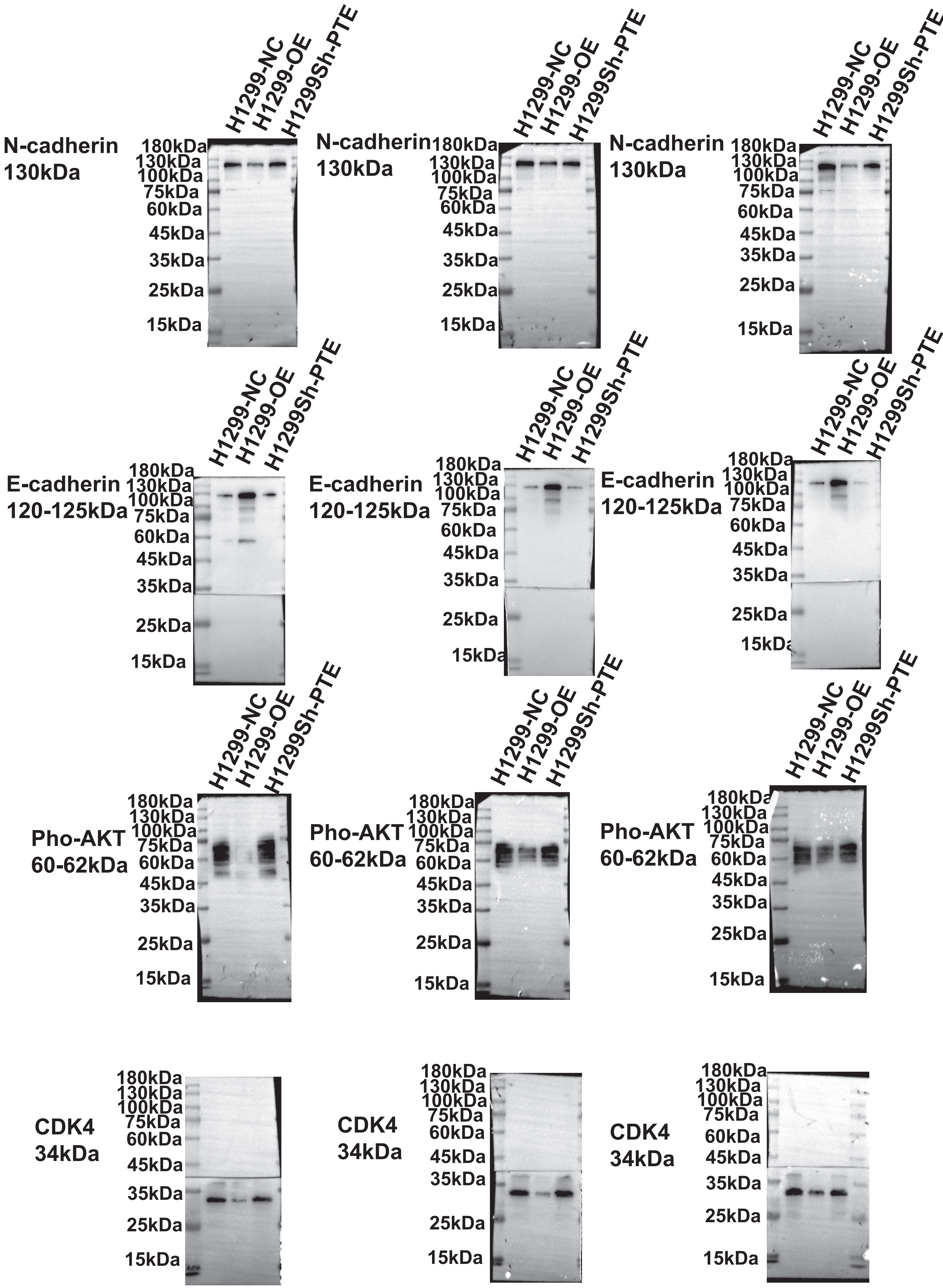

N-cadherin
130kDa
H1299-NC
H1299-OE
H1299Sh-PTE
180kDa
130kDa
100kDa
75kDa
60kDa
45kDa
35kDa
25kDa
15kDa
N-cadherin
130kDa
H1299-NC
H1299-OE
H1299Sh-PTE
180kDa
130kDa
100kDa
75kDa
60kDa
45kDa
35kDa
25kDa
15kDa
N-cadherin
130kDa
H1299-NC
H1299-OE
H1299Sh-PTE
180kDa
130kDa
100kDa
75kDa
60kDa
45kDa
35kDa
25kDa
15kDa
E-cadherin
120-125kDa
H1299-NC
H1299-OE
H1299Sh-PTE
180kDa
130kDa
100kDa
75kDa
60kDa
45kDa
35kDa
25kDa
15kDa
E-cadherin
120-125kDa
H1299-NC
H1299-OE
H1299Sh-PTE
180kDa
130kDa
100kDa
75kDa
60kDa
45kDa
35kDa
25kDa
15kDa
E-cadherin
120-125kDa
H1299-NC
H1299-OE
H1299Sh-PTE
180kDa
130kDa
100kDa
75kDa
60kDa
45kDa
35kDa
25kDa
15kDa
Pho-AKT
60-62kDa
H1299-NC
H1299-OE
H1299Sh-PTE
180kDa
130kDa
100kDa
75kDa
60kDa
45kDa
35kDa
25kDa
15kDa
Pho-AKT
60-62kDa
H1299-NC
H1299-OE
H1299Sh-PTE
180kDa
130kDa
100kDa
75kDa
60kDa
45kDa
35kDa
25kDa
15kDa
Pho-AKT
60-62kDa
H1299-NC
H1299-OE
H1299Sh-PTE
180kDa
130kDa
100kDa
75kDa
60kDa
45kDa
35kDa
25kDa
15kDa
CDK4
34kDa
180kDa
130kDa
100kDa
75kDa
60kDa
45kDa
35kDa
25kDa
15kDa
CDK4
34kDa
180kDa
130kDa
100kDa
75kDa
60kDa
45kDa
35kDa
25kDa
15kDa
CDK4
34kDa
180kDa
130kDa
100kDa
75kDa
60kDa
45kDa
35kDa
25kDa
15kDa

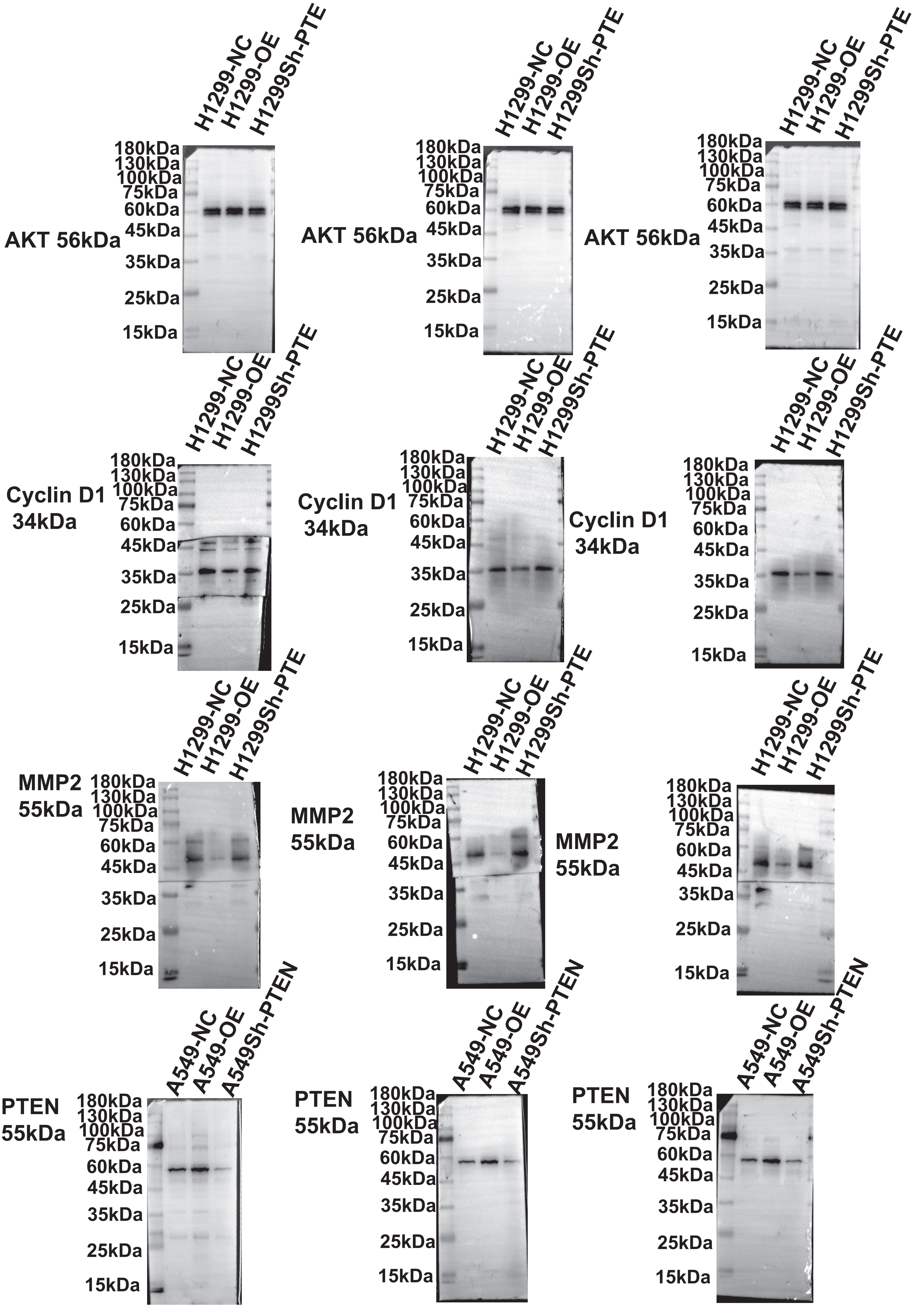

H1299-NC
H1299-OE
H1299Sh-PTE
AKT 56kDa
180kDa
130kDa
100kDa
75kDa
60kDa
45kDa
35kDa
25kDa
15kDa
Cyclin D1 34kDa
MMP2 55kDa
A549-NC
A549-OE
A549Sh-PTEN
PTEN 55kDa

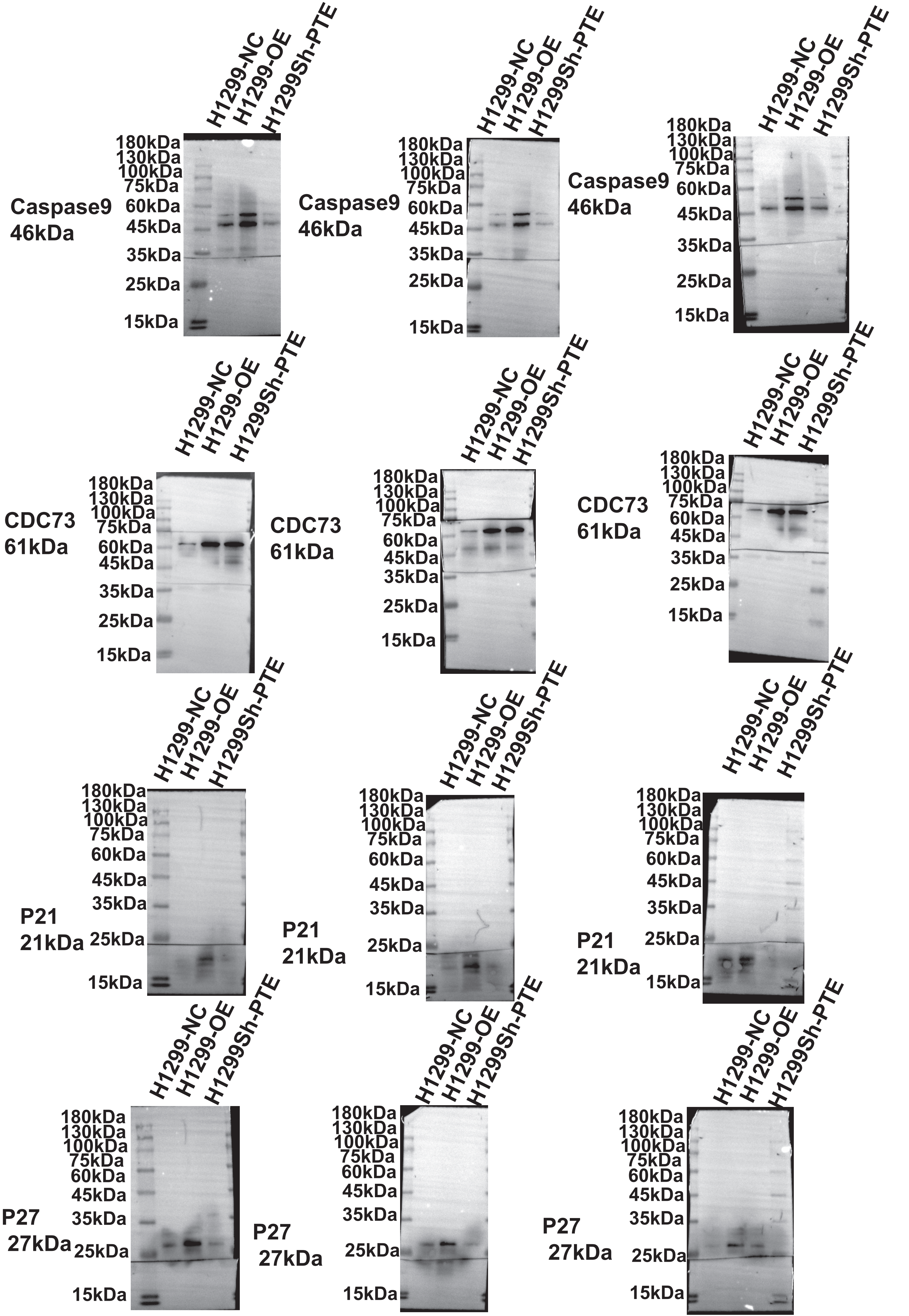

Caspase9
46kDa
H1299-NC
H1299-OE
H1299Sh-PTE
180kDa
130kDa
100kDa
75kDa
60kDa
45kDa
35kDa
25kDa
15kDa
Caspase9
46kDa
H1299-NC
H1299-OE
H1299Sh-PTE
180kDa
130kDa
100kDa
75kDa
60kDa
45kDa
35kDa
25kDa
15kDa
Caspase9
46kDa
H1299-NC
H1299-OE
H1299Sh-PTE
180kDa
130kDa
100kDa
75kDa
60kDa
45kDa
35kDa
25kDa
15kDa
CDC73
61kDa
H1299-NC
H1299-OE
H1299Sh-PTE
180kDa
130kDa
100kDa
75kDa
60kDa
45kDa
35kDa
25kDa
15kDa
CDC73
61kDa
H1299-NC
H1299-OE
H1299Sh-PTE
180kDa
130kDa
100kDa
75kDa
60kDa
45kDa
35kDa
25kDa
15kDa
CDC73
61kDa
H1299-NC
H1299-OE
H1299Sh-PTE
180kDa
130kDa
100kDa
75kDa
60kDa
45kDa
35kDa
25kDa
15kDa
P21
21kDa
H1299-NC
H1299-OE
H1299Sh-PTE
180kDa
130kDa
100kDa
75kDa
60kDa
45kDa
35kDa
25kDa
15kDa
P21
21kDa
H1299-NC
H1299-OE
H1299Sh-PTE
180kDa
130kDa
100kDa
75kDa
60kDa
45kDa
35kDa
25kDa
15kDa
P21
21kDa
H1299-NC
H1299-OE
H1299Sh-PTE
180kDa
130kDa
100kDa
75kDa
60kDa
45kDa
35kDa
25kDa
15kDa
P27
27kDa
H1299-NC
H1299-OE
H1299Sh-PTE
180kDa
130kDa
100kDa
75kDa
60kDa
45kDa
35kDa
25kDa
15kDa
P27
27kDa
H1299-NC
H1299-OE
H1299Sh-PTE
180kDa
130kDa
100kDa
75kDa
60kDa
45kDa
35kDa
25kDa
15kDa
P27
27kDa
H1299-NC
H1299-OE
H1299Sh-PTE
180kDa
130kDa
100kDa
75kDa
60kDa
45kDa
35kDa
25kDa
15kDa

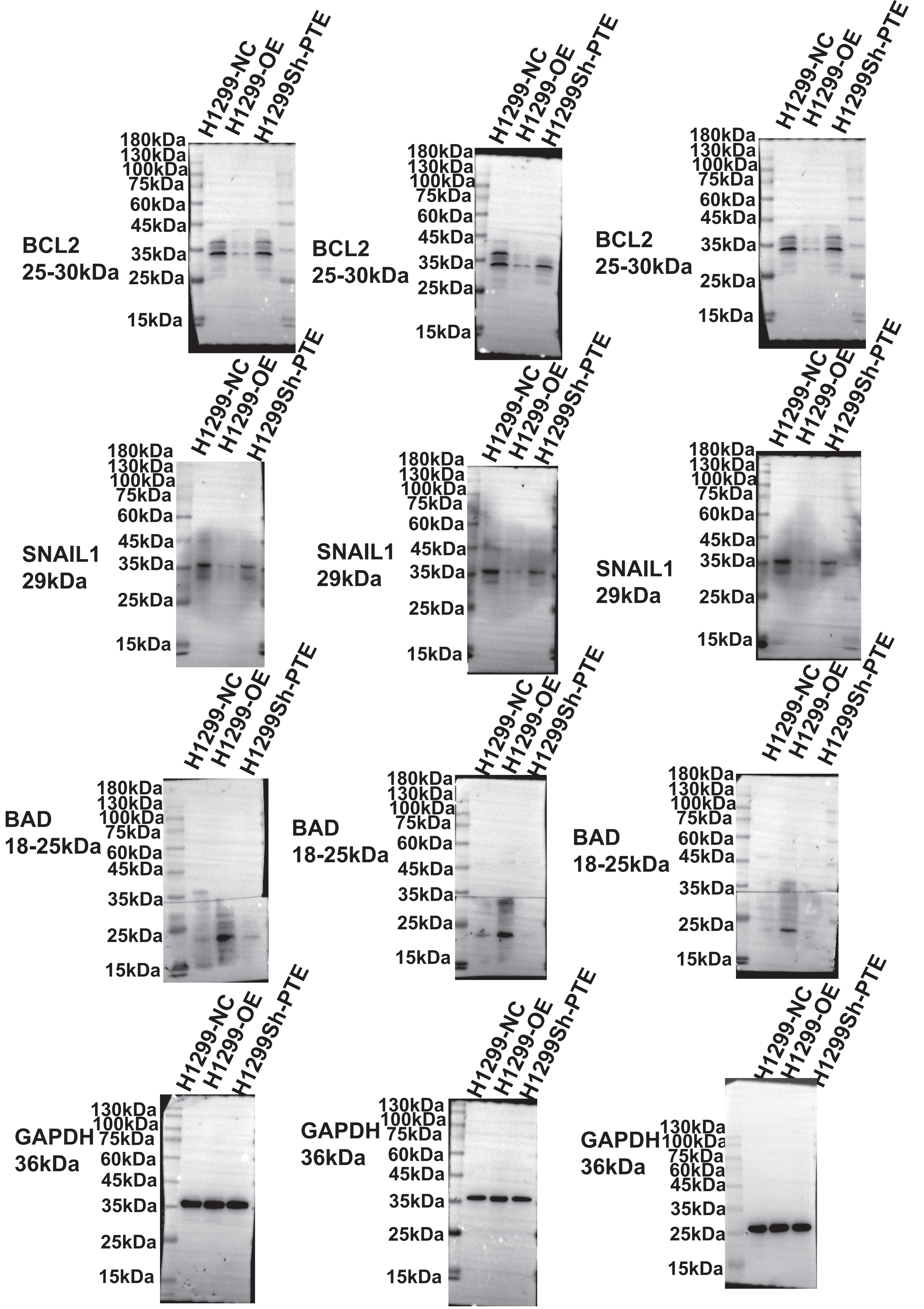

H1299-NC
H1299-OE
H1299Sh-PTE
180kDa
130kDa
100kDa
75kDa
60kDa
45kDa
35kDa
25kDa
15kDa
BCL2
25-30kDa
BCL2
25-30kDa
BCL2
25-30kDa
SNAIL1
29kDa
SNAIL1
29kDa
SNAIL1
29kDa
BAD
18-25kDa
BAD
18-25kDa
BAD
18-25kDa
GAPDH
36kDa
GAPDH
36kDa
GAPDH
36kDa
