# Supplementary material for: CDC73 suppresses lung adenocarcinoma via the PTEN/AKT pathway and exhibits biomarker and functional potential in extracellular vesicles
Source: Cell Death Discov. 2026 Aug 14;12:412. doi: 10.1038/s41420-026-03305-6 (PMC13652814; doi:10.1038/s41420-026-03305-6)
Supplement: Supplementary file 9 [file 41420_2026_3305_MOESM1_ESM.docx]

Supplementary Figure 1. CDC73 suppresses LUAD cell proliferation in vitro and alters cell-cycle progression. (A) Lentiviral transduction of A549 cells with Western blot verification of CDC73 overexpression. (B) CCK-8 assay comparing proliferation between control and CDC73-overexpressing A549 cells. (C) EdU incorporation assay assessing proliferation in control versus CDC73-overexpressing A549 cells. Scale bar, 100 μm. (D) Flow-cytometric analysis of cell-cycle distribution in A549 cells with or without CDC73 overexpression, with quantitative summary. *P < 0.05.

Supplementary Figure 2. (A) Confocal immunofluorescence imaging of A549 cells showing N-cadherin and E-cadherin signal intensities in control versus CDC73-overexpressing groups; (B) prediction of the CDC73 protein–protein interaction network using the STRING database; (C) Western blot analysis of changes in proteins within the CTNNB1/PTEN pathway following CDC73 overexpression.

Supplementary Figure 3. CDC73 promotes apoptosis of lung adenocarcinoma cells in vitro. (A) Flow-cytometric analysis of apoptosis in A549 cells with and without CDC73 overexpression, with quantitative summary. (B) Flow-cytometric analysis of apoptosis in H1299 cells with and without CDC73 overexpression, with quantitative summary. (C) Western blot analysis of apoptosis-related proteins following CDC73 overexpression, with densitometric quantification. *P < 0.05; **P < 0.01.

Supplementary Figure 4. Rescue experiments indicate that CDC73 suppresses LUAD cell proliferation and migration in vitro via PTEN and modulates cell-cycle progression. (A, B) CCK-8 assays assessing proliferation of A549 and H1299 cells in control, CDC73-overexpression, and CDC73-overexpression plus PTEN-knockdown groups. (C–E) Wound-healing assays evaluating migration in the same three groups of A549 and H1299 cells. ****P < 0.0001. (F, G) Flow-cytometric analysis of cell-cycle distribution across the three groups, with quantitative summaries. (H-J) Western blot analysis and densitometric quantification of proliferation- and migration-related proteins, as well as PTEN pathway components, in the three groups.

Supplementary Figure 5. Characterization and uptake of extracellular vesicles (EVs) derived from human plasma and LUAD cells. (A) EVs were isolated from human plasma by ultracentrifugation (plasma-exo+); Western blotting assessed EV markers (CD9, CD63, TSG101) in the isolated EVs and in exosome-depleted plasma (ED-plasma). (B, C) Western blot analysis of EV markers (TSG101, CD63, CD9) and CDC73 in EVs derived from A549NC, H1299NC, A549OE, and H1299OE cells. (D) Transmission electron microscopy showing the characteristic cup-shaped ultrastructure of plasma EVs. (E) Nanoparticle tracking analysis (NTA) indicating particle size distribution and concentration of plasma EVs. (F) GFP–CD63–labeled EVs released by lentivirally transduced H1299 cells were co-cultured with HBE cells; immunofluorescence imaging demonstrates EV uptake by HBE cells. F-actin was labeled with phalloidin (CL594). Scale bar, 100 μm.

Supplementary Figure 6. Following co-culture of HBE cells with EVs from lung adenocarcinoma cells (A549NC and A549OE), HBE cells in the OE group exhibited reduced proliferation and increased apoptosis compared with the NC group. (A) Flow-cytometric analysis of cell-cycle distribution in HBE cells after co-culture, with quantitative summary. (B) EdU assay assessing proliferative changes in HBE cells after co-culture. (C, D) Western blot analysis and quantification of cell-cycle–related proteins in HBE cells after co-culture. *P < 0.05; ***P < 0.001. (E) Flow-cytometric analysis of apoptosis in HBE cells following co-culture. (F) Western blot analysis and quantification of apoptosis-related proteins in HBE cells after co-culture with EVs from A549NC and A549OE cells. *P < 0.05; **P < 0.01; ***P < 0.001.
